# Interlocking Pseudorotaxanes through Borane- and Boronic Ester-Mediated Carbon–Carbon Bond Formation

**DOI:** 10.1021/acs.orglett.5c01233

**Published:** 2025-04-30

**Authors:** Jia-Jyun Jian, Min-Xuan Zhang, Yi-Hung Liu, Sheng-Hsien Chiu

**Affiliations:** Department of Chemistry, National Taiwan University, No. 1, Sec. 4, Roosevelt Road, Taipei 106, Taiwan

## Abstract

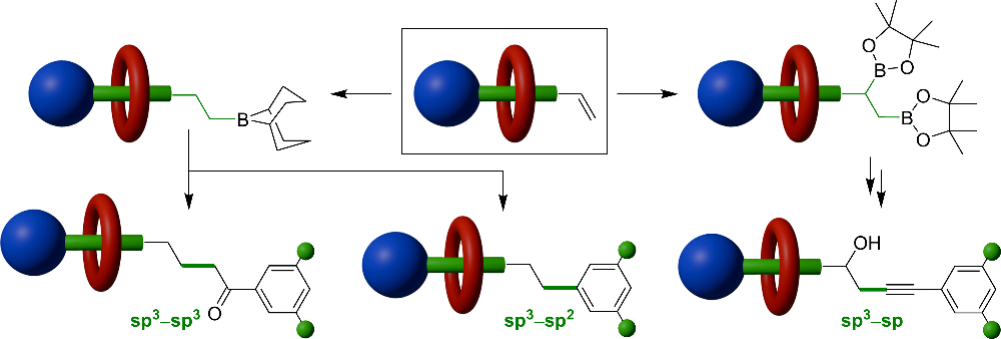

Employing 9-BBN and pinacol boronic ester as surrogate stoppers, pseudorotaxanes presenting terminal alkenes can be transformed into organoborane or organoboronate rotaxanes, respectively. Various boron-based reactions can then be used to replace these temporary stoppers with bulky derivatives of aromatic bromides, α-bromo ketones, or 1-bromo-1-alkynes, facilitating the selective formation of sp^3^–sp^2^, sp^3^–sp^3^, and sp^3^–sp carbon–carbon single bonds, respectively.

The interlocked structures and switchable properties of rotaxanes have made them functional materials with applications in such fields as sensing,^1^ catalysis,^2^ and gelation.^3^ Over the past three decades, many strategies have been developed for their synthesis, including threading-followed-by-stoppering,^4^ slippage,^5^ clipping,^6^ threading-followed-by-swelling,^7^ threading-followed-by-ring-shrinking,^8^ and active-template synthesis.^9^ Among them, threading-followed-by-stoppering has emerged as the most popular, owing to its relative synthetic ease and structural flexibility. Nevertheless, the range of stoppering reactions is limited by the need for solvents and/or reagents that ensure the stability of the pseudorotaxane structures.

Previously reported stoppering reactions allow the introduction of diverse functionalities—including ester,^10^ amide,^11^ imine,^12^ and triazole^13^ groups—as links between the linear component’s termini and the stopper units in the resulting rotaxanes. Despite the importance to organic chemistry of reactions that form carbon–carbon single bonds, and the potential to use their high stability to facilitate the postsynthesis of rotaxanes, few methods are available for interlocking pseudorotaxanes through the formation of C–C single bonds (Figure 1).^14^ This situation is due to many of the reactions leading to C–C single bond formation involving strong nucleophiles or bases, or polar solvents, which tend to destabilize most pseudorotaxanes.

**Figure 1:**
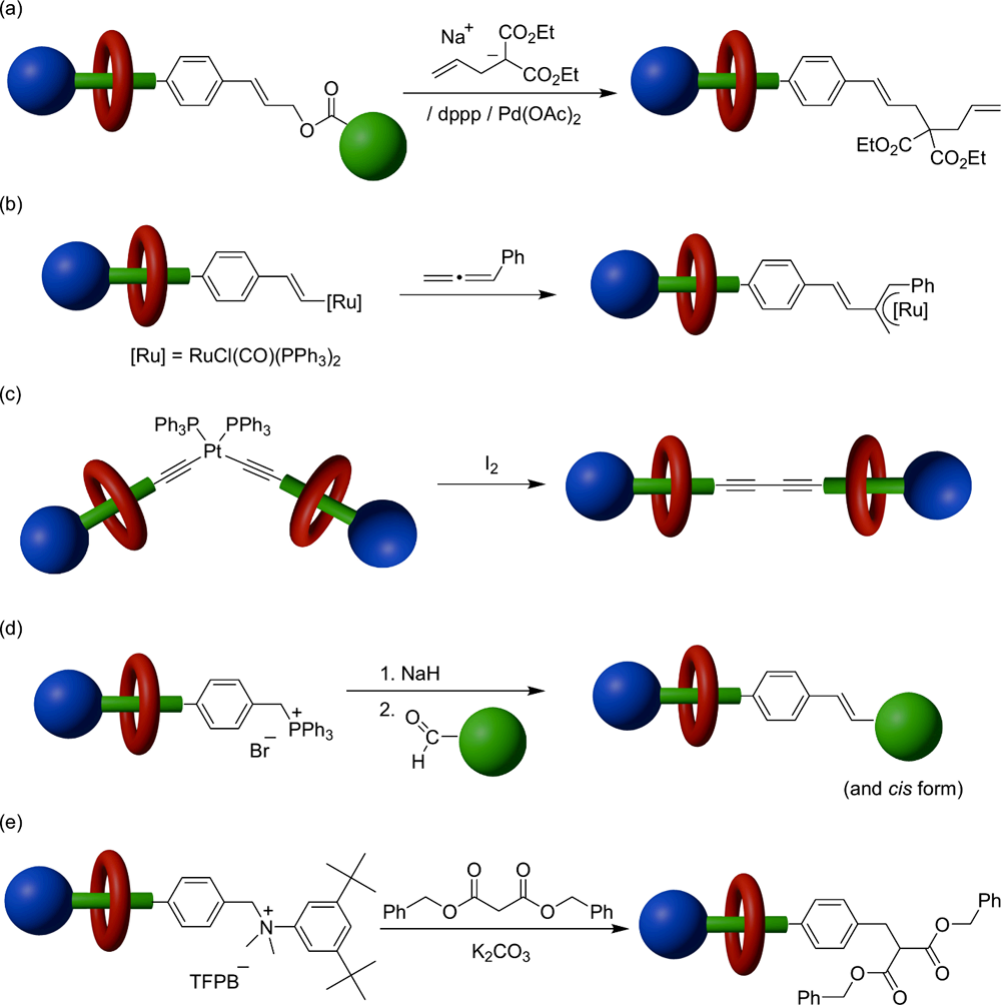
Reactions that interlock surrogate rotaxanes through the formation of C–C bonds in the (a–c) presence and (d, e) absence of transition metals.

Successful examples of stoppering with C–C single bond formation have typically involved the preformation of an interlocked intermediate (i.e., a surrogate rotaxane) that prevents the macrocycle from dethreading under the potentially harsher conditions required for C–C bond formation.^15^ By complexing a transition metal ion catalyst to the interlocked intermediates, rotaxanes with newly formed C–C single bond linkages can be synthesized through Tsuji–Trost allylation^16^ (Figure 1a), alkyne hydroruthenation followed by allene insertion^17^ (Figure 1b), and I_2_-promoted coupling of *cis*-alkyne–Pt–alkyne complexes^18^ (Figure 1c). In examples where no transition metal ion is needed, Wittig reactions^19^ (Figure 1d) and malonate alkylations^4b^ (Figure 1e) allow the indirect (hydrogenation of the formed C=C double bond) and direct production of C–C single bonds, respectively, from triphenylphosphonium- and trialkylanilinium-stopped surrogate rotaxanes, respectively.

New practical and robust methods for interlocking the components of pseudorotaxanes are always welcome, especially if they allow for the efficient formation of rotaxanes with different types of C–C single bond linkages. Herein, we report the use of 9-borabicyclo[3.3.1]nonane (9-BBN) and bis(pinacolato)diboron (B_2_pin_2_) as surrogate stoppers for the efficient interlocking of pseudorotaxanes presenting alkene termini, and the reactions of the resulting organoborane and organoboronic ester rotaxanes with bulky versions of an aromatic bromide, an α-bromo ketone, and a 1-bromo-1-alkyne, in the presence or absence of transition metals, to form rotaxanes with sp^3^–sp^2^-, sp^3^–sp^3^-, and sp^3^–sp-hybridized C–C single bond linkages, respectively.^20^

A single type of stoppering reaction would not always be practical for every host–guest recognition system because the noncovalent interactions that stabilize their pseudorotaxanes would respond differently under the reaction conditions. Therefore, any practical general method for interlocking would best be tested using relatively weakly associated recognition systems—and particularly those more sensitive to the reaction conditions (i.e., to the presence of nucleophiles, anions, and extreme solvent polarity). Here, we chose the monopyridinium/bis-*p*-xylyl[26]crown-6 (BPX26C6)^21^ and urea/Na^+^/BPX26C6^22^ recognition systems. In theory, concerted additions, which release no molecular fragments into the reaction mixture, would minimize disturbance from external chemical additives to the pseudorotaxane complexes. Therefore, we examined the addition of bulky 9-BBN to terminal alkenes in the preparation of surrogate rotaxanes. We prepared the semidumbbell-shaped pyridinium salt **1**·TFPB and the urea **2**, both presenting alkene termini, through N- and O-alkylations of the pyridine **3** and the urea **4**, respectively, with 6-bromo-1-hexene (Scheme 1).

**Scheme 1:**
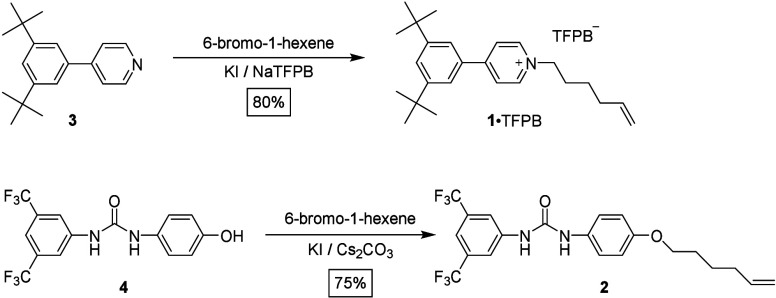
Synthesis of Semidumbbell-Shaped Threadlike Species Presenting Alkene Termini

We added 9-BBN dimer (0.22 M) to an equimolar (0.4 M) solution of threadlike salt **1**·TFPB and macrocycle BPX26C6 in toluene-*d*_8_ and then heated the mixture at 60 °C (Scheme 2). By diluting aliquots of the solution over time, ^1^H NMR spectroscopy (toluene-*d*_8_) revealed the diminution of the alkene signals (Figure 2a) and the growth of those for the interlocked macrocycle; these changes were complete after 16 h, suggesting successful formation of the rotaxane **5**·TFPB through hydroboration of the alkene terminus of the pseudorotaxane BPX26C6⊃**1**·TFPB. Based on integration of signals of the aromatic protons of the interlocked (δ = 6.52) and free (br, δ = 6.62–6.85) macrocycles, we estimated the reaction yield to be approximately 80%. Because alkyl-9-BBN compounds are generally highly air-sensitive,^23^ we applied the crude rotaxane **5**·TFPB directly in the next step, rather than purifying it. Using Pd(dppf)Cl_2_ to catalyze the coupling of the crude interlocked organoborane **5**·TFPB and 1-bromo-3,5-di-*tert*-butylbenzene (**6**) in DMF,^24^ we obtained the stopper-exchanged product, the rotaxane **7**·TFPB, in an overall yield (for sequential hydroboration/Suzuki coupling) of 70% after column chromatography. Thus, using 9-BBN to make an interlocked organoborane surrogate, followed by Suzuki coupling to generate a C–C (sp^3^–sp^2^-hybridized) bond, is a feasible and reasonably efficient method for linking a pseudorotaxane’s terminal alkene and a bulky aromatic bromide.

**Figure 2:**
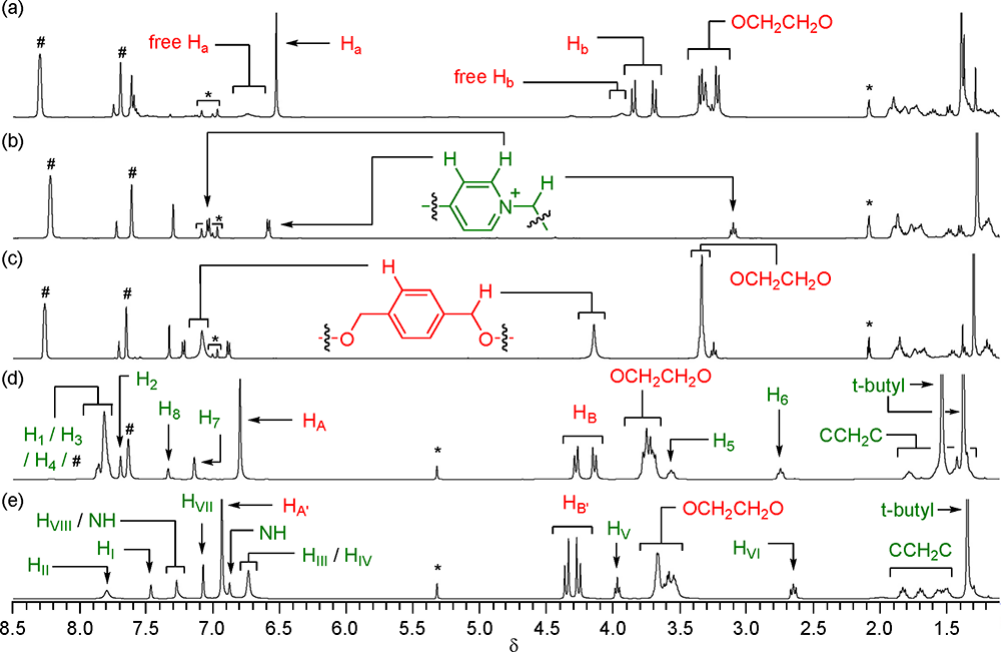
^1^H NMR spectra (400 MHz, 298 K) of (a) the sample obtained after mixing **1**·TFPB (0.4 M), BPX26C6 (0.4 M), and 9-BBN dimer (0.22 M) in toluene and heating at 60 °C for 16 h (toluene-*d*_8_); (b) the sample obtained after mixing **1**·TFPB (0.4 M) and 9-BBN dimer (0.22 M) and heating at 60 °C for 16 h (toluene-*d*_8_); (c) the sample obtained after mixing 1 equiv of BPX26C6 with the solution in (b) and heating at 60 °C for 16 h (toluene-*d*_8_); (d) the rotaxane **7**·TFPB (CD_2_Cl_2_); and (e) the rotaxane **9** (CD_2_Cl_2_). Asterisks: signals from residual solvent. Hashtag: signals from the TFPB^–^ anion.

**Scheme 2:**
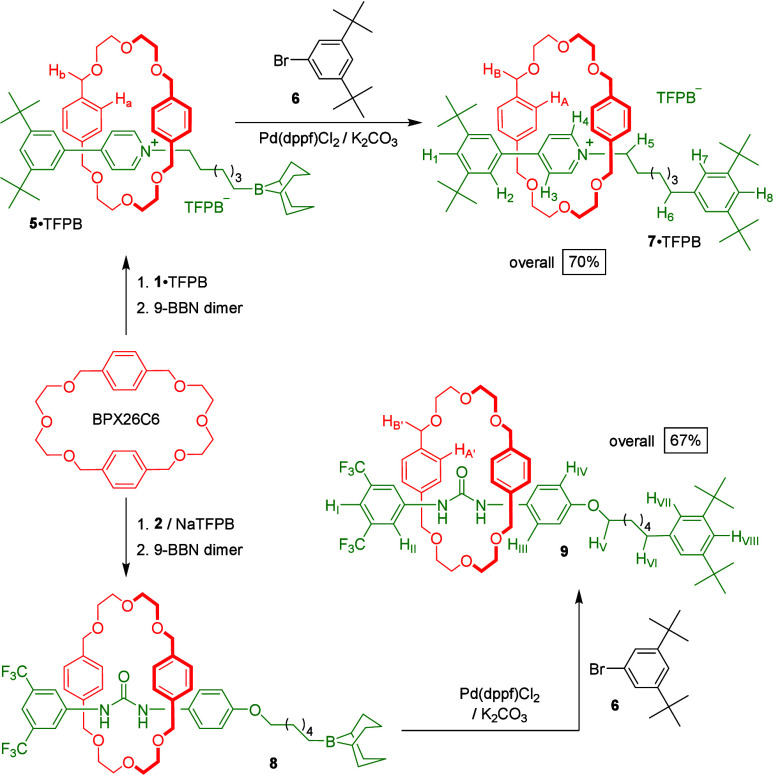
Synthesis of 9-BBN-Stoppered Rotaxanes, and Their Exchange with New Stoppers to Form sp^3^–sp^2^-Hybridized C–C Bond Linkages

We obtained single crystals suitable for X-ray crystallography after slowly evaporating a solution of [2]rotaxane **7**·TFPB in CH_2_Cl_2_/hexanes (1:2). The solid state structure^25^ (Figure 3) confirms the [2]rotaxane geometry of **7**·TFPB, with the interlocked macrocycle encircling the pyridinium unit. This structure is stabilized by multiple C–H**···**O hydrogen bonds between the hydrogen atoms adjacent to the pyridinium nitrogen and the macrocycle’s oxygen atoms, as well as by an aromatic stacking interaction between the pyridinium unit and a xylene ring (centroid–centroid distance: 3.587 Å).

**Figure 3:**
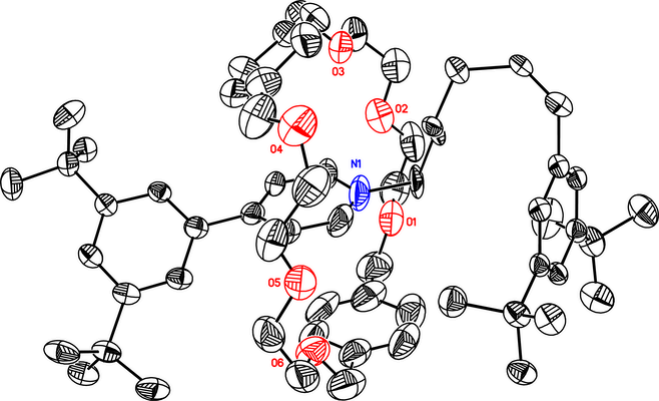
ORTEP representation of the [2]rotaxane **7**·TFPB with 50% probability atomic displacement parameters. Anions, hydrogen atoms, and solvent molecules have been omitted for clarity.

Although the successful synthesis of rotaxane **7**·TFPB from surrogate rotaxane **5**·TFPB in polar DMF implied that 9-BBN is an appropriate stopper for interlocking BPX26C6, we examined whether 9-BBN was possibly a slippage stopper for BPX26C6 in **5**·TFPB. First, we heated a toluene-*d*_8_ solution of threadlike salt **1**·TFPB (0.4 M) and 9-BBN dimer (0.22 M) at 60 °C for 16 h. The ^1^H NMR spectrum of the sample obtained after diluting an aliquot of this mixture revealed (Figure 2b) no alkene signals, suggesting that the hydroboration had been successful. Next, we added BPX26C6 (0.4 M) to the reaction mixture and heated it to 60 °C for 16 h. No signals for complexation appeared in the ^1^H NMR spectrum (Figure 2c), suggesting that the rotaxane **5**·TFPB had indeed been constructed through threading-followed-by-stoppering with 9-BBN functioning as a real stopper for BPX26C6 under the reaction conditions.

We applied the same synthetic method to efficiently interlock [(BPX26C6·Na)⊃**2**], a pseudorotaxane that is highly sensitive to anions and solvent polarity. Using 9-BBN dimer (0.22 M) to hydroborate the alkene terminus of the pseudorotaxane formed from an equimolar (0.4 M) mixture of the threadlike urea **2**, BPX26C6, and NaTFPB, followed by Suzuki coupling between the resulting surrogate rotaxane **8** and the stopper **6** in the presence of Pd(dppf)Cl_2_, we isolated the rotaxane **9** with a C–C (sp^3^–sp^2^-hybridized) single bond linking the pseudorotaxane’s terminus and the stopper, in 67% yield (Scheme 2).

Having confirmed that hydroboration of a pseudorotaxane’s terminal C=C double bond with 9-BBN and subsequent Pd-catalyzed coupling between the resulting organoborane surrogate rotaxane and a bulky aromatic bromide could afford a rotaxane with a new sp^3^–sp^2^-hybridized C–C single bond, we turned our attention to a transition-metal-free method for C–C single bond formation. Brown et al. demonstrated that organoboranes can react with α-bromo esters under basic conditions;^26^ we suspected that an organoborane surrogate rotaxane should react similarly with a bulky α-bromoacetophenone to afford a rotaxane with an sp^3^–sp^3^-hybridized C–C bond linkage, without the need for a transition metal. We prepared the surrogate pyridinium-based rotaxane **5**·TFPB and mixed the crude product with α-bromo ketone **10** in the presence of a base, potassium 2,6-di-*tert*-butylphenolate (**11**), in THF, affording rotaxane **12**·TFPB in 39% yield after chromatography (Scheme 3). Similarly, reacting the crude surrogate urea-based rotaxane **8** with the α-bromo ketone **10** in the presence of base **11** afforded the rotaxane **13** in 50% yield. Therefore, depending on the synthetic design, the 9-BBN stoppers of the surrogate rotaxanes **5**·TFPB and **8** can be replaced by other bulky stoppers to form sp^3^–sp^2^- and sp^3^–sp^3^-hybridized C–C bond linkages in the presence and absence, respectively, of a transition metal catalyst.

**Scheme 3:**
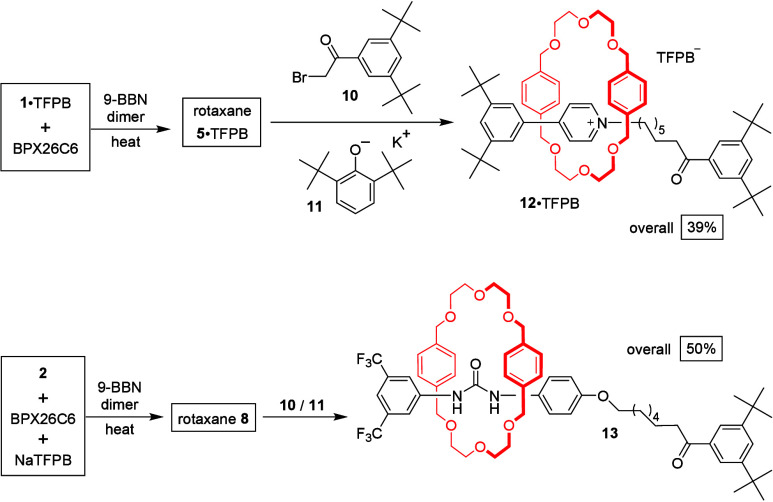
Forming sp^3^–sp^3^-Hybridized C–C Bonds through Reactions of the 9-BBN-Stoppered Surrogate Rotaxanes

Next, we replaced 9-BBN with a bulky boronic ester so that we could isolate the surrogate rotaxanes as air-stable products. Here, we applied organocatalytic diboration^27^ to interlock a pseudorotaxane presenting an alkene terminus. After mixing the threadlike salt **1**·TFPB (0.25 M), BPX26C6 (0.25 M), bis(pinacolato)diboron (B_2_pin_2_, 0.50 M), Cs_2_CO_3_ (2 equiv), MeOH (1 M), and TBA·TFPB (0.25 M) in CH_2_Cl_2_, we isolated the rotaxane **14**·TFPB in 45% yield (Scheme 4).^28^ Similarly, we reacted threadlike urea **2** (0.25 M), NaTFPB (0.75 M), BPX26C6 (0.75 M), bis(pinacolato)diboron (B_2_pin_2_, 0.50 M), MeOH (0.75 M), and 1,8-bis(dimethylamino)naphthalene (proton sponge, 0.125 M) in CH_2_Cl_2_ and obtained a 75% yield of the rotaxane **15**.

**Scheme 4:**
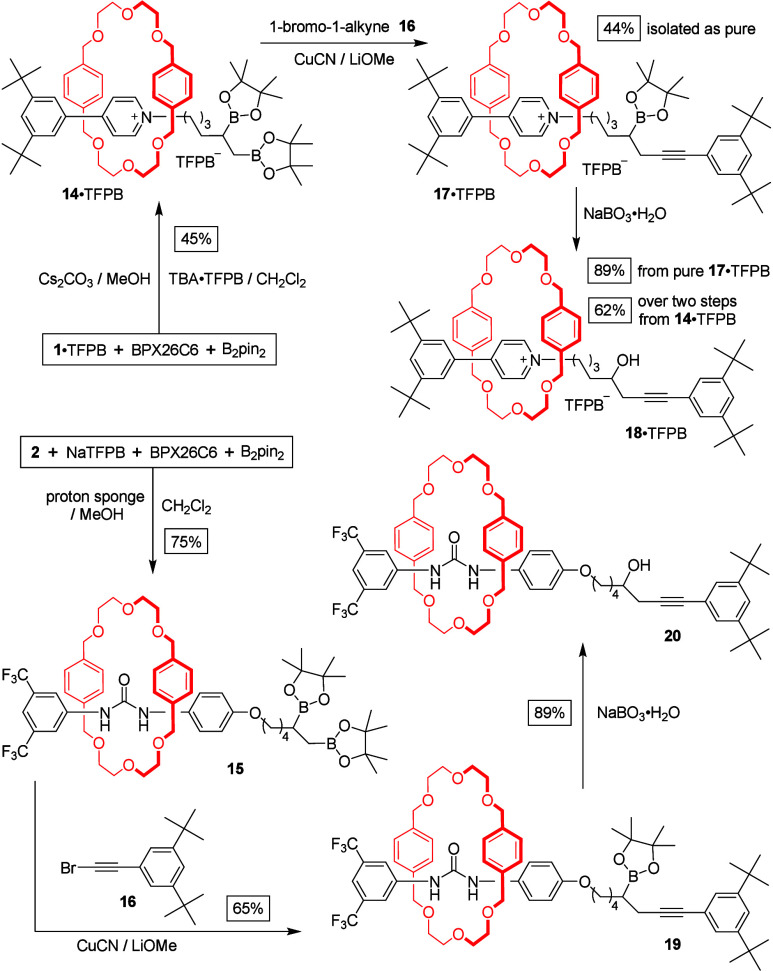
Synthesis of Bis(Boronate) Surrogate Rotaxanes and Their Exchange with New Stoppers to Form sp^3^–sp-Hybridized C–C Bond Linkages

Although alkyl pinacol boronates are generally much less reactive than alkyl boranes when used as substrates in cross-couplings, the reactivity of the primary organoboronate in a 1,2-bis(boronate) is enhanced by vincinal O–B chelation, which increases the Lewis acidity^29^ and allows the reactions to proceed efficiently under relatively benign conditions. By reacting the rotaxane **14**·TFPB (0.2 M) with a mixture of CuCN (60 mM), lithium methoxide (0.6 M), and the 1-bromo-1-alkyne **16** (0.24 M),^30^ we obtained the pyridinium-based rotaxane **17**·TFPB (44% yield),^31^ featuring a newly generated sp^3^–sp-hybridized C–C single bond between the erstwhile threadlike component and the stopper. We removed the remaining boronic ester group in **17**·TFPB through NaBO_3_·H_2_O-mediated oxidation, providing alcohol rotaxane **18**·TFPB in 89% yield. Applying the same transformation to the urea-based rotaxane **15** afforded the boronic ester rotaxane **19** (65% yield), which we then converted to the alcohol rotaxane **20** using NaBO_3_·H_2_O, also in 89% yield.

Using 9-BBN and pinacol boronic ester to form surrogate stoppers, we have transformed pseudorotaxanes presenting alkene termini into rotaxanes by creating C–C single bond linkages to proper stoppers. This approach might be generally useful because terminal alkenes are readily prepared synthetically and because boron-based reactions allow the installation of C–C single bonds, with sp^3^–sp^3^, sp^3^–sp^2^, and sp^3^–sp hybridization, through distinct stopper exchange processes, leaving room for further installation of various functionalities on the threadlike components. We believe that such stoppering methods will aid in the design and synthesis of interlocked (macro)molecules and switches.

## Data Availability

The data underlying this study are available in the published article and its Supporting Information.

## Supporting Information Available

Synthetic procedures, characterization data and NMR spectra of the rotaxanes (PDF)
