# Supplementary material for: Interlocking Pseudorotaxanes through Borane- and Boronic Ester-Mediated Carbon–Carbon Bond Formation
Source: Org Lett. 2025 Apr 30;27(18):4772–6. doi: 10.1021/acs.orglett.5c01233 (PMC12070462; doi:10.1021/acs.orglett.5c01233)
Supplement: Supplementary file 1 — ol5c01233_si_001.pdf [file ol5c01233_si_001.pdf]

Supplementary Material

Interlocking Pseudorotaxanes through Borane- and Boronic Ester-Mediated Carbon–Carbon Bond Formation

Jia-Jyun Jian, Min-Xuan Zhang, Yi-Hung Liu, and Sheng-Hsien Chiu*

Department of Chemistry, National Taiwan University, No. 1, Sec. 4, Roosevelt Road, Taipei 106, Taiwan

---

Data Page Number

Experimental procedures and characterization data for new compounds ···· S2–S19

^{1}H and ^{13}C NMR spectra of the rotaxanes, the dumbbell-shaped molecules, and their synthetic precursors ·· S20–S55

2D COSY and ROESY NMR spectra of the rotaxanes························ S56–S78

^{1}H NMR spectra of the 9-BBN hydroboration of **1**·TFPB, performed with or without BPX26C6 ·· S79

ORTEP representation of [2]rotaxane **7**·TFPB ································· S80

Crystallographic data and experimental details for [2]rotaxane **7**·TFPB ···· S81

General Methods: All glassware, stirrer bars, and needles were either oven-dried or treated with a heat gun under high vacuum before use. Unless otherwise specified, all reactions were performed under an inert nitrogen atmosphere using commercially available chemicals. Reactions requiring elevated temperatures were conducted using a sand or oil bath, while those sensitive to moisture or oxygen were performed inside an MBRAUN UNIlab Pro glovebox filled with argon. When necessary, chemical reagents were dehydrated using a BUCHI B-585 glass oven equipped with a drying pistol containing P_2O_5. Column chromatography was performed using Kieselgel 60 SiO_2 silica gel (70–230 mesh, Merck), LiChroprep RP-18 silica gel (40–63 μm, Merck), or Chromatorex DIOL silica gel (MB100-40/75, Fuji Silysia). Thin-layer chromatography (TLC) was performed on glass plates coated with the corresponding silica gel (thickness: 0.25 mm) and fluorescent indicator F_{254}. Melting points were determined using a Fargo MP-2D melting point apparatus. NMR spectra were recorded in deuterated solvents, which served as both the lock and shim medium. Residual solvent peaks were used as internal standards: $CDCl_3$ (^{1}H: δ = 7.24 ppm; ^{13}C: δ = 77.0 ppm), CD_2Cl_2 (^{1}H: δ = 5.32 ppm; ^{13}C: δ = 53.8 ppm), and $(CD_3)_2CO$ (^{1}H: δ = 2.05 ppm; ^{13}C: δ = 29.8 ppm). Structural assignments were made with additional information from gCOSY, gHSQC, and gHMBC experiments. High-resolution mass spectrometry (HR-MS) was performed using a Bruker micrOTOF-QII or Thermo Orbitrap QE Plus instrument. High-resolution mass spectrometry (HRMS) was performed using a Sciex QStar Elite Q-TOF or Bruker microTOF-QII (Q-TOF) instrument. X-ray crystallographic data were collected using an Agilent Xcalibur [Atlas, Gemini Enhance (Mo) X-ray Source] diffractometer with ω scans at 100(2) K. Integration and reduction of the data were performed using CrysAlis PRO software (Agilent, 2014). Absorption corrections were applied to the data using Multi-scan software (CrysAlis PRO; Agilent, 2014). SHELXS-2014/7 software was applied to solve the structures by direct methods; subsequent refinement and extension were also performed using SHELXL-2014/7.

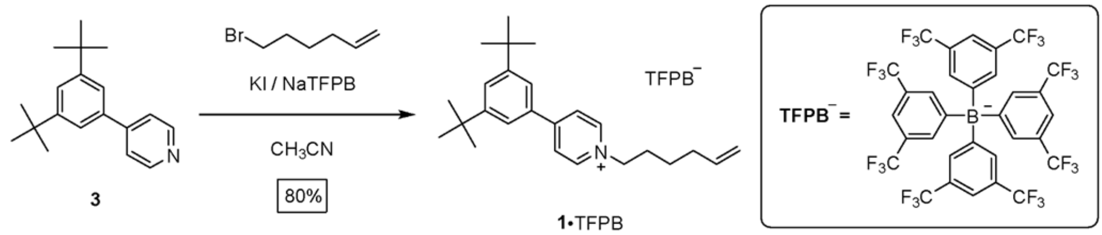

Pyridinium salt 1·TFPB: A CH_3CN (12.5 mL) solution of the pyridine **3**[1] (500 mg, 1.87 mmol), 6-bromo-1-hexene (500 μL, 3.74 mmol), potassium iodide (80.0 mg, 0.467 mmol), and NaTFPB (1.66 g, 1.87 mmol) was sealed in a pressure tube and stirred at 80 °C for 72 h. After cooling to room temperature, the mixture was

concentrated. The residue was partitioned between CH_2Cl_2 (30 mL) and H_2O (50 mL), and the aqueous layer was extracted with CH_2Cl_2 (3 × 20 mL). The combined organic phases were dried ($MgSO_4$) and concentrated. The residue was purified chromatographically [SiO_2; EtOAc/hexane (2:8) and then CH_2Cl_2/hexane (gradient from 5:5 to 10:0)] to afford the pyridinium salt **1**·TFPB as a white solid (1.81 g, 80%). M.p. = 117–118 °C; ^{1}H NMR (400 MHz, $CDCl_3$): δ = 8.06–7.99 (m, 4H), 7.76 (t, J = 1.6 Hz, 1H), 7.68 (s, 8H), 7.51–7.46 (m, 6H), 5.70–5.58 (m, 1H), 5.02–4.92 (m, 2H), 4.12 (t, J = 7.6 Hz, 2H), 2.08–2.00 (m, 2H), 1.89–1.79 (m, 2H), 1.39–1.29 (m, 20H); ^{13}C NMR (100 MHz, CD_2Cl_2): δ = 162.6 (q, $^1J_{CB}$ = 49.6 Hz), 160.7, 154.2, 143.3, 137.4, 135.6, 133.3, 130.4–129.3 (m), 128.8, 126.5, 125.4 (q, $^1J_{CF}$ = 270.8 Hz), 123.0, 118.6–118.1 (m), 116.4, 62.4, 35.7, 33.2, 31.5, 31.2, 25.6; HR-MS (ESI): calcd for [**1**]$^+$, $C_{25}H_{36}N^+$: m/z 350.2842; found 350.2831.

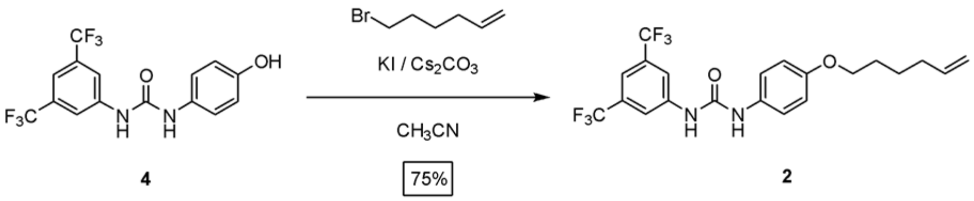

4
2

Urea 2: A mixture of the urea **4**[2] (1.09 g, 2.99 mmol), 6-bromo-1-hexene (481 μL, 3.60 mmol), potassium iodide (598 mg, 3.60 mmol), and Cs_2CO_3 (1.17 g, 3.59 mmol) in CH_3CN (30 mL) was sealed in a pressure tube and stirred at 80 °C for 72 h. After cooling to room temperature, the mixture was concentrated. The residue was partitioned between CH_2Cl_2 (50 mL) and H_2O (100 mL), and the aqueous layer was extracted with CH_2Cl_2 (3 × 50 mL). The combined organic phases were dried ($MgSO_4$) and concentrated. The residue was purified chromatographically [SiO_2; acetone/hexane (2:8)] to afford the solid urea **2**, which was further washed with $CHCl_3$/hexane (3:7) and dried under vacuum to give a white solid (1.00 g, 75%). M.p. = 160–161 °C; ^{1}H NMR (400 MHz, $(CD_3)_2CO$): δ = 8.68 (s, 1H), 8.25–8.08 (m, 3H), 7.57 (s, 1H), 7.42 (d, J = 8.8 Hz, 2H), 6.88 (d, J = 8.8 Hz, 2H), 5.95–5.76 (m, 1H), 5.08–4.90 (m, 2H), 3.96 (t, J = 6.4 Hz, 2H), 2.20–2.07 (m, 2H), 1.85–1.68 (m, 2H), 1.63–1.49 (m, 2H); ^{13}C NMR (100 MHz, $(CD_3)_2CO$): δ = 156.2, 153.4, 143.1, 139.5, 132.7, 132.4 (q, $^2J_{CF}$ = 32.7 Hz), 124.4 (q, $^1J_{CF}$ = 270.3 Hz), 122.1, 119.1–118.7 (m), 115.4, 115.4–115.1 (m), 115.0, 68.5, 34.1, 29.5, 26.1; HR-MS (ESI): calcd for [**2** + H]$^+$, $C_{21}H_{21}F_6N_2O_2^+$: m/z 447.1502; found 447.1496.

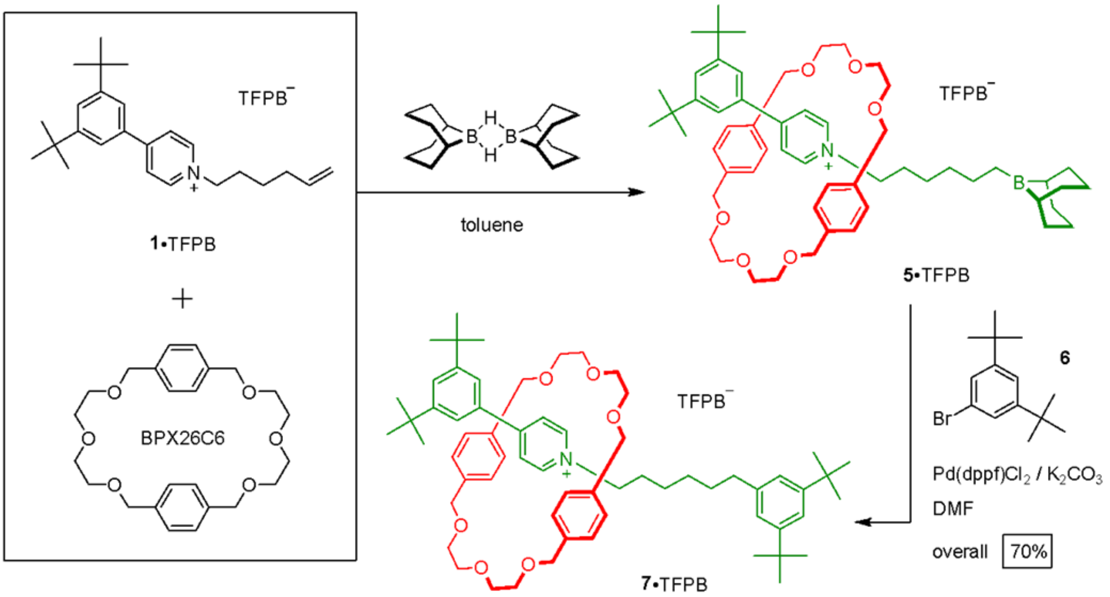

Pyridinium rotaxane 7·TFPB: In a glovebox, a mixture of the pre-dried pyridinium salt **1**·TFPB (146 mg, 0.120 mmol) and BPX26C6[3] (50.0 mg, 0.120 mmol) in anhydrous toluene (0.3 mL) was stirred at 60 °C. When the mixture became homogeneous, the solution was treated with 9-BBN dimer (16.1 mg, 0.132 mmol as monomer) and then the resulting mixture was stirred at 60 °C for 18 h to afford the 9-BBN-stoppered rotaxane **5**·TFPB. After cooling to room temperature, the mixture was diluted with DMF (0.9 mL) and treated with Pd(dppf)Cl_2 (4.39 mg, 6.00 μmol), K_2CO_3 (33.2 mg, 0.240 mmol), and the bromide **6** (38.8 mg, 0.144 mmol). After stirring at 60 °C for 6 h, the mixture was cooled to room temperature and moved from the glovebox to a fume hood. The mixture was diluted with EtOAc (20 mL) and washed with water (3 × 20 mL). The organic phase was dried ($MgSO_4$) and concentrated. The residue was purified chromatographically [SiO_2; CH_2Cl_2/hexane (gradient from 5:5 to 9:1)] to afford the pyridinium rotaxane **7**·TFPB as a pale-yellow solid (153 mg, 70%). M.p. = 115–116 °C; ^{1}H NMR (400 MHz, CD_2Cl_2): δ = 7.91–7.72 (m, 13H), 7.69 (s, 2H), 7.63 (s, 4H), 7.33 (s, 1H), 7.14 (s, 2H), 6.80 (s, 8H), 4.28 (d, J = 10.0 Hz, 4H), 4.14 (d, J = 10.0 Hz, 4H), 3.83–3.62 (m, 16H), 3.62–3.51 (m, 2H), 2.74 (t, J = 6.8 Hz, 2H), 1.85–1.72 (m, 2H), 1.64–1.47 (m, 22H), 1.47–1.33 (m, 20H); ^{13}C NMR (100 MHz, CD_2Cl_2): δ = 162.3 (q, $^1J_{CB}$ = 49.5 Hz), 155.0, 153.1, 151.3, 144.5, 142.0, 137.4, 135.3, 134.0, 130.1–128.9 (m), 128.9, 126.9, 125.3, 125.1 (q, $^1J_{CF}$ = 270.8 Hz), 123.0, 122.5, 120.3, 118.3–117.7 (m), 74.0, 71.6, 70.8, 60.2, 36.9, 35.6, 35.1, 32.2, 31.7, 31.7, 30.3, 29.6, 27.1; HR-MS (ESI): calcd for [**7**]$^+$, $C_{63}H_{90}NO_6^+$: m/z 956.6763; found 956.6768.

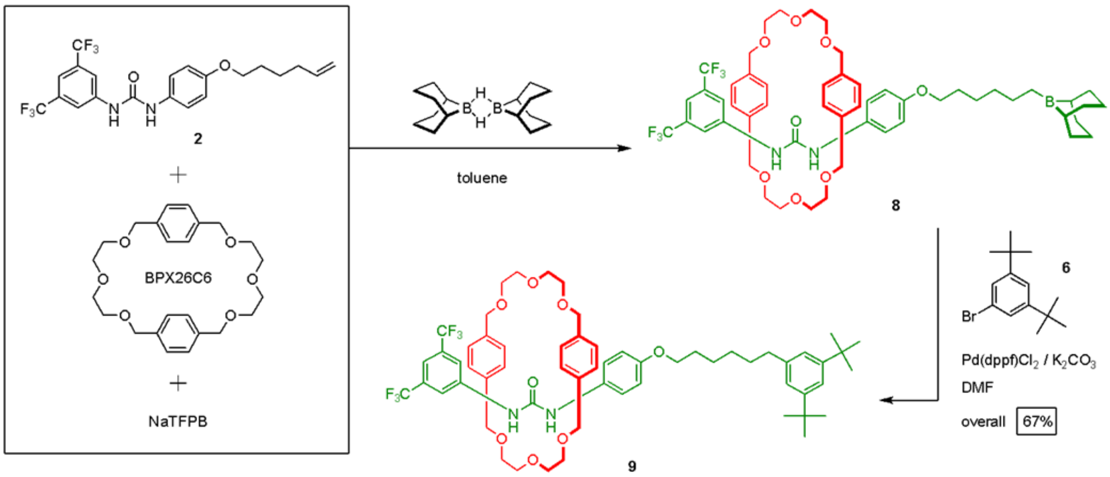

Urea rotaxane 9: In a glovebox, the pre-dried urea **2** (53.6 mg, 0.120 mmol), BPX26C6 (50.0 mg, 0.120 mmol), and NaTFPB (pre-ground as a fine powder; 106 mg, 0.120 mmol) were mixed in anhydrous toluene (0.3 mL). After stirring at 60 °C for 18 h, the mixture became homogeneous. 9-BBN dimer (16.1 mg, 0.132 mmol as monomer) was added and the mixture was stirred at 60 °C for another 18 h to afford the 9-BBN-stoppered rotaxane **8**. After cooling to room temperature, the mixture was diluted with DMF (0.9 mL) and treated with $Pd(dppf)Cl_2$ (4.39 mg, 6.00 μmol), K_2CO_3 (33.2 mg, 0.240 mmol), and the bromide **6** (38.8 mg, 0.144 mmol); this mixture was then stirred at 60 °C for 6 h. After cooling to room temperature, the mixture was moved from the glovebox to a fume hood. The mixture was diluted with EtOAc (20 mL) and washed with water (3 × 20 mL). The organic phase was dried ($MgSO_4$) and concentrated. The residue was purified chromatographically [SiO_2; EtOAc/hexane (2:8)] to afford the urea rotaxane **9** as a sticky colorless liquid (85.2 mg, 67%). ^{1}H NMR (400 MHz, CD_2Cl_2): δ = 7.78 (br, 2H), 7.46 (s, 1H), 7.33–7.16 (m, 2H), 7.07 (s, 2H), 7.00–6.82 (m, 9H), 6.73 (br, 4H), 4.34 (d, J = 11.2 Hz, 4H), 4.25 (d, J = 11.2 Hz, 4H), 3.96 (t, J = 6.4 Hz, 2H), 3.73–3.45 (m, 16H), 2.64 (t, J = 8.0 Hz, 2H), 1.88–1.77 (m, 2H), 1.75–1.63 (m, 2H), 1.62–1.44 (m, 4H), 1.34 (s, 18H); ^{13}C NMR (100 MHz, CD_2Cl_2): δ = 155.0, 151.0, 150.9, 142.5, 142.4, 137.4, 132.2 (br), 131.4 (q, $^2J_{CF}$ = 32.6 Hz), 128.6, 124.2 (q, $^1J_{CF}$ = 270.8 Hz), 123.0, 121.6 (br), 119.9, 118.7 (br), 114.6, 114.1 (br), 73.6, 71.1, 69.3, 68.7, 36.9, 35.0, 32.3, 31.7, 29.8, 29.8, 26.4; HR-MS (ESI): calcd for [**9** + H]$^+$, $C_{59}H_{75}F_6N_2O_8^+$: m/z 1053.5422; found 1053.5408.

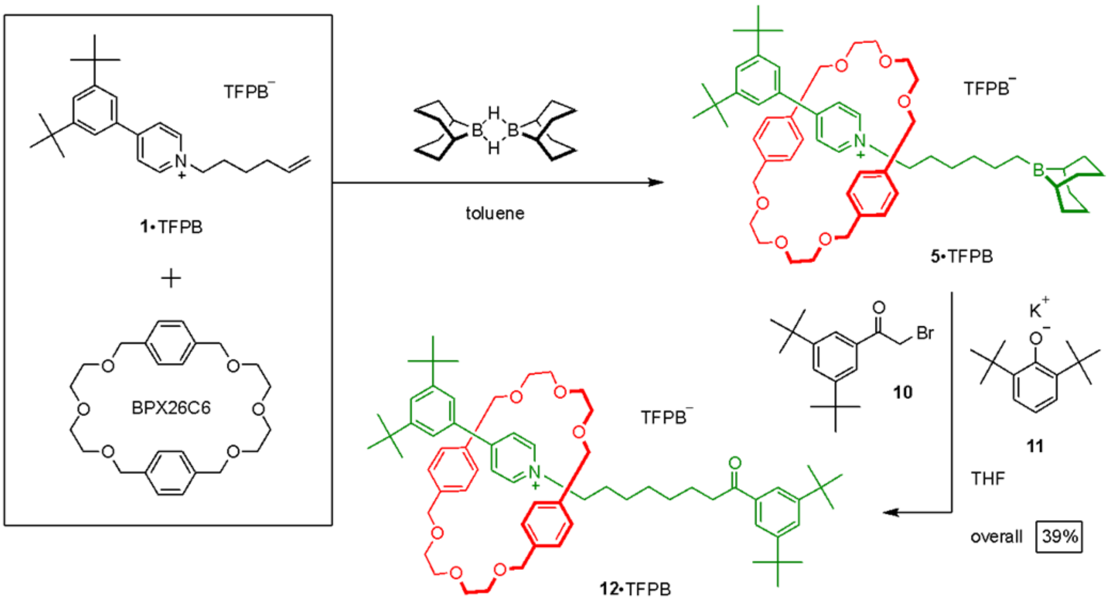

Pyridinium rotaxane 12·TFPB: In a glovebox, the pre-dried pyridinium salt **1**·TFPB (72.8 mg, 60.0 µmol) and BPX26C6 (25.0 mg, 60.0 µmol) were mixed in anhydrous toluene (0.15 mL). The mixture was stirred at 60 °C for 10 min and turned homogeneous. 9-BBN dimer (8.05 mg, 66.0 µmol as monomer) was added and this mixture was stirred at 60 °C for 18 h to afford the crude 9-BBN-stoppered rotaxane **5**·TFPB. After cooling to 0 °C, the mixture was diluted with anhydrous THF (0.3 mL) and treated with a THF (0.1 mL) solution suspension of phenolate salt **11**[4] (16.1 mg, 65.9 µmol). After stirring at 0 °C for 5 min, the mixture was treated with the α-bromoketone **10**[5] (20.5 mg, 65.9 µmol in 0.2 mL of THF) and then the resulting red mixture was stirred at 0 °C for 3 h. After warming to room temperature, the mixture was moved to a fume hood and EtOH (0.3 mL) was added to quench the reaction. The mixture was stirred at room temperature for 30 min and then concentrated. The residue was purified chromatographically [first: SiO_2; CH_2Cl_2/hexane (gradient from 5:5 to 10:0); second: RP-18 gel; MeOH; third: DIOL gel; EtOAc/hexane (2:8) then CH_2Cl_2/hexane (5:5)] to afford the pyridinium rotaxane **12**·TFPB as a sticky colorless liquid (43.4 mg, 39%). ^{1}H NMR (800 MHz, $CDCl_3$): δ = 7.82 (d, J = 1.6 Hz, 2H), 7.76 (d, J = 7.2 Hz, 2H), 7.73 (t, J = 1.6 Hz, 1H), 7.72–7.68 (m, 8H), 7.67 (d, J = 7.2 Hz, 2H), 7.65 (t, J = 1.6 Hz, 1H), 7.58 (d, J = 1.6 Hz, 2H), 7.51 (s, 4H), 6.71 (s, 8H), 4.22 (d, J = 9.6 Hz, 4H), 4.06 (d, J = 9.6 Hz, 4H), 3.74–3.61 (m, 16H), 3.47–3.42 (m, 2H), 3.04 (t, J = 7.2 Hz, 2H), 1.84–1.77 (m, 2H), 1.50–1.39 (m, 24H), 1.36–1.28 (m, 20H); ^{13}C NMR (200 MHz, $CDCl_3$): δ = 200.9, 161.7 (q, $^1J_{CB}$ = 49.6 Hz), 154.5, 152.7, 151.4, 143.9, 136.7, 136.6, 134.8, 133.3, 129.2–128.6 (m), 128.4, 127.4, 126.5, 124.7, 124.6 (q, $^1J_{CF}$ = 270.8 Hz), 122.2, 121.9, 117.5–117.3 (m), 73.6, 71.1, 70.2, 59.5, 38.3, 35.2, 35.0, 31.4, 31.3, 29.7, 29.2, 29.1, 26.4, 24.0; HR-MS (ESI): calcd for [**12**]$^+$, $C_{65}H_{92}NO_7^+$: m/z 998.6868; found 998.6875.

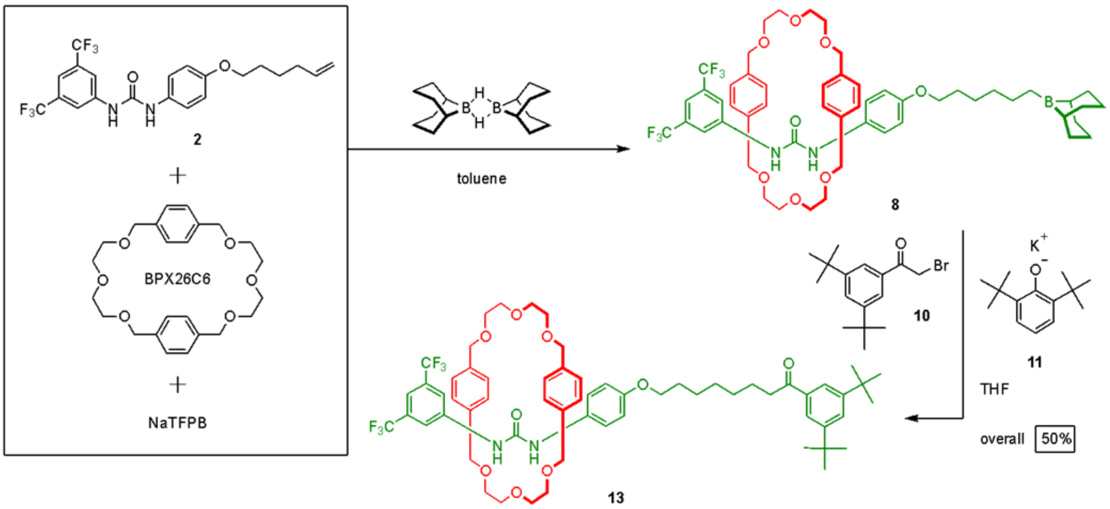

Urea rotaxane 13: In a glovebox, the pre-dried urea **2** (26.8 mg, 60.0 μmol), BPX26C6 (25.0 mg, 60.0 μmol), and NaTFPB (pre-ground as a fine powder; 53.2 mg, 60.0 μmol) were mixed in anhydrous toluene (0.15 mL). The mixture was stirred at 60 °C for 18 h at which point it had turned homogeneous. 9-BBN dimer (8.05 mg, 66.0 μmol as monomer) was added and this mixture was stirred at 60 °C for another 18 h to afford the crude 9-BBN-stoppered rotaxane **8**. After cooling to 0 °C, the mixture was diluted with anhydrous THF (0.3 mL) and treated with a THF (0.1 mL) solution suspension of phenolate salt **11** (16.1 mg, 65.9 μmol). After stirring at 0 °C for 5 min, the α-bromoketone **10** (20.5 mg, 65.9 μmol dissolved in 0.2 mL of THF) was added and the resulting red mixture was stirred at 0 °C for 3 h. After warming to room temperature, the mixture was moved to a fume hood and EtOH (0.3 mL) was added to quench the reaction. The mixture was stirred at room temperature for 30 min and then concentrated. The residue was purified chromatographically [first: SiO_2; EtOAc/hexane (gradient from 1:9 to 3:7); second: RP-18 gel; MeOH] to afford the urea rotaxane **13** as a sticky colorless liquid (32.8 mg, 50%). ^{1}H NMR (500 MHz, CD_2Cl_2): δ = 7.89–7.68 (m, 4H), 7.66 (t, J = 2.0 Hz, 1H), 7.44 (s, 1H), 7.23 (br, 1H), 6.91 (s, 8H), 6.85 (s, 1H), 6.71 (br, 4H), 4.33 (d, J = 11.0 Hz, 4H), 4.24 (d, J = 11.0 Hz, 4H), 3.95 (t, J = 6.5 Hz, 2H), 3.70–3.47 (m, 16H), 3.00 (t, J = 7.0 Hz, 2H), 1.84–1.71 (m, 4H), 1.56–1.43 (m, 6H), 1.37 (s, 18H); ^{13}C NMR (100 MHz, $CDCl_3$): δ = 201.2, 154.7, 151.1, 150.6, 141.7, 136.8, 131.1 (q, $^{2}J_{CF}$ = 32.7 Hz), 128.3, 127.1, 123.6 (q, $^{1}J_{CF}$ = 271.1 Hz), 122.2, 121.6 (br), 118.7 (br), 114.3, 114.0 (br), 73.3, 70.7, 68.8, 68.3, 38.6, 35.0, 31.4, 29.4, 29.4, 29.4, 26.0, 24.4 (two signals were missing, possibly due to signal overlap); HR-MS (ESI): calcd for [**13** + Na]$^+$, $C_{61}H_{76}F_6N_2NaO_9^+$: m/z 1117.5347; found 1117.5322.

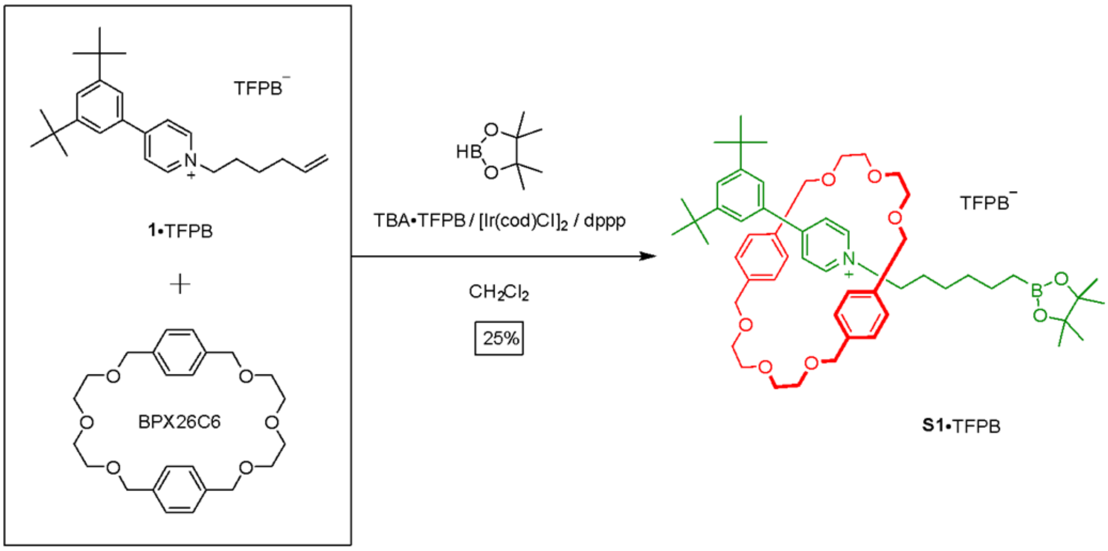

Pyridinium rotaxane S1·TFPB: In a glovebox, a solution of pinacolborane (18.4 mg, 0.144 mmol) in CH_2Cl_2 (0.2 mL) was added to a mixture of the pyridinium salt **1**·TFPB (146 mg, 0.120 mmol), TBA·TFPB[6] (133 mg, 0.120 mmol), and BPX26C6 (50.0 mg, 0.120 mmol) in CH_2Cl_2 (0.2 mL). The resulting mixture was treated sequentially with 1,3-bis(diphenylphosphino)propane (dppp, 0.74 mg, 1.79 µmol) and $[Ir(cod)Cl]_2$ (2.42 mg, 3.60 µmol) and then stirred at room temperature for 18 h. The mixture was moved to a fume hood and partitioned between CH_2Cl_2 (20 mL) and H_2O (20 mL). The aqueous phase was extracted with CH_2Cl_2 (3 × 10 mL) and the combined organic phases were dried ($MgSO_4$) and concentrated. The residue was purified chromatographically [SiO_2; CH_2Cl_2/hexane (gradient from 5:5 to 8:2)] to afford the pyridinium rotaxane **S1**·TFPB as a sticky colorless liquid (52.3 mg, 25%). ^{1}H NMR (400 MHz, CD_2Cl_2): δ = 7.85 (d, J = 5.6 Hz, 2H), 7.83–7.72 (m, 11H), 7.68 (s, 2H), 7.61 (s, 4H), 6.80 (s, 8H), 4.28 (d, J = 9.6 Hz, 4H), 4.13 (d, J = 9.6 Hz, 4H), 3.81–3.65 (m, 16H), 3.60–3.49 (m, 2H), 1.61–1.34 (m, 26H), 1.27 (s, 12H), 0.87 (t, J = 7.2 Hz, 2H); ^{13}C NMR (100 MHz, CD_2Cl_2): δ = 162.3 (q, $^1J_{CB}$ = 49.7 Hz), 154.9, 153.1, 144.5, 137.4, 135.3, 134.0, 130.0–128.9 (m), 128.9, 126.9, 125.3, 125.1 (q, $^1J_{CF}$ = 270.7 Hz), 122.5, 118.2–117.8 (m), 83.4, 73.9, 71.6, 70.8, 60.2, 35.6, 32.2, 31.6, 30.2, 26.8, 25.1, 24.3 (one signal was missing, possibly due to signal overlap or the inability to observe the carbon attached to boron, likely due to quadrupolar relaxation); HR-MS (ESI): calcd for [**S1**]$^+$, $C_{55}H_{81}BNO_8^+$: m/z 894.6050; found 894.6050.

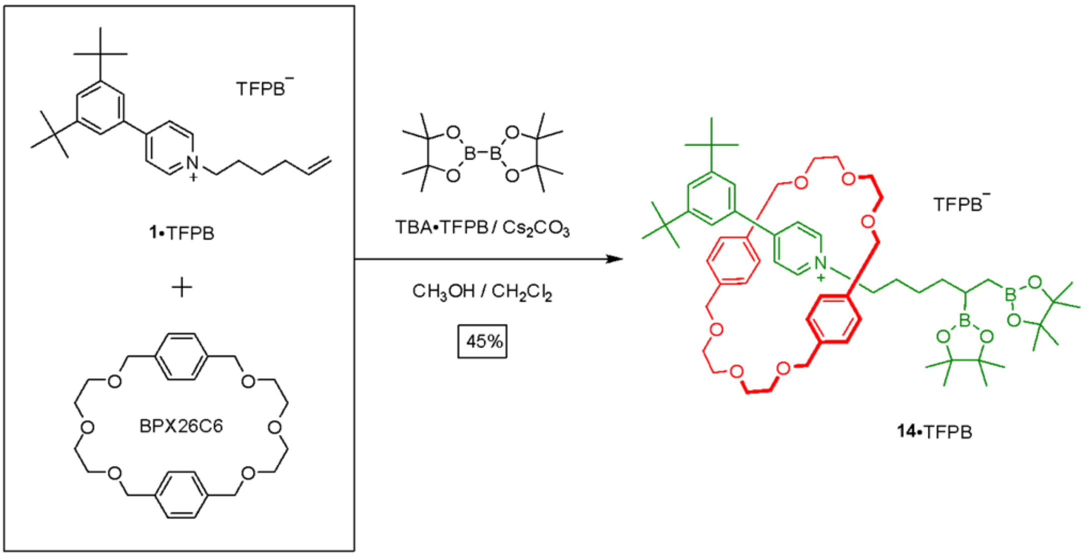

Pyridinium rotaxane 14·TFPB: The pyridinium salt **1**·TFPB (364 mg, 0.300 mmol), BPX26C6 (125 mg, 0.300 mmol), TBA·TFPB (332 mg, 0.300 mmol), bis(pinacolato)diboron (152 mg, 0.599 mmol), and Cs_2CO_3 (195 mg, 0.599 mmol) were mixed in a pressure tube with anhydrous CH_2Cl_2 (1.2 mL) and anhydrous CH_3OH (48.7 μL, 1.20 mmol). The resulting mixture was stirred at 40 °C for 18 h and then partitioned between CH_2Cl_2 (20 mL) and H_2O (20 mL). The aqueous phase was extracted with CH_2Cl_2 (3 × 10 mL) and then the combined organic phases were dried ($MgSO_4$) and concentrated. The residue was purified chromatographically [first: SiO_2; CH_2Cl_2/hexane (gradient from 5:5 to 10:0) and then acetone/CH_2Cl_2 (2:98); second: DIOL gel; CH_2Cl_2/hexane (1:1)] to afford the pyridinium rotaxane **14**·TFPB as a pale-orange solid (252 mg, 45%). M.p. = 42–43 °C; ^{1}H NMR (400 MHz, CD_2Cl_2): δ = 7.83 (d, J = 6.8 Hz, 2H), 7.80–7.70 (m, 11H), 7.65 (d, J = 2.0 Hz, 2H), 7.58 (s, 4H), 6.79 (s, 8H), 4.27 (d, J = 9.6 Hz, 4H), 4.12 (d, J = 9.6 Hz, 4H), 3.82–3.64 (m, 16H), 3.58–3.48 (m, 2H), 1.64–1.36 (m, 24H), 1.29 (s, 12H), 1.26 (s, 12H), 1.22–1.13 (m, 1H), 0.96–0.81 (m, 2H); ^{13}C NMR (100 MHz, CD_2Cl_2): δ = 162.2 (q, $^{1}J_{CB}$ = 49.5 Hz), 154.9, 153.0, 144.4, 137.3, 135.2, 134.0, 129.9–128.8 (m), 128.8, 126.8, 125.2, 125.1 (q, $^{1}J_{CF}$ = 270.7 Hz), 122.5, 118.2–117.7 (m), 83.5, 83.4, 73.9, 71.6, 70.7, 60.0, 35.6, 33.7, 31.6, 30.3, 26.1, 25.2, 25.1, 25.0 (three signals were missing, possibly due to signal overlap and the inability to observe the carbon attached to boron, likely due to quadrupolar relaxation); HR-MS (ESI): calcd for $[\mathbf{14}]^{+}$, $C_{61}H_{92}B_2NO_{10}^{+}$: m/z 1020.6902; found 1020.6937.

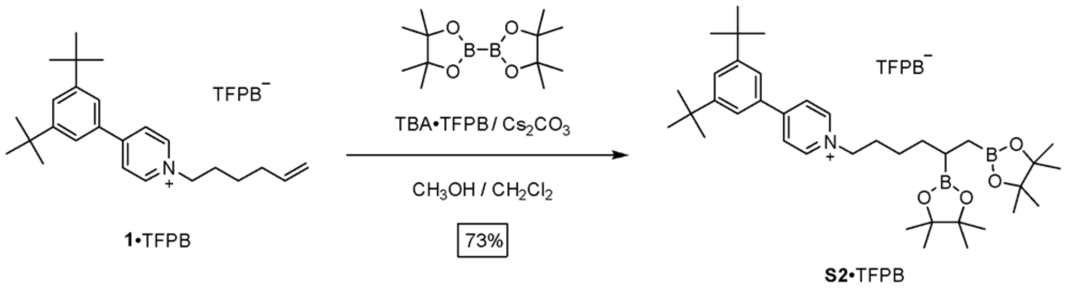

Dumbbell-shaped pyridinium salt S2·TFPB: The pyridinium salt **1**·TFPB (364 mg, 0.300 mmol), TBA·TFPB (332 mg, 0.300 mmol), bis(pinacolato)diboron (152 mg, 0.599 mmol), and Cs_2CO_3 (195 mg, 0.599 mmol) were mixed in a pressure tube with anhydrous CH_2Cl_2 (1.2 mL) and anhydrous CH_3OH (48.7 μL, 1.20 mmol). The resulting mixture was stirred at 40 °C for 18 h and then partitioned between CH_2Cl_2 (20 mL) and H_2O (20 mL). The aqueous phase was extracted with CH_2Cl_2 (3 × 10 mL) and then the combined organic phases were dried ($MgSO_4$) and concentrated. The residue was purified chromatographically [SiO_2; CH_2Cl_2/hexane (gradient from 5:5 to 10:0) and then acetone/CH_2Cl_2 (1:9)] to afford the dumbbell-shaped pyridinium salt **S2**·TFPB as a pale-orange solid (320 mg, 73%). M.p. = 51–52 °C; ^{1}H NMR (400 MHz, CD_2Cl_2): δ = 8.46 (d, J = 6.8 Hz, 2H), 8.22 (d, J = 6.8 Hz, 2H), 7.82 (t, J = 1.6 Hz, 1H), 7.78 (s, 8H), 7.62 (d, J =1.6 Hz, 2H), 7.60 (s, 4H), 4.49 (t, J = 7.2 Hz, 2H), 2.13–2.01 (m, 2H), 1.65–1.37 (m, 22H), 1.26–1.19 (m, 24H), 1.18–1.06 (m, 1H), 0.92–0.74 (m, 2H); ^{13}C NMR (100 MHz, CD_2Cl_2): δ = 162.2 (q, $^1J_{CB}$ = 49.5 Hz), 160.1, 153.9, 143.3, 135.3, 133.0, 129.9–128.8 (m), 128.5, 126.2, 125.1 (q, $^1J_{CF}$ = 270.8 Hz), 122.7, 118.3–117.6 (m), 83.5, 83.4, 62.2, 35.5, 32.9, 31.7, 31.3, 25.4, 25.1, 25.0, 25.0, 24.9 (two signals were missing, possibly due to the inability to observe the carbon attached to boron, likely due to quadrupolar relaxation); HR-MS (ESI): calcd for [**S2**]$^+$, $C_{37}H_{60}B_2NO_4^+$: m/z 604.4703; found 604.4714.

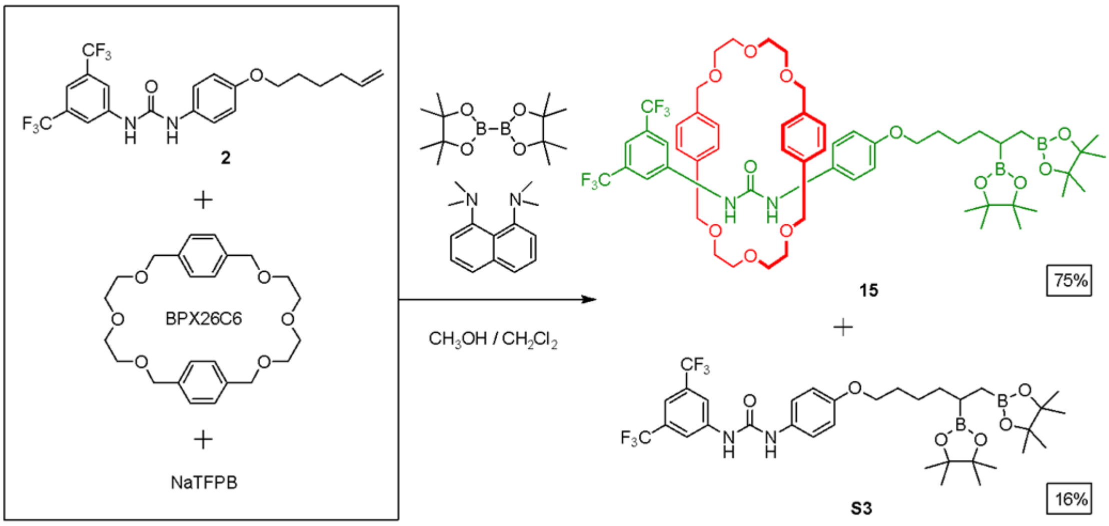

Urea rotaxane 15 and dumbbell-shaped urea S3: The urea **2** (134 mg, 0.300 mmol), BPX26C6 (375 mg, 0.900 mmol), NaTFPB (798 mg, 0.901 mmol), bis(pinacolato)diboron (152 mg, 0.599 mmol), and 1,8-bis(dimethylamino)naphthalene (Proton-sponge®, 32.1 mg, 0.150 mmol) were mixed in a pressure tube with anhydrous CH_2Cl_2 (1.2 mL) and anhydrous CH_3OH (36.5 μL, 0.900 mmol). The resulting mixture was stirred at 40 °C for 18 h, cooled to room temperature, and concentrated. The residue was purified chromatographically [SiO_2; EtOAc/hexane (gradient from 2:8 to 4:6)] to afford the urea rotaxane **15** as a sticky colorless liquid (250 mg, 75%) and the dumbbell-shaped urea **S3** as a white solid (33.5 mg, 16%).

Data for the urea rotaxane 15: ^{1}H NMR (800 MHz, CD_2Cl_2): δ = 7.81 (br, 2H), 7.45 (s, 1H), 7.24 (br, 1H), 6.93 (s, 8H), 6.86 (s, 1H), 6.78–6.65 (m, 4H), 4.34 (d, *J* = 11.2 Hz, 4H), 4.25 (d, *J* = 11.2 Hz, 4H), 3.94 (t, *J* = 6.4 Hz, 2H), 3.79–3.41 (m, 16H), 1.82–1.75 (m, 2H), 1.59–1.47 (m, 3H), 1.46–1.38 (m, 1H), 1.31–1.18 (m, 24H), 1.15–1.10 (m, 1H), 0.89–0.77 (m, 2H); ^{13}C NMR (100 MHz, $CDCl_3$): δ = 154.3, 150.2, 141.5, 136.4, 130.6 (q, $^2J_{CF}$ = 32.5 Hz), 127.9, 123.3 (q, $^1J_{CF}$ = 271.0 Hz), 121.2 (br), 118.3 (br), 113.8, 113.3 (br), 82.3, 72.8, 70.2, 68.3, 67.7, 33.1, 29.1, 24.8, 24.4, 24.4, 24.3, 18.0 (br), 12.3 (br) (Three signals were missing, possibly due to signal overlap); HR-MS (ESI): calcd for [**15** + Na]$^+$, $C_{57}H_{76}B_2F_6N_2NaO_{12}{}^+$: *m*/*z* 1139.5381; found 1139.5370.

Data for the dumbbell-shaped urea S3: M.p. = 47–48 °C; ^{1}H NMR (400 MHz, $CDCl_3$): δ = 7.87 (br, 1H), 7.68 (s, 2H), 7.45–7.26 (m, 2H), 7.07 (d, *J* = 8.4 Hz, 2H), 6.72 (d, *J* = 8.4 Hz, 2H), 3.77 (t, *J* = 6.4 Hz, 2H), 1.74–1.61 (m, 2H), 1.54–1.31 (m, 4H), 1.24–1.06 (m, 25H), 0.91–0.74 (m, 2H); ^{13}C NMR (100 MHz, $CDCl_3$): δ = 156.7, 154.5, 139.9, 132.0 (q, $^2J_{CF}$ = 33.1 Hz), 129.0, 123.8, 122.9 (q, $^1J_{CF}$ = 271.1 Hz), 119.0, 116.3–115.8 (m), 114.8, 82.8, 82.8, 68.0, 33.4, 29.3, 25.1, 24.8, 24.7, 24.7, 24.6, 18.3 (br), 12.6 (br); HR-MS (ESI): calcd for [**S3** + Na]$^+$, $C_{33}H_{44}B_2F_6N_2NaO_6{}^+$: *m*/*z* 723.3182; found 723.3161.

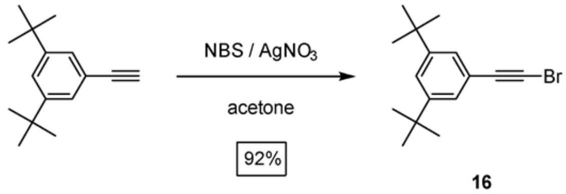

16

Bromoalkyne 16: *N*-Bromosuccinimide (270 mg, 1.52 mmol) and $AgNO_3$ (11.7 mg, 68.9 μmol) were added sequentially to a solution of 1,3-di-*tert*-butyl-5-

ethynylbenzene[7] (296 mg, 1.38 mmol) in acetone (14 mL). The mixture was stirred in the dark at room temperature for 2 h and then concentrated. The residue was purified chromatographically (SiO_2; hexane) to afford the bromoalkyne **16** (371 mg, 92%) as a white solid. M.p. = 90–91 °C; ^{1}H NMR (400 MHz, $CDCl_3$): δ = 7.38 (s, 1H), 7.29 (s, 2H), 1.29 (s, 18H); ^{13}C NMR (100 MHz, $CDCl_3$): δ = 150.8, 126.2, 123.1, 121.7, 81.1, 48.1, 34.8, 31.3; HR-MS (ESI): calcd for [**16** + H]$^+$, $C_{16}H_{22}Br^+$: *m*/*z* 293.0899; found 293.0893.

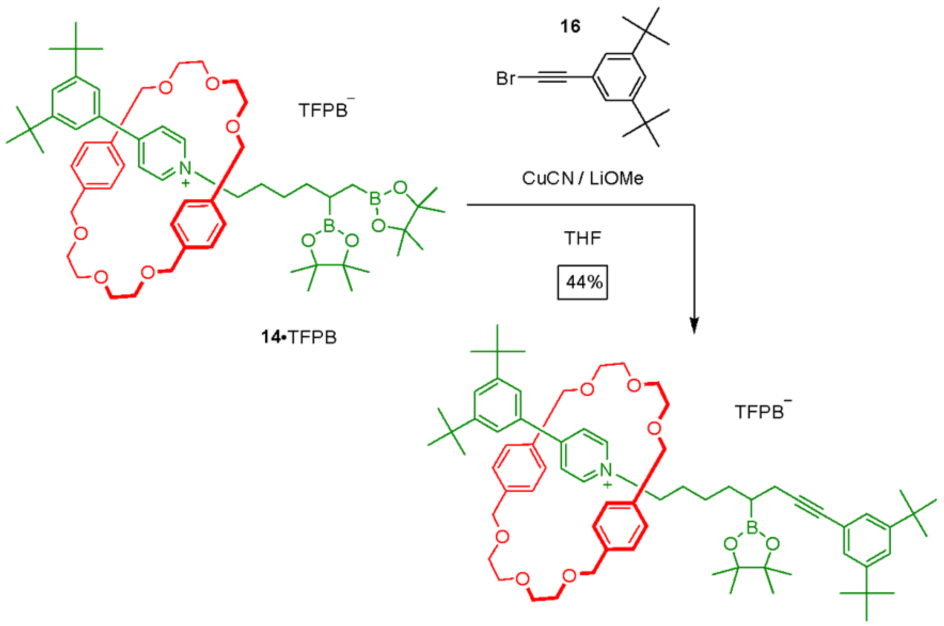

Pyridinium rotaxane 17·TFPB: Anhydrous THF (450 μL) was added to a solid mixture of the pyridinium rotaxane **14**·TFPB (169 mg, 89.7 μmol), the bromoalkyne **16** (31.7 mg, 108 μmol), CuCN (2.42 mg, 27.0 μmol), and LiOMe (10.3 mg, 271 μmol) and then the resulting mixture was stirred at 60 °C for 16 h. After cooling to room temperature, the mixture was diluted with CH_2Cl_2 (10 mL) and silica gel (1 g) was added. After concentrating, the residue was purified chromatographically [SiO_2; CH_2Cl_2/hexane (gradient from 5:5 to 8:2)] to afford the pyridinium rotaxane **17**·TFPB as a white solid (77.9 mg, 44 %; because of purification challenges, the yield was determined based on the pure fraction obtained from column chromatography.). M.p. = 56–57 °C; ^{1}H NMR (400 MHz, $CDCl_3$): δ = 7.81–7.67 (m, 13H), 7.59 (d, *J* = 1.6 Hz, 2H), 7.54 (s, 4H), 7.34 (t, *J* = 1.6 Hz, 1H), 7.25 (d, *J* = 1.6 Hz, 2H), 6.73 (s, 8H), 4.20 (d, *J* = 10.0 Hz, 4H), 4.12–4.01 (m, 4H), 3.78–3.57 (m, 16H), 3.53–3.41 (m, 2H), 2.68–2.55 (m, 2H), 1.76–1.60 (m, 2H), 1.54–1.36 (m, 23H), 1.34–1.29 (m, 12H), 1.27 (s, 18H); ^{13}C NMR (100 MHz, $CDCl_3$): δ = 161.7 (q, $^1J_{CB}$ = 49.6 Hz), 154.7, 152.8, 150.8, 143.9, 136.7, 134.8, 133.3, 129.5–128.4 (m), 128.4, 126.6, 125.7, 124.6, 124.6 (q, $^1J_{CF}$ = 271.0 Hz), 122.9, 122.2, 121.9, 117.7–117.2 (m), 88.2, 83.6, 82.3, 73.5, 71.1, 70.2, 59.4, 35.2, 34.7, 31.4, 31.3, 29.7, 25.6, 24.8, 24.8, 23.4 (br), 20.7 (one signal was missing, possibly due to signal overlap); HR-MS (ESI): calcd for [**17**]$^+$,

$C_{71}H_{101}BNO_8^+$: *m*/*z* 1106.7615; found 1106.7640.

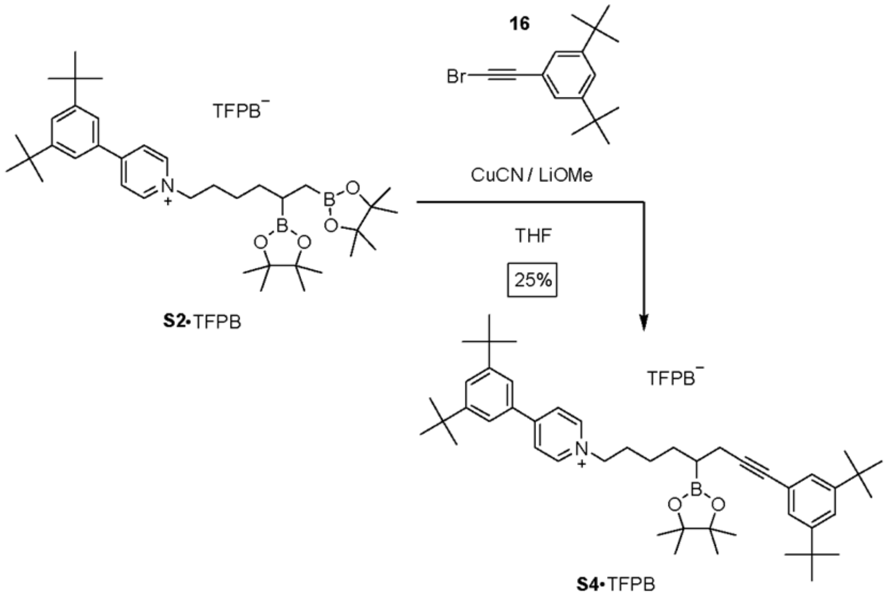

Dumbbell-shaped pyridinium salt S4·TFPB: A mixture of the dumbbell-shaped pyridinium salt **S2**·TFPB (132 mg, 89.9 µmol), the bromoalkyne **16** (31.7 mg, 108 µmol), CuCN (2.42 mg, 27.0 µmol), and LiOMe (10.3 mg, 271 µmol) in anhydrous THF (450 µL) was stirred at 60 °C for 16 h. After cooling to room temperature, the mixture was diluted with CH_2Cl_2 (10 mL), treated with silica gel (1 g), and concentrated. The residue was purified chromatographically [SiO_2; CH_2Cl_2/hexane (gradient from 5:5 to 10:0) and then acetone/CH_2Cl_2 (2:98)] to afford the dumbbell-shaped pyridinium salt **S4**·TFPB as a sticky pale-brown liquid. (35.5 mg, 25%). ^{1}H NMR (400 MHz, $CDCl_3$): δ = 8.12 (d, *J* = 7.2 Hz, 2H), 7.99 (d, *J* = 7.2 Hz, 2H), 7.75 (t, *J* = 1.6 Hz, 1H), 7.68 (s, 8H), 7.52–7.45 (m, 6H), 7.33 (t, *J* = 2.0 Hz, 1H), 7.21 (d, *J* = 2.0 Hz, 2H), 4.25 (t, *J* = 7.2 Hz, 2H), 2.59–2.41 (m, 2H), 2.02–1.89 (m, 2H), 1.73–1.57 (m, 2H), 1.46–1.31 (m, 20H), 1.29–1.23 (m, 19H), 1.21 (s, 12H); ^{13}C NMR (100 MHz, $CDCl_3$): δ = 161.7 (q, $^1J_{CB}$ = 49.6 Hz), 160.0, 153.6, 150.9, 142.4, 134.8, 132.2, 129.6–128.2 (m), 125.6, 125.6, 124.5 (q, $^1J_{CF}$ = 270.8 Hz), 122.8, 122.2, 122.1, 117.8–117.3 (m), 88.2, 83.6, 82.2, 61.5, 35.2, 34.7, 31.3, 31.1, 28.9, 24.7, 24.7, 24.7, 23.0 (br), 20.5 (two signals were missing, possibly due to signal overlap); HR-MS (ESI): calcd for [**S4**]$^+$, $C_{47}H_{69}O_2NB^+$: *m*/*z* 690.5416; found 690.5439.

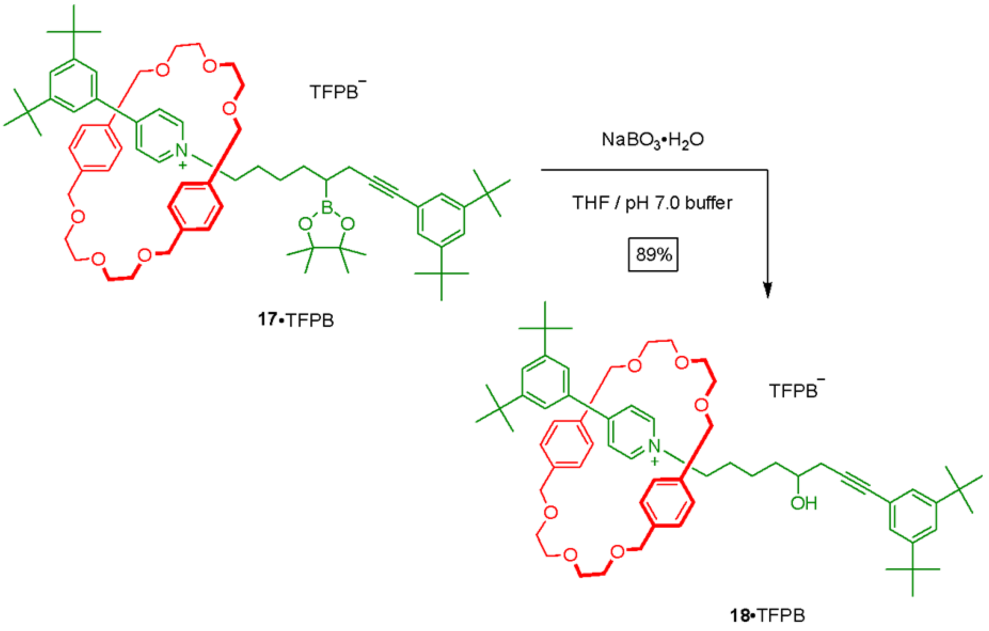

Pyridinium rotaxane 18·TFPB: $NaBO_3 \cdot H_2O$ (8.31 mg, 83.2 μmol) was added to a mixture of the pyridinium rotaxane **17**·TFPB (82.0 mg, 41.6 μmol) in THF (420 μL) and an aqueous buffer solution (pH 7.0; 0.5 M NaOH-$KH_2PO_{4(aq.)}$, 210 μL). After stirring at room temperature for 3 h, the mixture was partitioned between CH_2Cl_2 (10 mL) and H_2O (10 mL). The aqueous phase was extracted with CH_2Cl_2 (3 × 10 mL); the combined organic phases were dried ($MgSO_4$) and concentrated. The residue was purified chromatographically [SiO_2; CH_2Cl_2/hexane (gradient from 7:3 to 10:0) and then acetone/CH_2Cl_2 (5:95)] to afford the pyridinium rotaxane **18**·TFPB as a white solid (69.1 mg, 89%). M.p. = 50–51 °C; ^{1}H NMR (400 MHz, $CDCl_3$): δ = 7.81 (d, J = 6.8 Hz, 2H), 7.77–7.66 (m, 11H), 7.60 (d, J = 2.0 Hz, 2H), 7.54 (s, 4H), 7.39 (t, J = 1.6 Hz, 1H), 7.29 (d, J = 1.6 Hz, 2H), 6.74 (s, 8H), 4.29–4.15 (m, 4H), 4.08 (d, J = 9.6 Hz, 4H), 3.97–3.84 (m, 1H), 3.79–3.55 (m, 16H), 3.55–3.42 (m, 2H), 2.78–2.58 (m, 2H), 2.31 (d, J = 4.8 Hz, 1H), 1.77–1.62 (m, 2H), 1.58–1.38 (m, 22H), 1.29 (s, 18H); ^{13}C NMR (100 MHz, $CDCl_3$): δ = 161.7 (q, $^1J_{CB}$ = 49.6 Hz), 154.7, 152.8, 151.0, 144.0, 136.8, 134.8, 133.3, 129.5–128.5 (m), 128.5, 126.6, 125.8, 124.6, 124.6 (q, $^1J_{CF}$ = 270.9 Hz), 122.7, 122.0, 121.8, 117.9–117.0 (m), 84.6, 84.1, 73.6, 71.1, 70.2, 69.7, 59.3, 35.3, 35.2, 34.8, 31.4, 31.2, 29.3, 28.9, 22.8; HR-MS (ESI): calcd for [**18**]$^+$, $C_{65}H_{90}NO_7^+$: m/z 996.6712; found 996.6706.

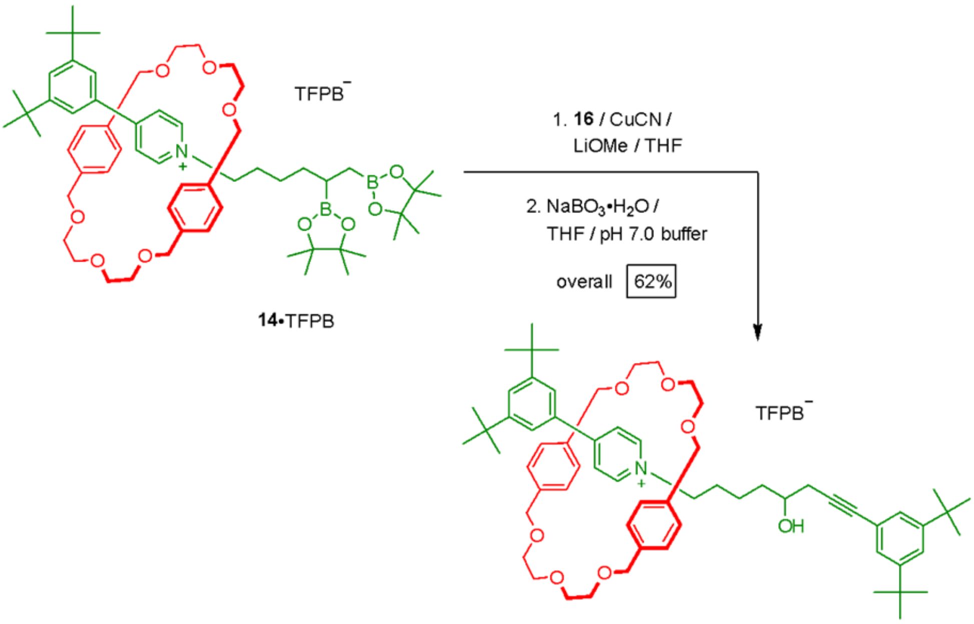

The pyridinium rotaxane ***18***·*TFPB can be synthesized more efficiently by oxidizing the crude rotaxane* ***17***·*TFPB.*

Pyridinium rotaxane 18·TFPB: A mixture of the pyridinium rotaxane **14**·TFPB (56.5 mg, 30.0 µmol), the bromoalkyne **16** (10.6 mg, 36.1 µmol), CuCN (0.81 mg, 9.04 µmol), and LiOMe (3.42 mg, 90.1 µmol) in anhydrous THF (150 µL) was stirred at 60 °C for 16 h. After cooling to room temperature, the mixture was diluted with CH_2Cl_2 (10 mL), treated with silica gel (1 g), and concentrated. The residue was purified chromatographically [SiO_2; CH_2Cl_2/hexane (gradient from 5:5 to 10:0)] to afford the crude rotaxane **17**·TFPB, which was used directly in the next step. The crude pyridinium rotaxane **17**·TFPB was dissolved in THF (300 µL) and an aqueous buffer solution (pH 7.0; 0.5 M NaOH-$KH_2PO_{4(aq.)}$, 150 µL) and then this mixture was treated with $NaBO_3$·H_2O (5.99 mg, 60.0 µmol). The resulting mixture was stirred at room temperature for 3 h and then partitioned between CH_2Cl_2 (10 mL) and H_2O (10 mL). The aqueous phase was extracted with CH_2Cl_2 (3 × 10 mL); the combined organic phases were dried ($MgSO_4$) and concentrated. The residue was purified chromatographically [SiO_2; CH_2Cl_2/hexane (gradient from 7:3 to 10:0) and then acetone/CH_2Cl_2 (5:95)] to afford the pyridinium rotaxane **18**·TFPB as a white solid (34.5 mg, 62%).

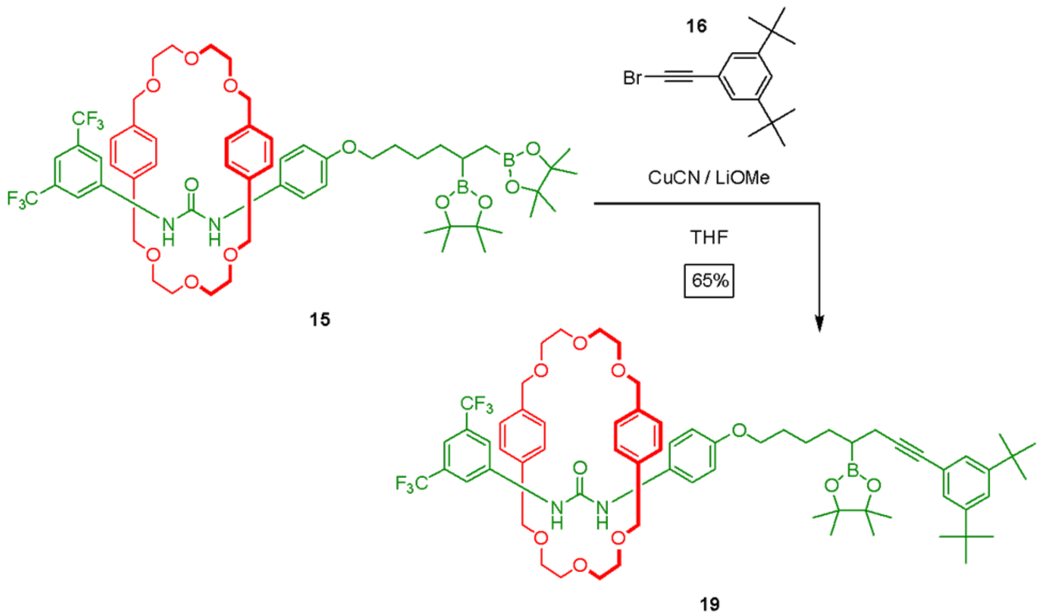

Urea rotaxane 19: A mixture of the urea rotaxane **15** (101 mg, 90.4 µmol), the bromoalkyne **16** (31.7 mg, 108 µmol), CuCN (16.1 mg, 180 µmol), and LiOMe (10.3 mg, 271 µmol) in anhydrous THF (900 µL) was stirred at 60 °C for 4 h. After cooling to room temperature, the mixture was diluted with CH_2Cl_2 (10 mL), treated with silica gel (1 g), and concentrated. The residue was purified chromatographically [SiO_2; acetone/hexane (15:75)] to afford the urea rotaxane **19** as a sticky colorless liquid (69.9 mg, 65%). ^{1}H NMR (500 MHz, $CDCl_3$): δ = 7.72 (br, 2H), 7.39 (s, 1H), 7.30 (t, J = 1.5 Hz, 1H), 7.22 (d, J = 1.5 Hz, 2H), 7.04 (br, 1H), 6.96–6.79 (m, 9H), 6.77–6.55 (m, 4H), 4.32 (d, J = 11.0 Hz, 4H), 4.22 (d, J = 11.0 Hz, 4H), 3.93 (t, J = 6.5 Hz, 2H), 3.71–3.39 (m, 16H), 2.51 (d, J = 7.5 Hz, 2H), 1.87–1.77 (m, 2H), 1.70–1.48 (m, 4H), 1.41–1.34 (m, 1H), 1.31–1.24 (m, 30H); ^{13}C NMR (125 MHz, $CDCl_3$): δ = 154.7, 150.6, 150.5, 141.7, 136.8, 131.1 (q, $^2J_{CF}$ = 32.5 Hz), 128.4, 125.7, 123.6 (q, $^1J_{CF}$ = 271.0 Hz), 123.1, 121.8, 118.8 (br), 114.2, 114.0 (br), 88.7, 83.3, 81.8, 73.3, 70.7, 68.8, 68.2, 34.7, 31.3, 30.4, 29.6, 25.3, 24.9, 24.8, 23.8 (br), 20.8 (two signals were missing, possibly due to signal overlap); HR-MS (ESI): calcd for [**19** + H]$^+$, $C_{67}H_{86}BF_6N_2O_{10}^+$: m/z 1203.6274; found 1203.6270.

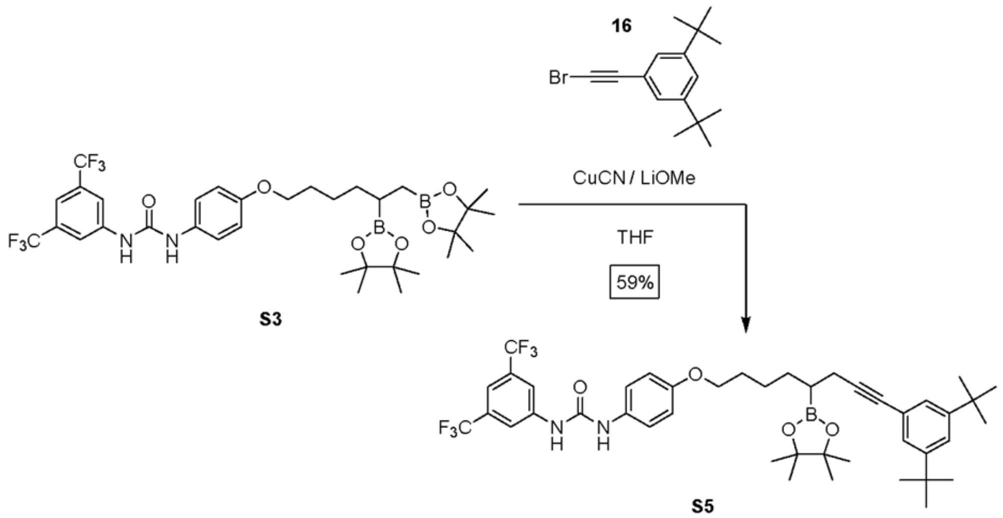

Dumbbell-shaped urea S5: A mixture of the semidumbbell-shaped urea **S3** (63.0 mg, 90.0 µmol), the bromoalkyne **16** (31.7 mg, 108 µmol), CuCN (16.1 mg, 180 µmol), and LiOMe (10.3 mg, 271 µmol) in anhydrous THF (900 µL) was stirred at 60 °C for 4 h. After cooling to room temperature, the mixture was diluted with CH_2Cl_2 (10 mL), treated with silica gel (1 g), and concentrated. The residue was purified chromatographically [first: SiO_2; acetone/hexane (15:75); second: DIOL gel; CH_2Cl_2/hexane (gradient from 5:5 to 7:3)] to afford the dumbbell-shaped urea **S5** as a white solid (41.5 mg, 59%). M.p. = 68–69 °C; ^{1}H NMR (400 MHz, $CDCl_3$): δ = 7.76 (s, 2H), 7.45 (s, 1H), 7.41–7.26 (m, 2H), 7.21 (d, J = 1.6 Hz, 2H), 7.14 (d, J = 8.8 Hz, 2H), 6.91 (br, 1H), 6.81 (d, J = 8.8 Hz, 2H), 3.88 (t, J = 6.4 Hz, 2H), 2.49 (d, J = 7.2 Hz, 2H), 1.86–1.72 (m, 2H), 1.65–1.44 (m, 4H), 1.40–1.13 (m, 31H); ^{13}C NMR (100 MHz, $CDCl_3$): δ = 157.3, 153.8, 150.5, 140.0, 132.2 (q, $^2J_{CF}$ = 33.0 Hz), 128.9, 125.7, 125.0 (br), 123.0 123.0 (q, $^1J_{CF}$ = 270.0 Hz), 121.8, 119.0, 116.5–116.0 (m), 115.3, 88.7, 83.3, 81.8, 68.1, 34.7, 31.3, 30.3, 29.3, 25.2, 24.8, 24.8, 23.8 (br), 20.8; HR-MS (ESI): calcd for [**S5** + H]$^+$, $C_{43}H_{54}O_4N_2BF_6^+$: m/z 787.4075; found 787.4089.

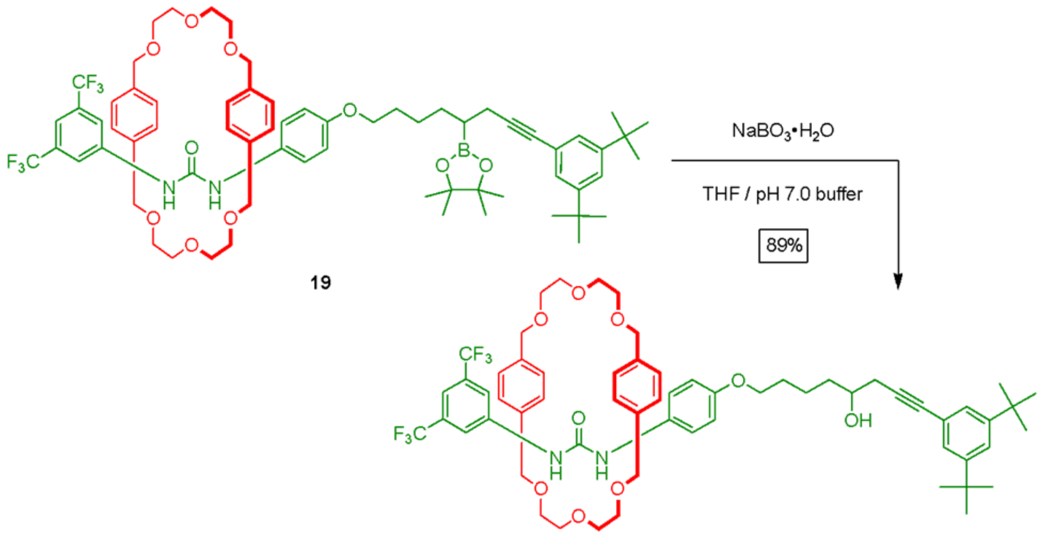

Urea rotaxane 20: $NaBO_3 \cdot H_2O$ (11.4 mg, 114 μmol) was added to a mixture of the urea rotaxane **19** (68.8 mg, 57.2 μmol) in THF (570 μL) and an aqueous buffer solution (pH 7.0, 0.5 M NaOH-$KH_2PO_{4(aq.)}$, 285 μL). The resulting mixture was stirred at room temperature for 3 h and then partitioned between CH_2Cl_2 (10 mL) and H_2O (10 mL). The aqueous phase was extracted with CH_2Cl_2 (3 × 10 mL); the combined organic phases were dried ($MgSO_4$) and concentrated. The residue was purified chromatographically [SiO_2; acetone/hexane (gradient from 2:8 to 3:7) and then acetone/CH_2Cl_2 (5:95)] to afford the rotaxane **20** as a sticky colorless liquid (55.6 mg, 89%). ^{1}H NMR (400 MHz, $CDCl_3$): δ = 7.73 (br, 2H), 7.40 (s, 1H), 7.36 (t, J = 1.6 Hz, 1H), 7.27 (d, J = 1.6 Hz, 2H), 7.09 (br, 1H), 7.00–6.82 (m, 9H), 6.79–6.59 (m, 4H), 4.33 (d, J = 11.2 Hz, 4H), 4.22 (d, J = 11.2 Hz, 4H), 3.97 (t, J = 6.4 Hz, 2H), 3.93–3.84 (m, 1H), 3.72–3.41 (m 16H), 2.75–2.50 (m, 2H), 2.20 (d, J = 4.8 Hz, 1H), 1.93–1.77 (m, 2H), 1.77–1.54 (m, 4H), 1.30 (s, 18H); ^{13}C NMR (100 MHz, $CDCl_3$): δ = 154.5, 150.8, 150.6, 141.7, 136.7, 131.7 (br), 131.1 (q, $^2J_{CF}$ = 32.6 Hz), 128.3, 125.9, 123.6 (q, $^1J_{CF}$ = 271.0 Hz), 122.4, 122.2, 121.5 (br), 118.6 (br), 114.3, 114.0 (br), 84.6, 84.1, 73.3, 70.6, 70.1, 68.8, 68.2, 36.1, 34.7, 31.3, 29.3, 28.6, 22.4; HR-MS (ESI): calcd for [**20** + Na]$^+$, $C_{61}H_{74}F_6N_2NaO_9^+$: m/z 1115.5191; found 1115.5218.

References:

1. Lin, C.-F.; Lai, C.-C.; Liu, Y.-H.; Peng, S.-M.; Chiu, S.-H. Use of anions to allow translational isomerism of a rotaxane, *Chem. Eur. J.* **2007**, *13*, 4350–4355.
2. Yang, L.-L.; Li, G.-B.; Ma, S.; Zou, C.; Zhou, S.; Sun, Q.-Z.; Cheng, C.; Chen, X.; Wang, L.-*J*.; Feng, S.; Li, L.-L.; Yang, S.-Y. Structure–activity relationship studies of pyrazolo[3,4-d]pyrimidine derivatives leading to the discovery of a novel multikinase inhibitor that potently inhibits FLT3 and VEGFR2 and evaluation of its activity against acute myeloid leukemia in vitro and in vivo, *J. Med. Chem.* **2013**, *56*, 1641–1655.
3. Cheng, P.-N.; Lin, C.-F.; Liu, Y.-H.; Lai, C.-C.; Peng, S.-M.; Chiu, S.-H. [3]Pseudorotaxane-like complexes formed between bipyridinium dications and bis-p-xylyl[26]crown-6, *Org. Lett.* **2006**, *8*, 435–438.
4. Monreal, M. *J*.; Thomson, R. K.; Cantat, T.; Travia, N. E.; Scott, B. L.; Kiplinger, J. L. UI_4(1,4-dioxane)$_2$, [UCl_4(1,4-dioxane)]$_2$, and UI_3(1,4-dioxane)$_{1.5}$: stable and versatile starting materials for low- and high-valent uranium chemistry, *Organometallics* **2011**, *30*, 2031–2038.
5. Peddibhotla, S.; Shi, R.; Khan, P.; Smith, L. H.; Mangravita-Novo, A.; Vicchiarelli, M.; Su, Y.; Okolotowicz, K. J.; Cashman, J. R.; Reed, J. C.; Roth, G. P. Inhibition of protein kinase C-driven nuclear factor-κB activation: synthesis, structure−activity relationship, and pharmacological profiling of pathway specific benzimidazole probe molecules, *J. Med. Chem.* **2010**, *53*, 4793–4797.
6. Uraguchi, D.; Kinoshita, N.; Kizu, T.; Ooi, T. Synergistic catalysis of ionic Brønsted acid and photosensitizer for a redox neutral asymmetric α-coupling of *N*-arylaminomethanes with aldimines, *J. Am. Chem. Soc.* **2015**, *137*, 13768–13771.
7. Stoll, R. S.; Peters, M. V.; Kuhn, A.; Heiles, S.; Goddard, R.; Bühl, M.; Thiele, C. M.; Hecht, S. Photoswitchable catalysts: correlating structure and conformational dynamics with reactivity by a combined experimental and computational approach, *J. Am. Chem. Soc.* **2009**, *131*, 357–367.

Figure S1. ^{1}H NMR Spectrum (400 MHz / $CDCl_3$ / 298 K) of **1**·TFPB

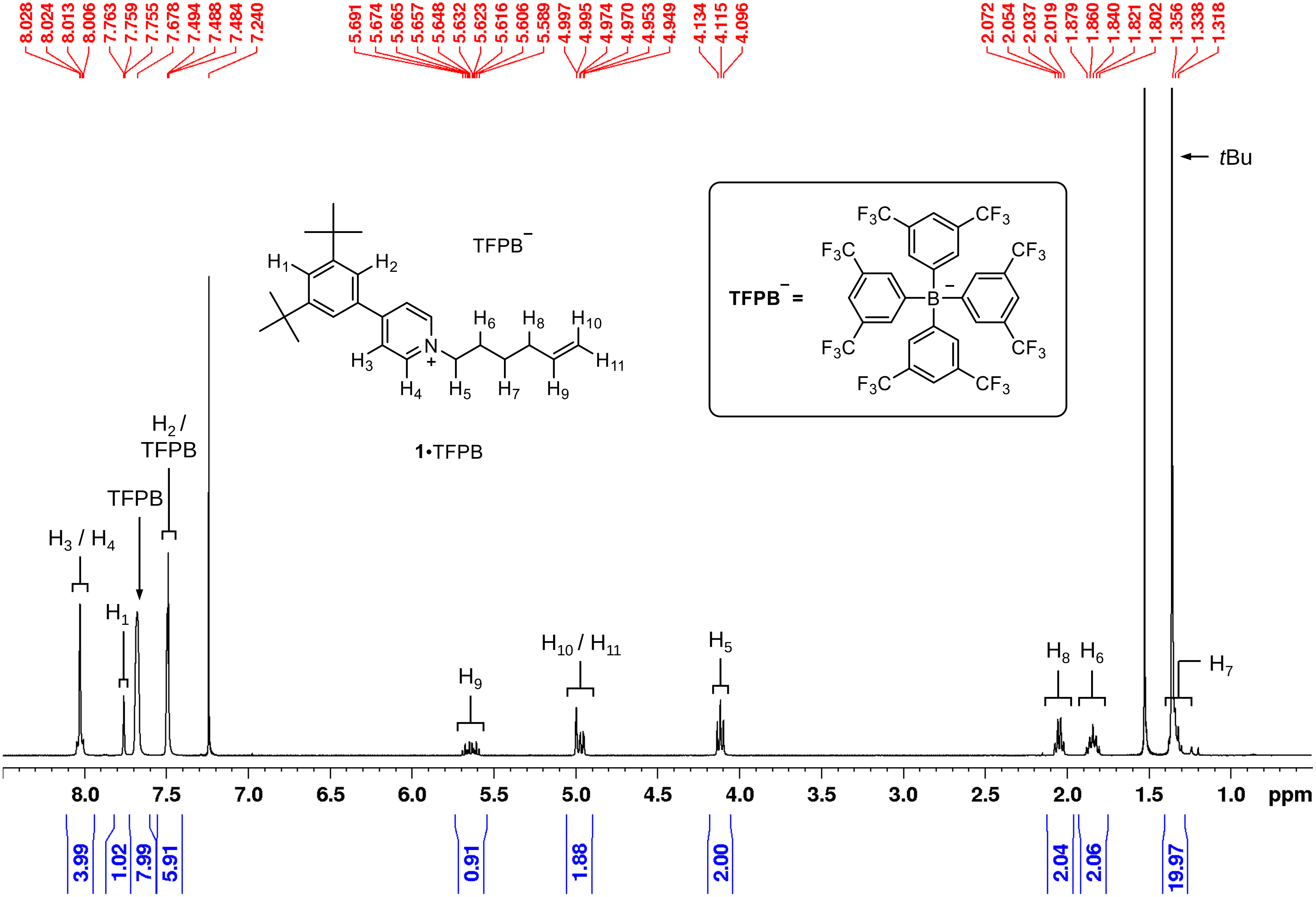

Figure S2. ^{13}C NMR Spectrum (100 MHz / CD_2Cl_2 / 298 K) of **1**·TFPB

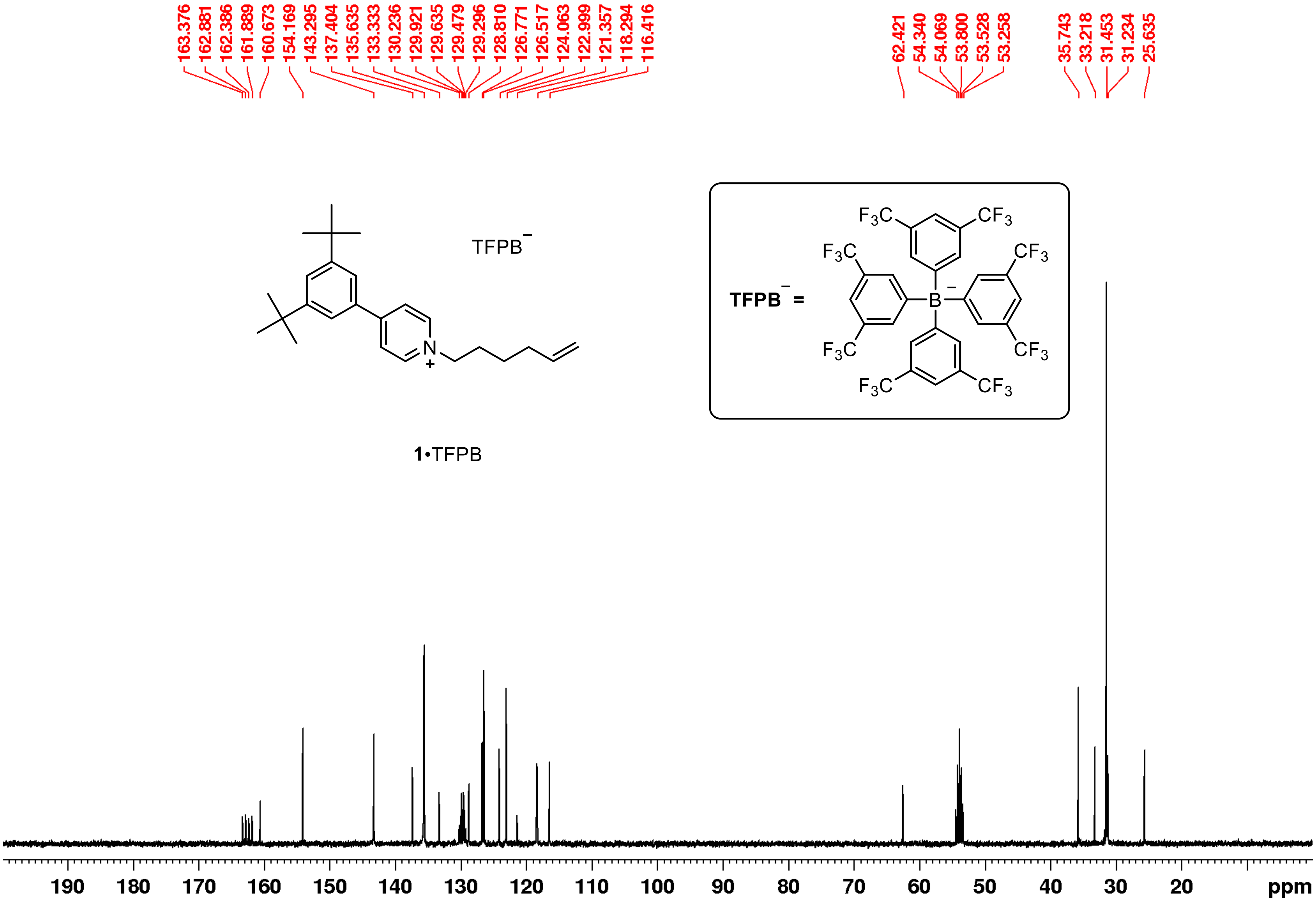

Figure S3. ^{1}H NMR Spectrum (400 MHz / $(CD_3)_2CO$ / 298 K) of **2**

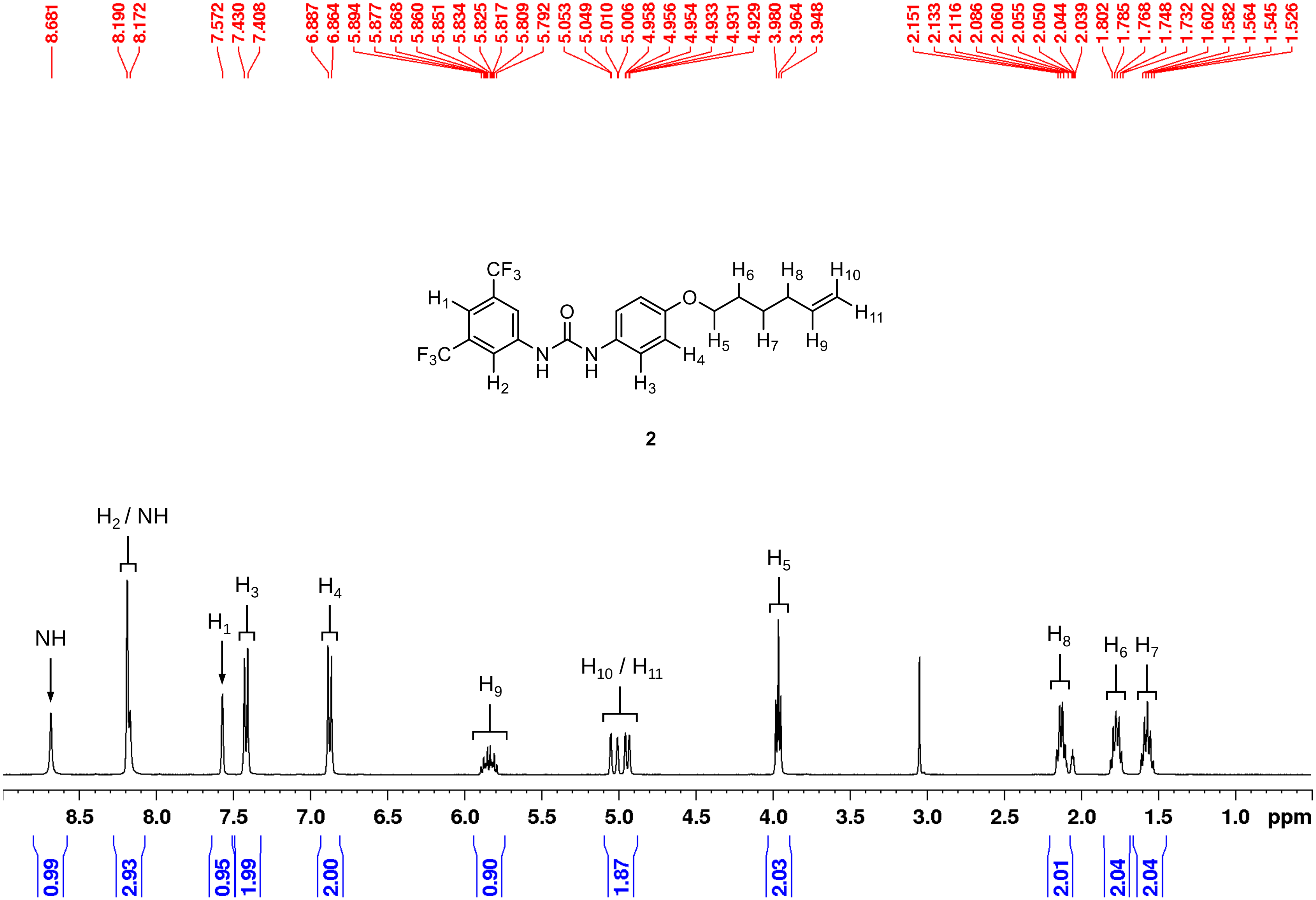

Figure S4. ^{13}C NMR Spectrum (100 MHz / $(CD_3)_2CO$ / 298 K) of **2**

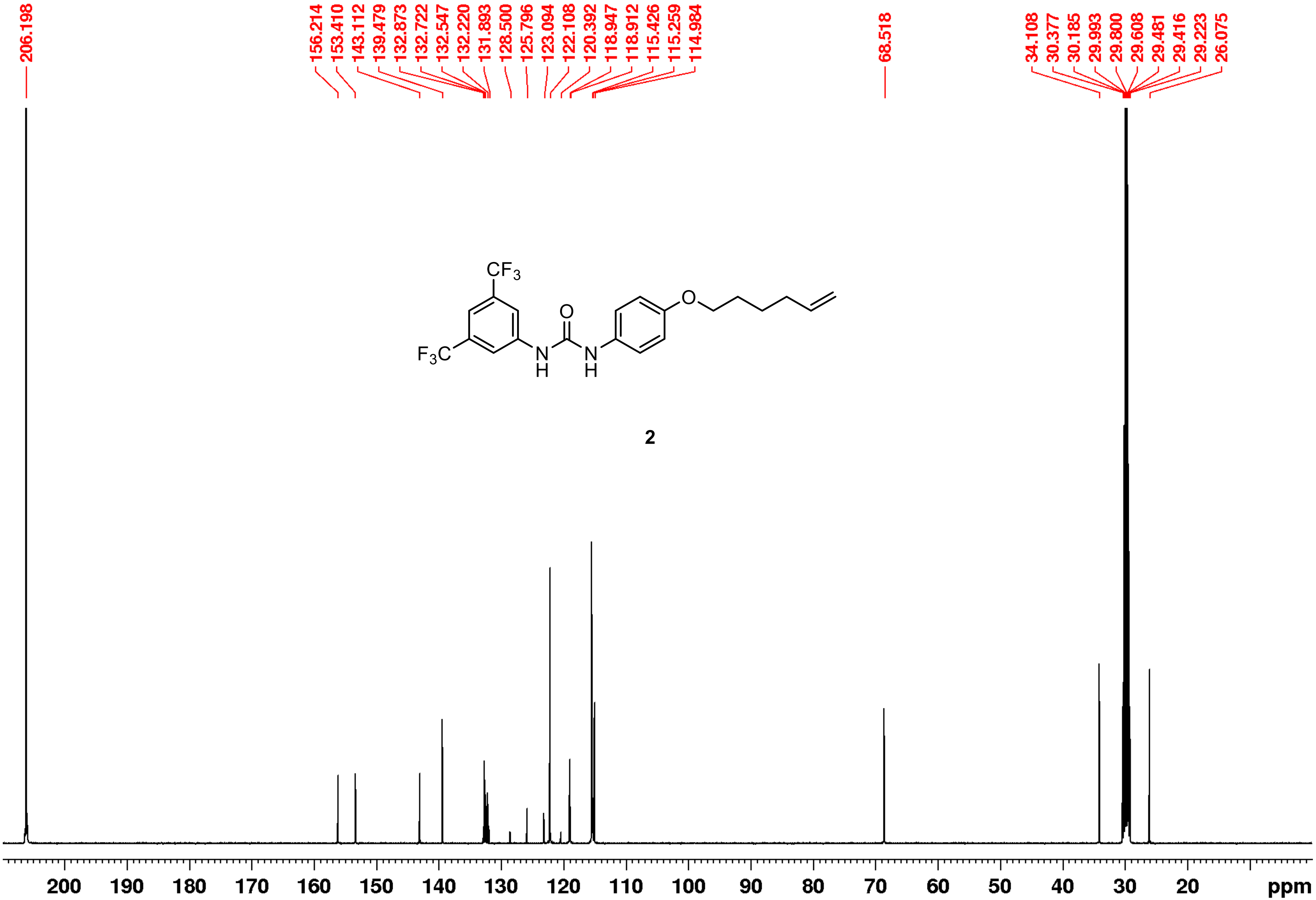

Figure S5. ^{1}H NMR Spectrum (400 MHz / CD_2Cl_2 / 298 K) of **7**·TFPB

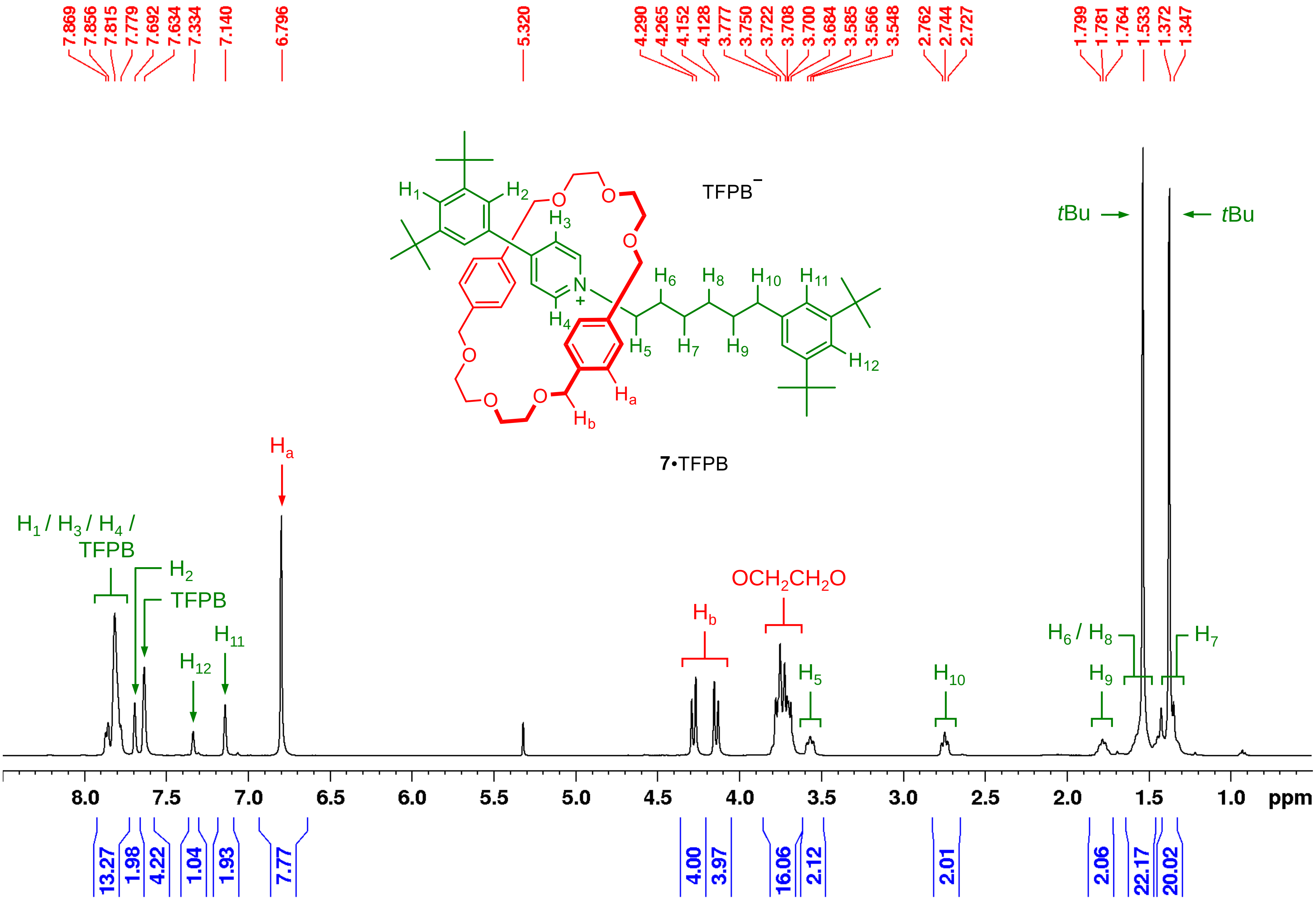

Figure S6. ^{13}C NMR Spectrum (100 MHz / CD_2Cl_2 / 298 K) of **7**·TFPB

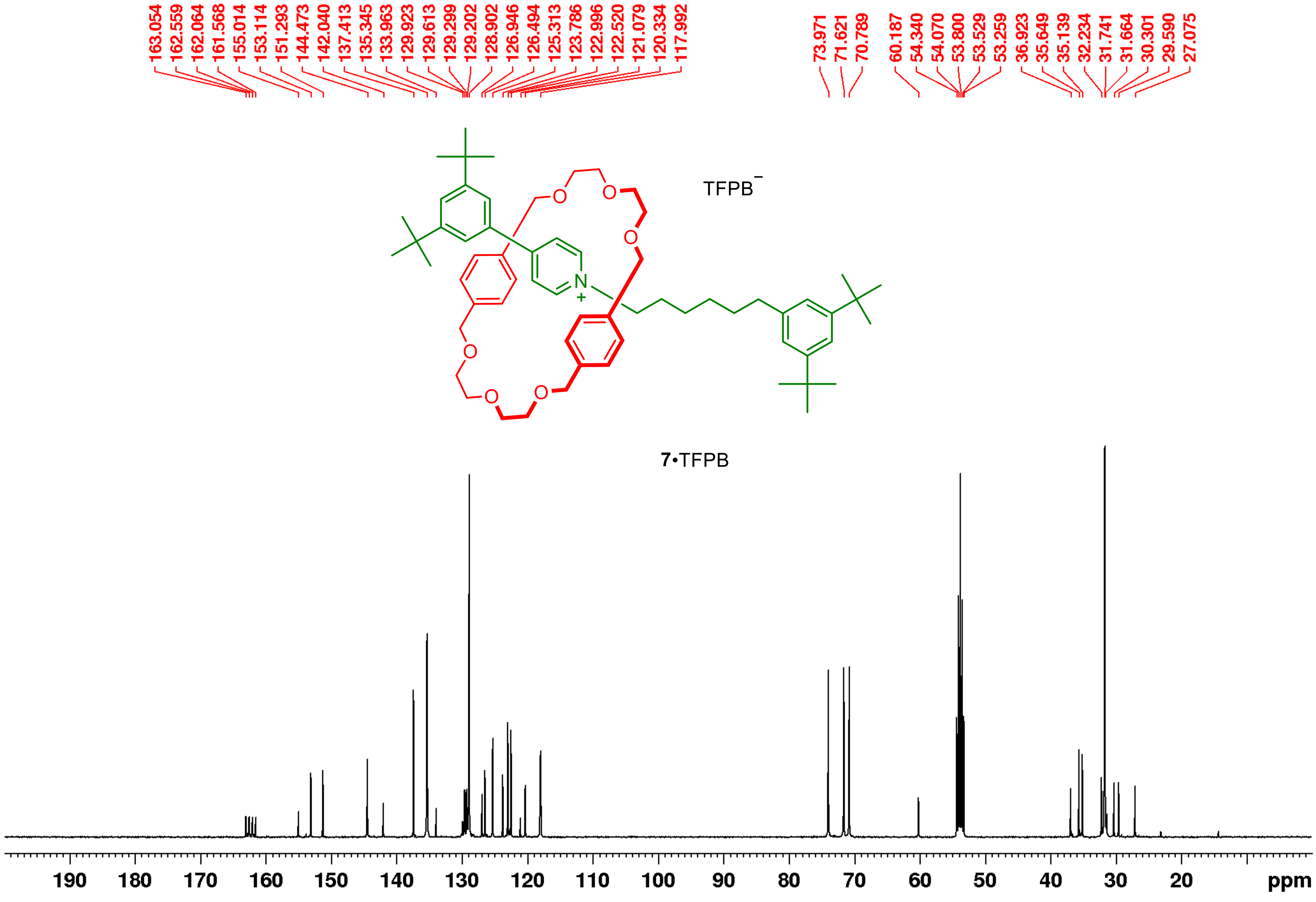

Figure S7. ^{1}H NMR Spectrum (400 MHz / CD_2Cl_2 / 298 K) of **9**

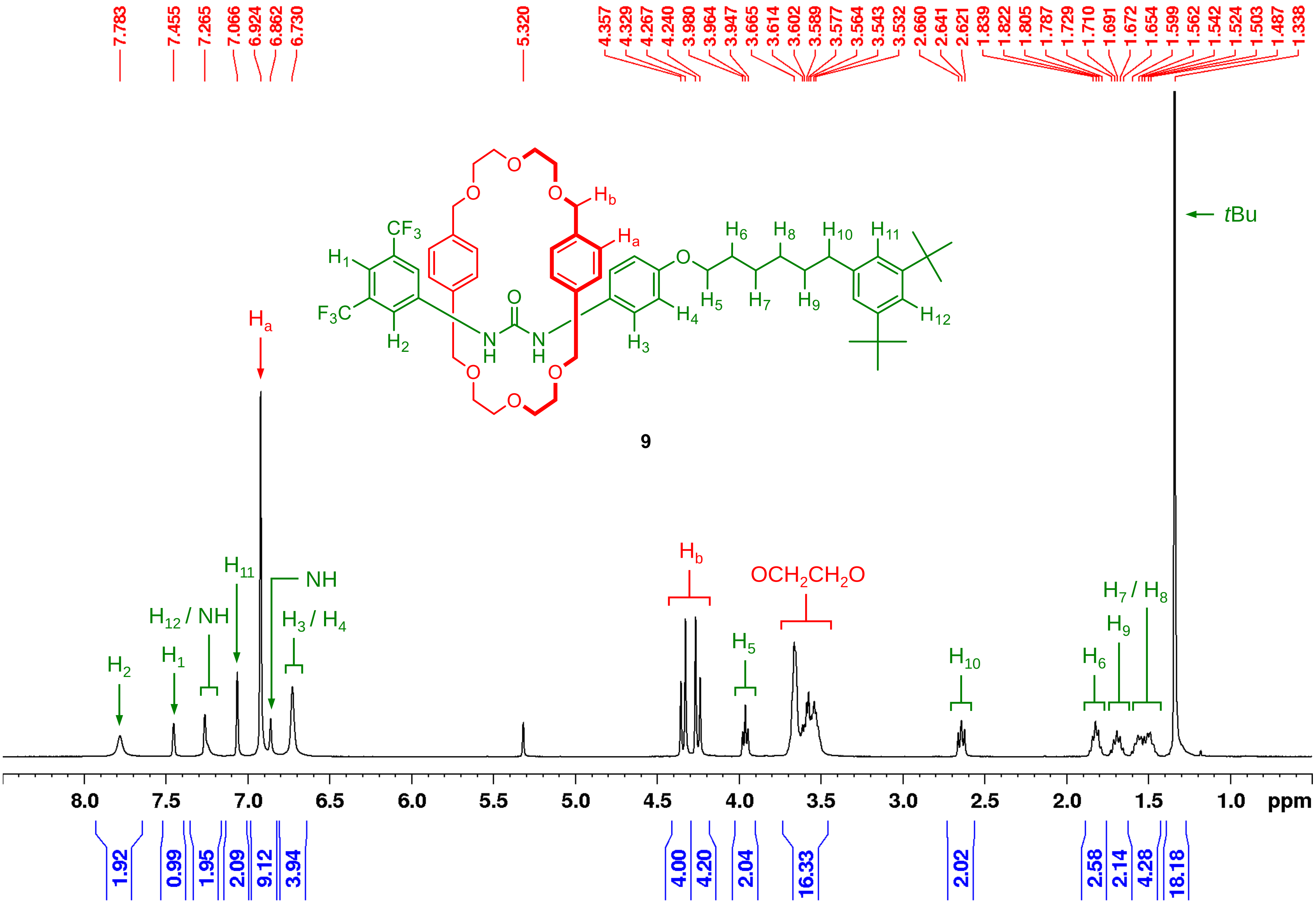

Figure S8. ^{13}C NMR Spectrum (100 MHz / CD_2Cl_2 / 298 K) of **9**

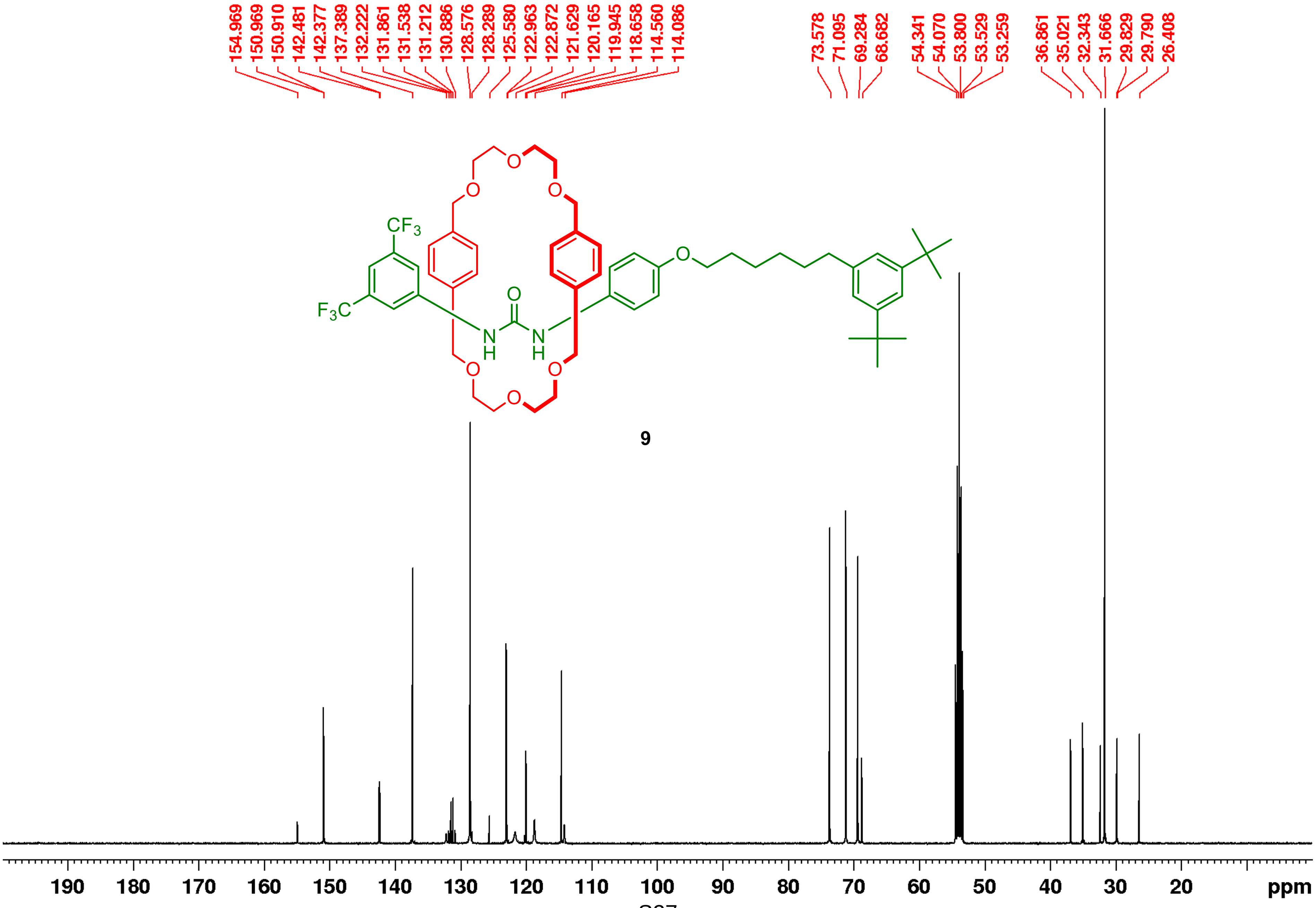

Figure S9. ^{1}H NMR Spectrum (800 MHz / $CDCl_3$ / 298 K) of **12**·TFPB

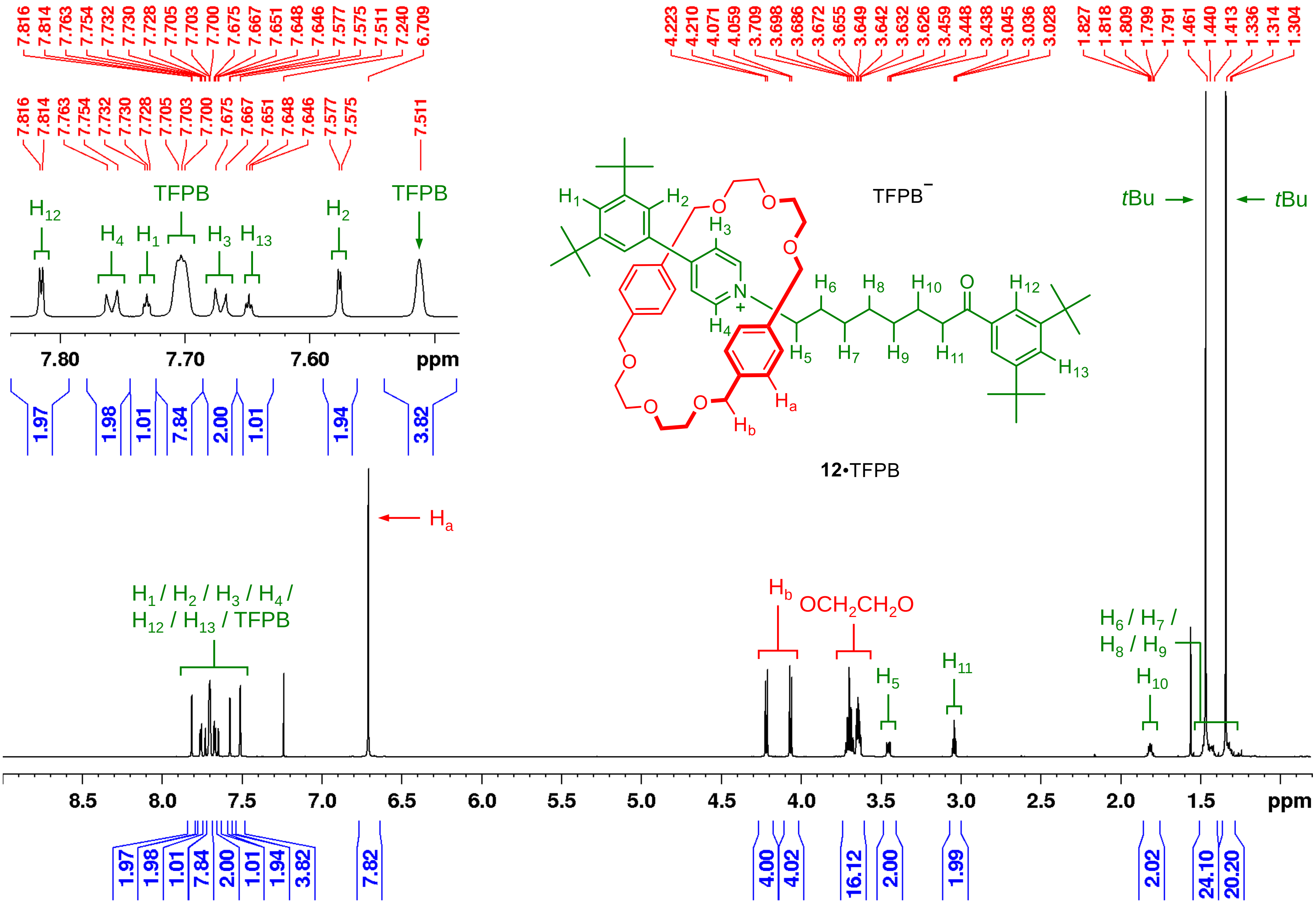

Figure S10. ^{13}C NMR Spectrum (200 MHz / $CDCl_3$ / 298 K) of **12**·TFPB

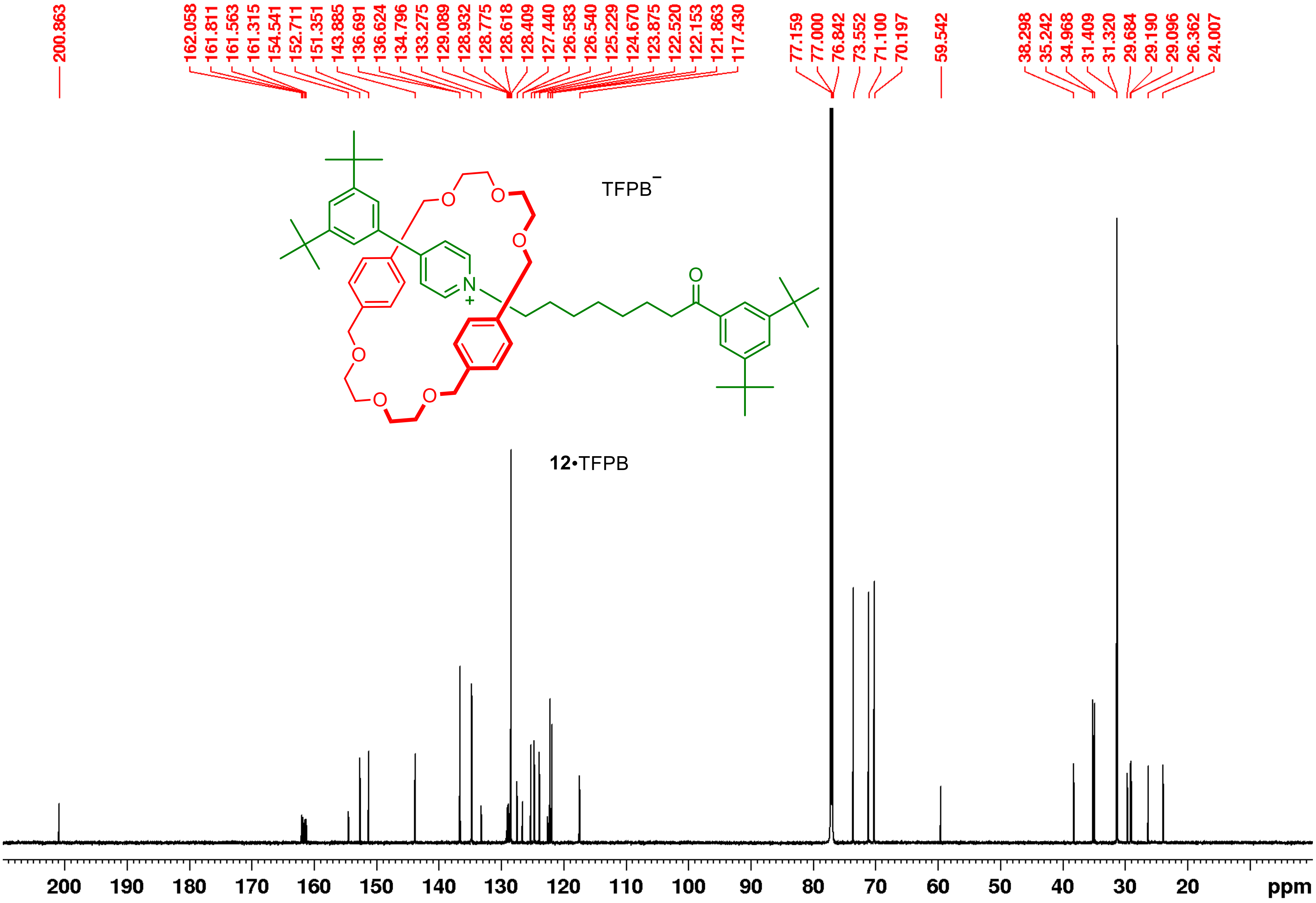

Figure S11. ^{1}H NMR Spectrum (500 MHz / CD_2Cl_2 / 298 K) of **13**

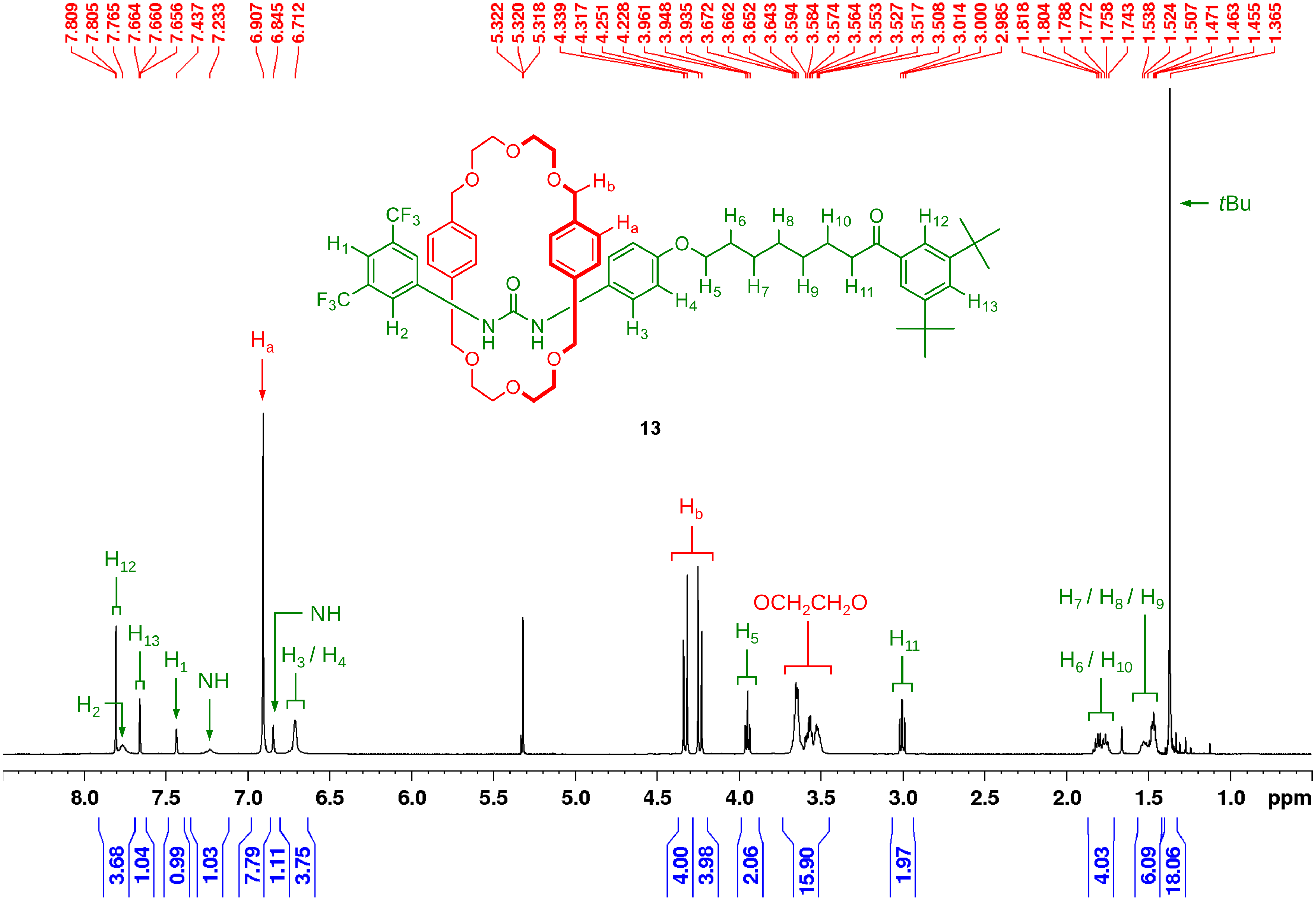

Figure S12. ^{13}C NMR Spectrum (100 MHz / $CDCl_3$ / 298 K) of **13**

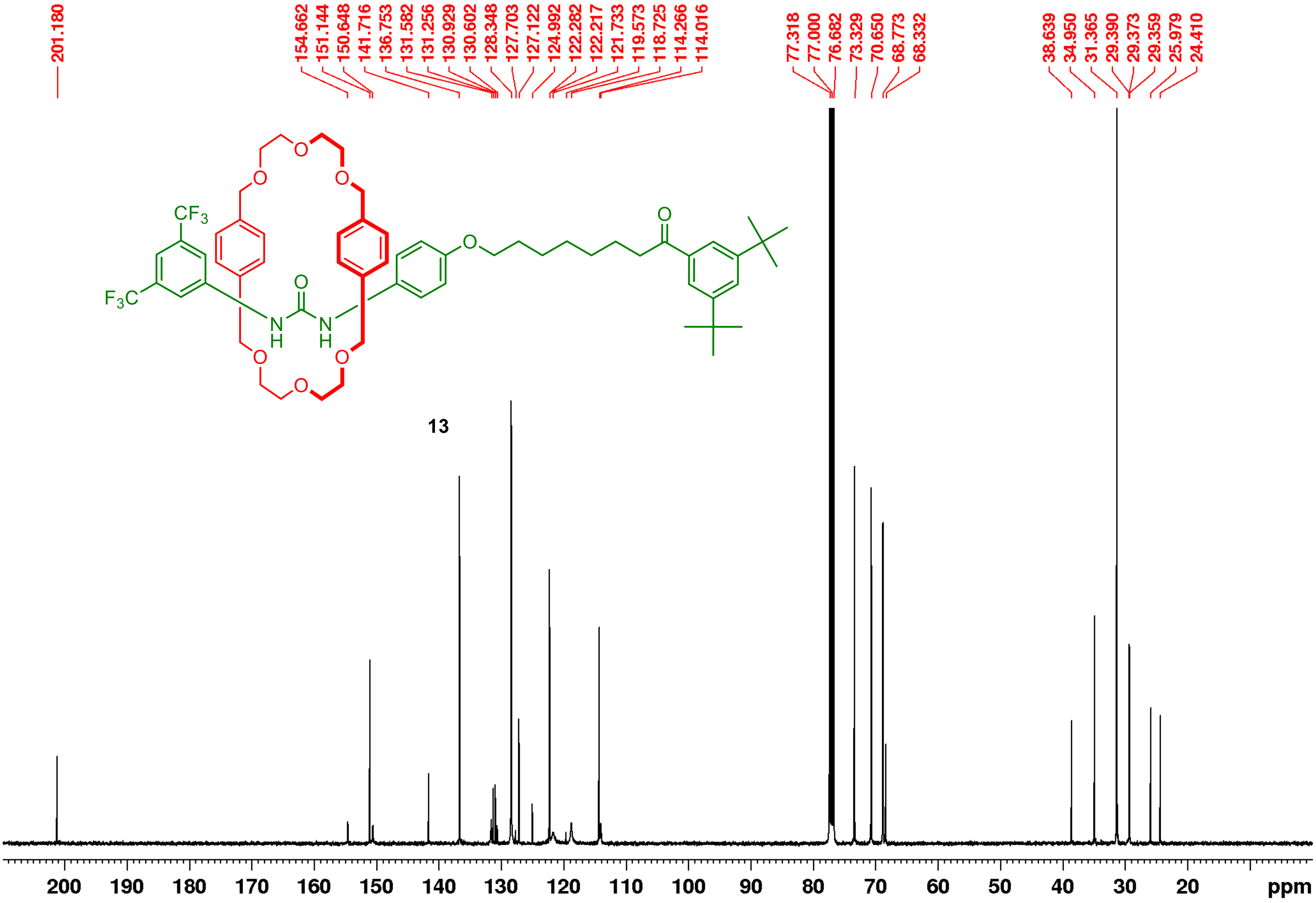

Figure S13. ^{1}H NMR Spectrum (400 MHz / CD_2Cl_2 / 298 K) of **S1**·TFPB

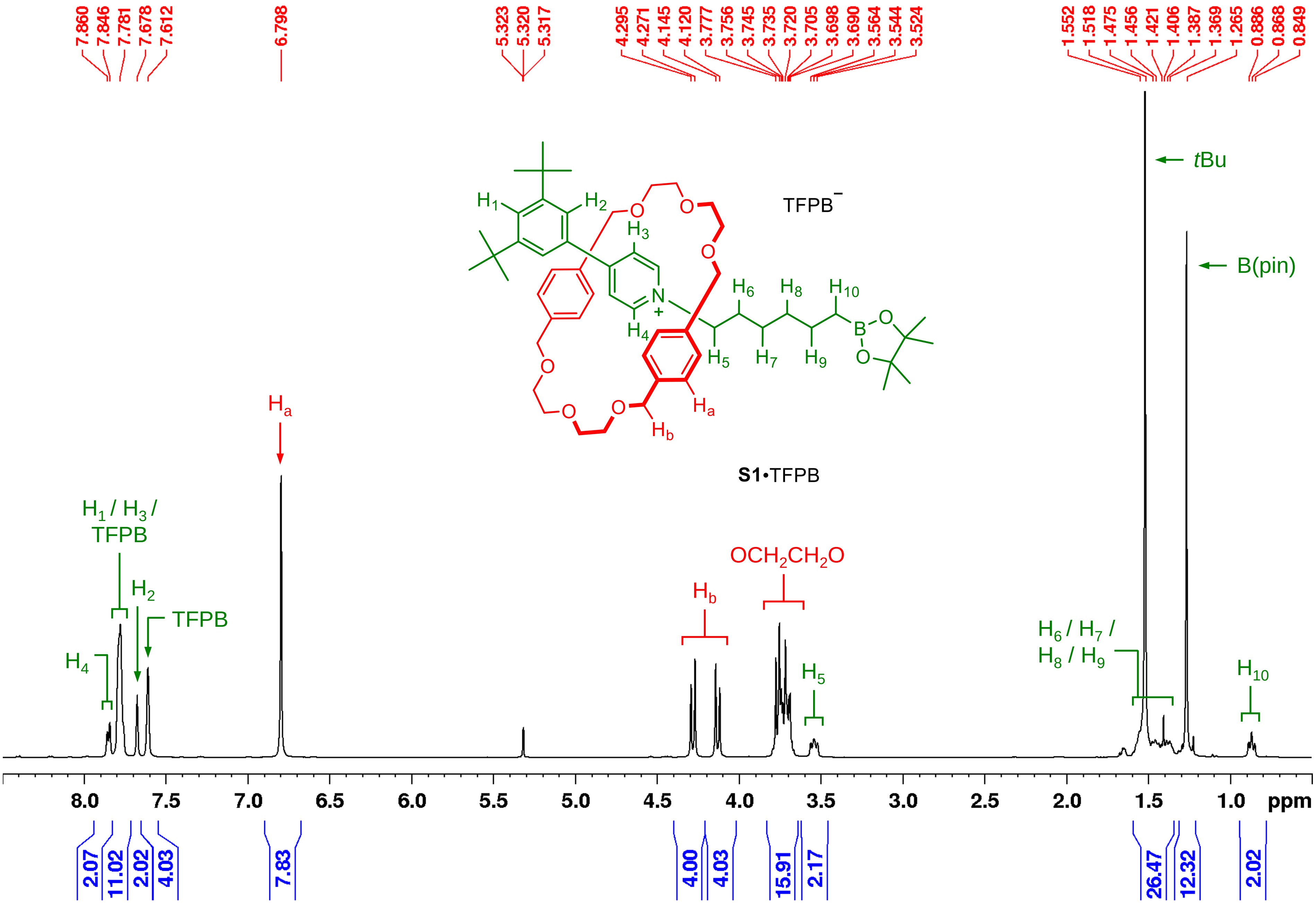

Figure S14. ^{13}C NMR Spectrum (100 MHz / CD_2Cl_2 / 298 K) of **S1**·TFPB

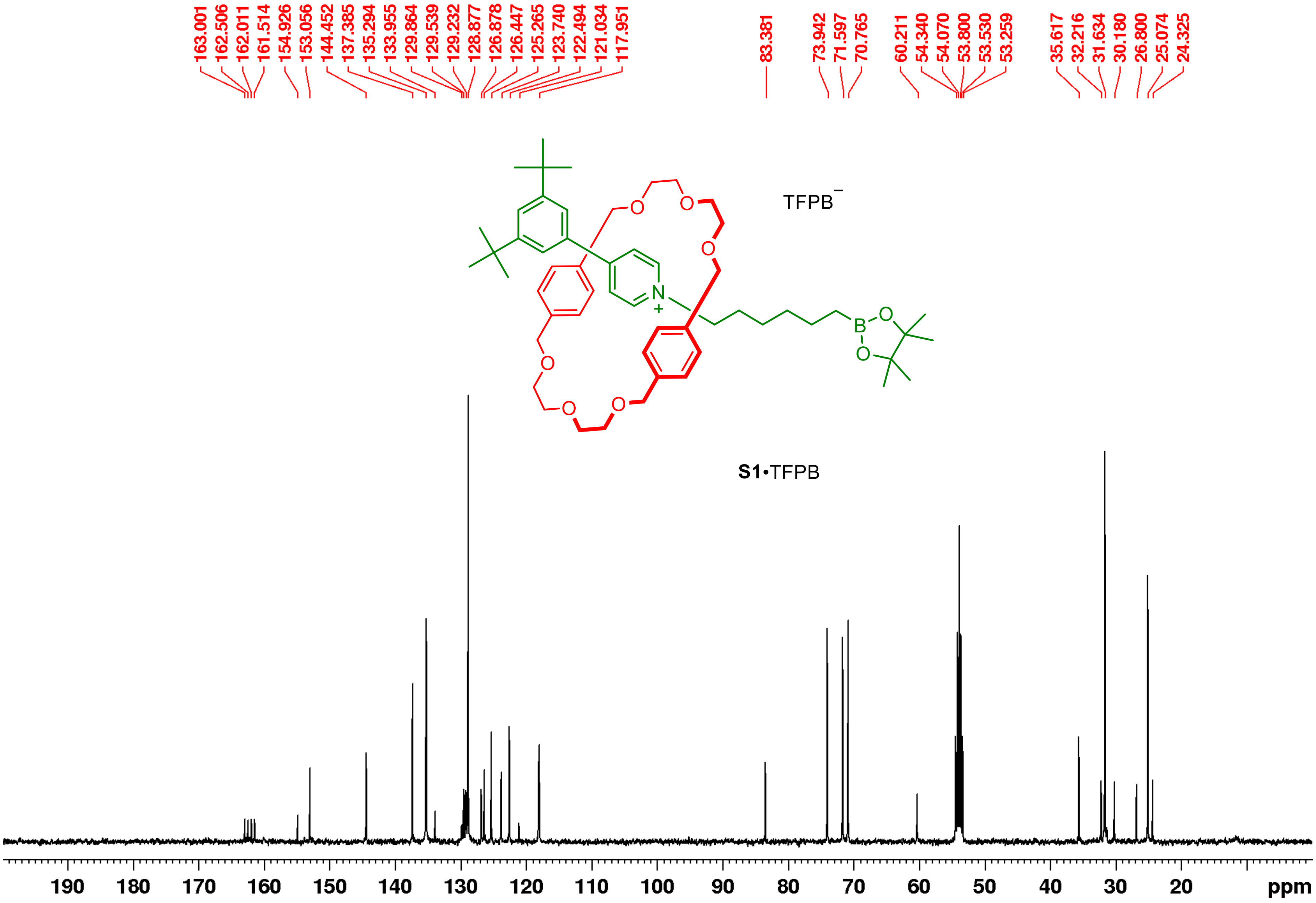

Figure S15. ^{1}H NMR Spectrum (400 MHz / CD_2Cl_2 / 298 K) of **14**·TFPB

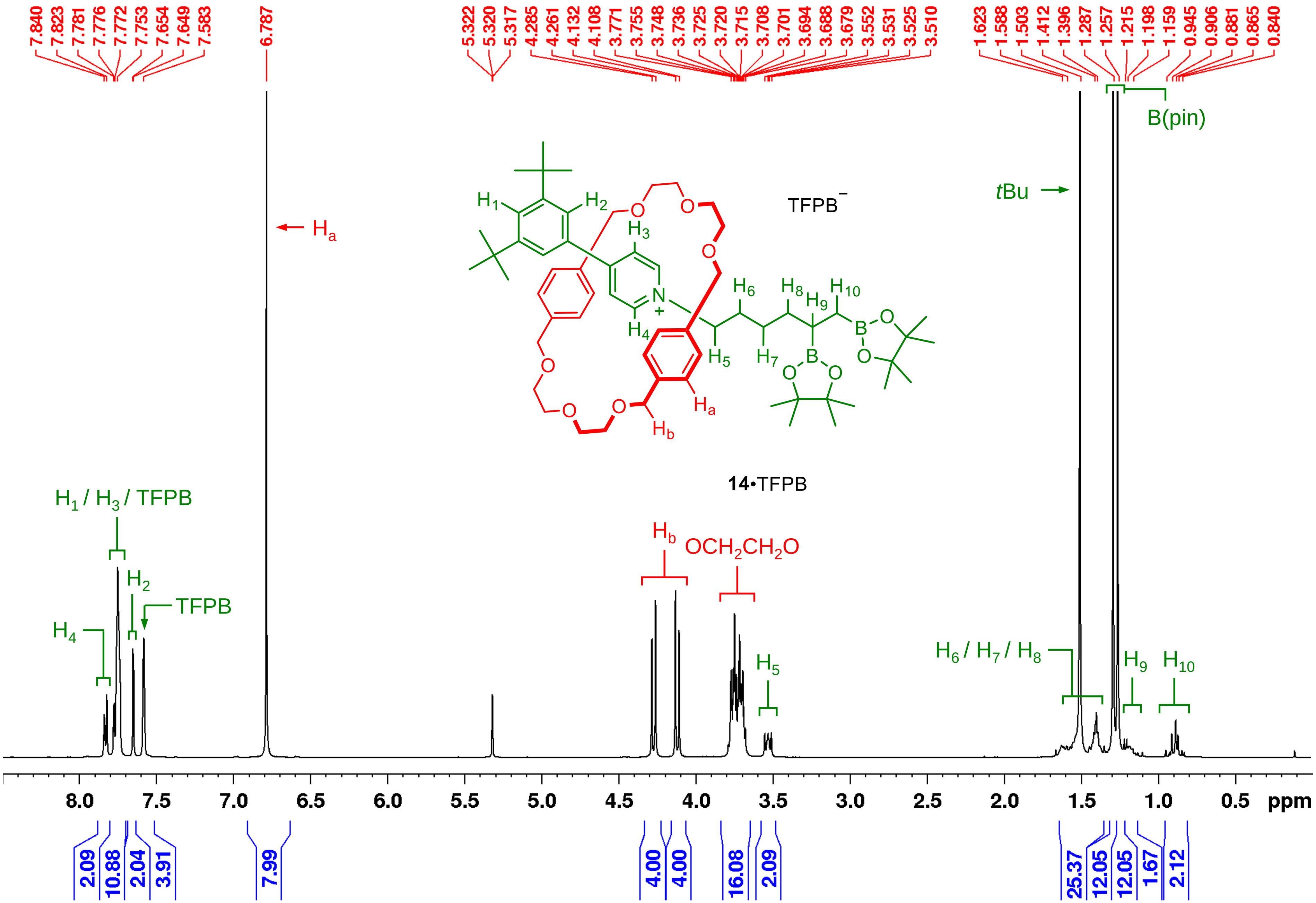

Figure S16. ^{13}C NMR Spectrum (100 MHz / CD_2Cl_2 / 298 K) of **14**·TFPB

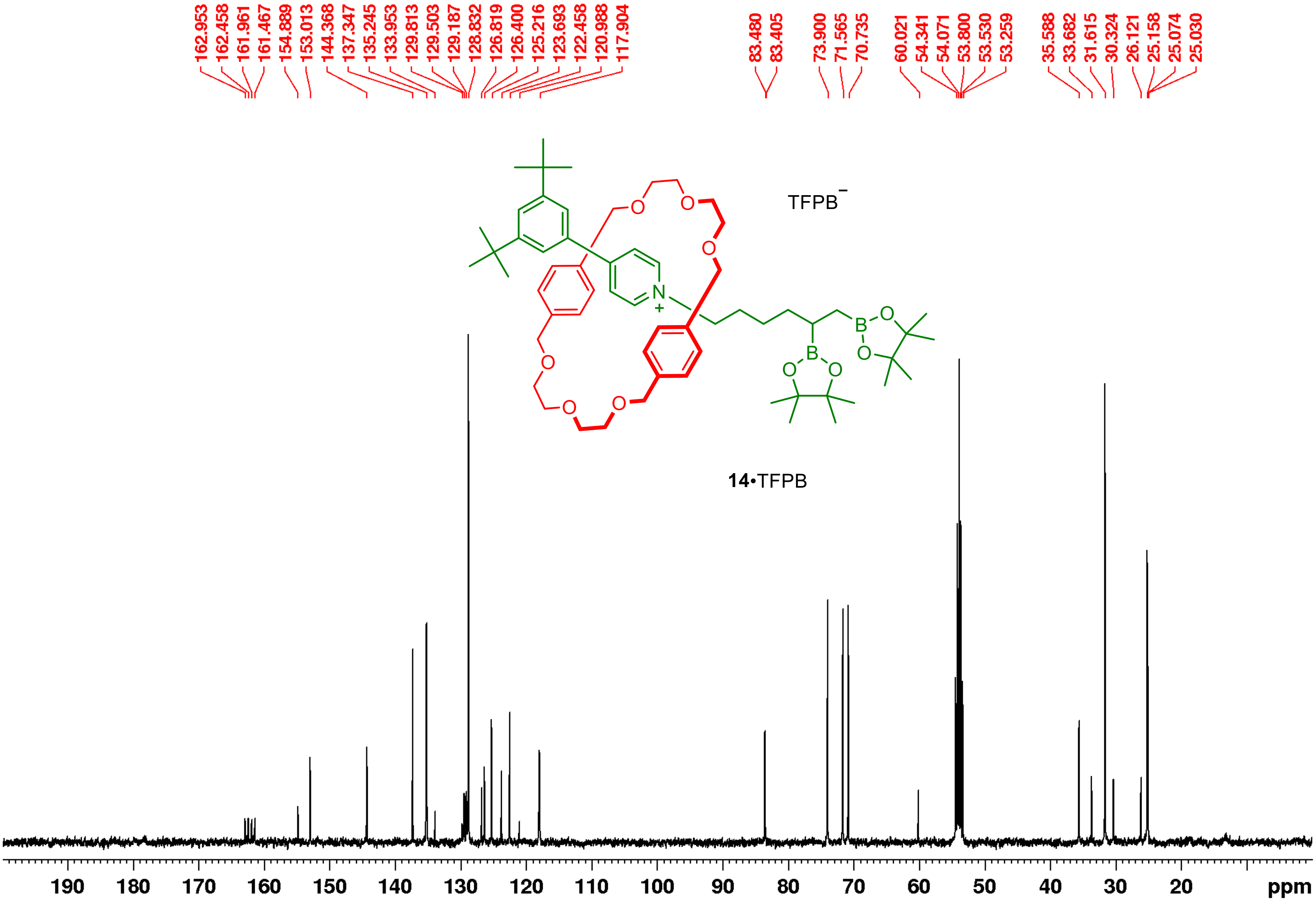

Figure S17. ^{1}H NMR Spectrum (400 MHz / CD_2Cl_2 / 298 K) of **S2**·TFPB

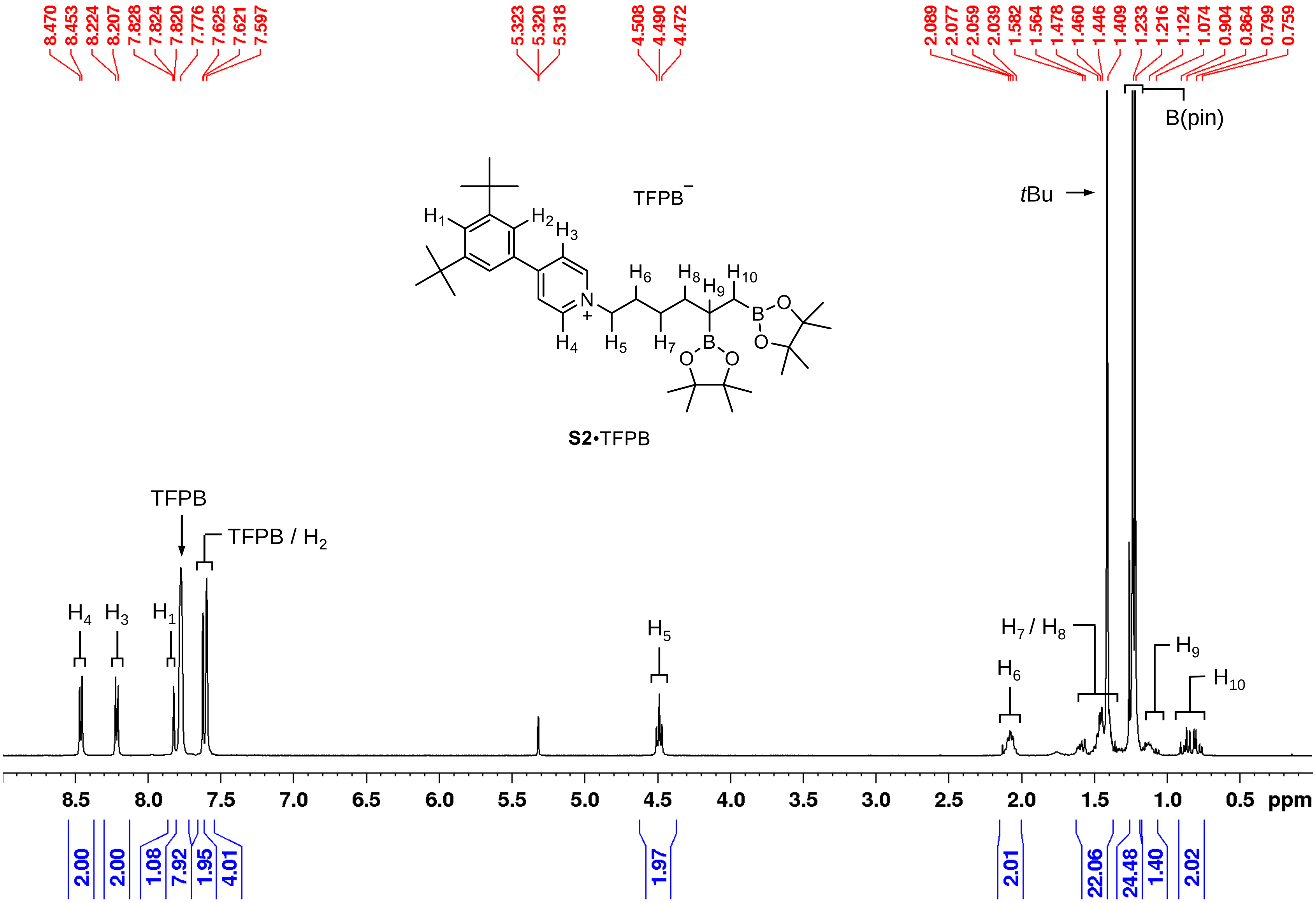

Figure S18. ^{13}C NMR Spectrum (100 MHz / CD_2Cl_2 / 298 K) of **S2**·TFPB

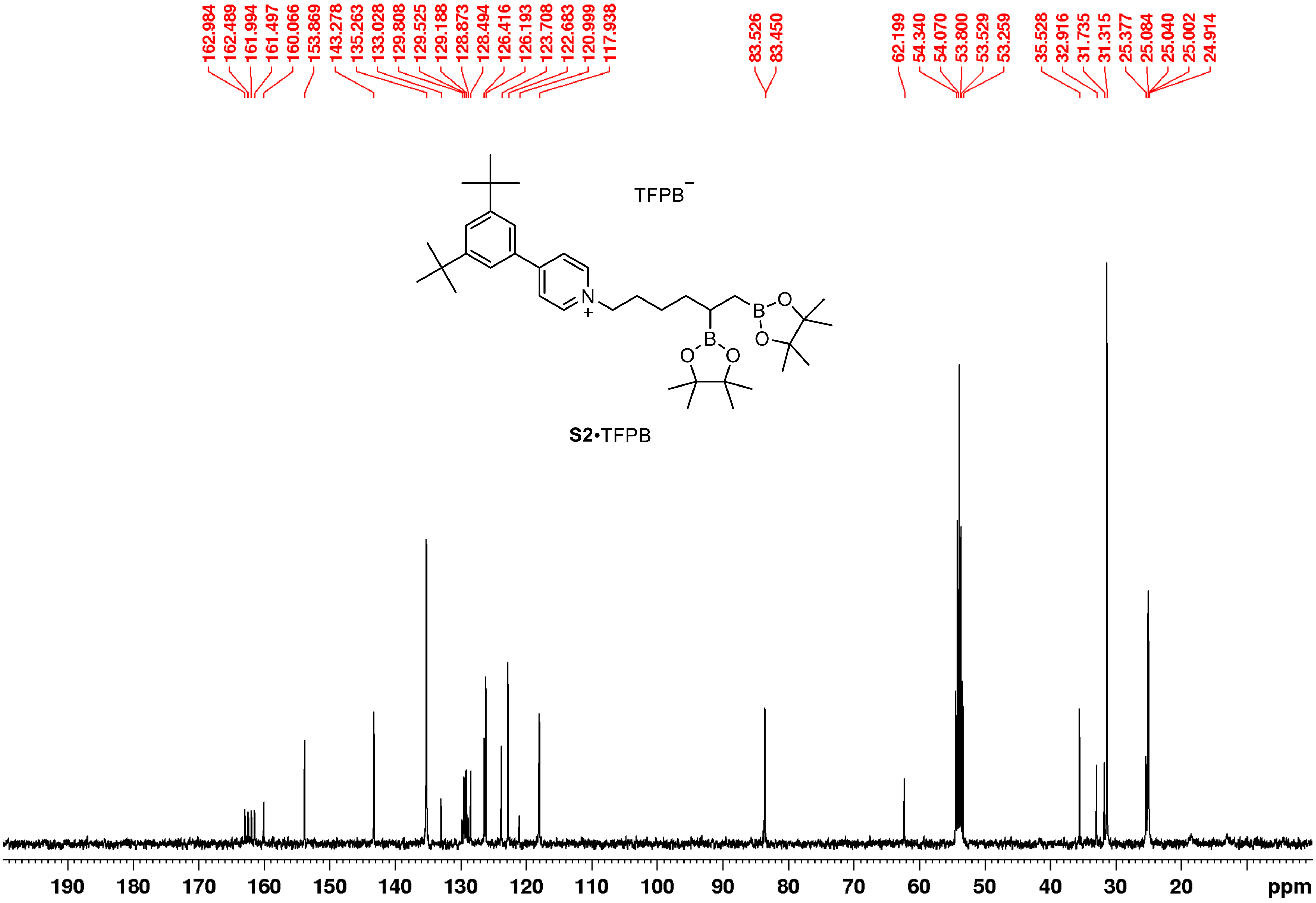

Figure S19. ^{1}H NMR Spectrum (800 MHz / CD_2Cl_2 / 298 K) of **15**

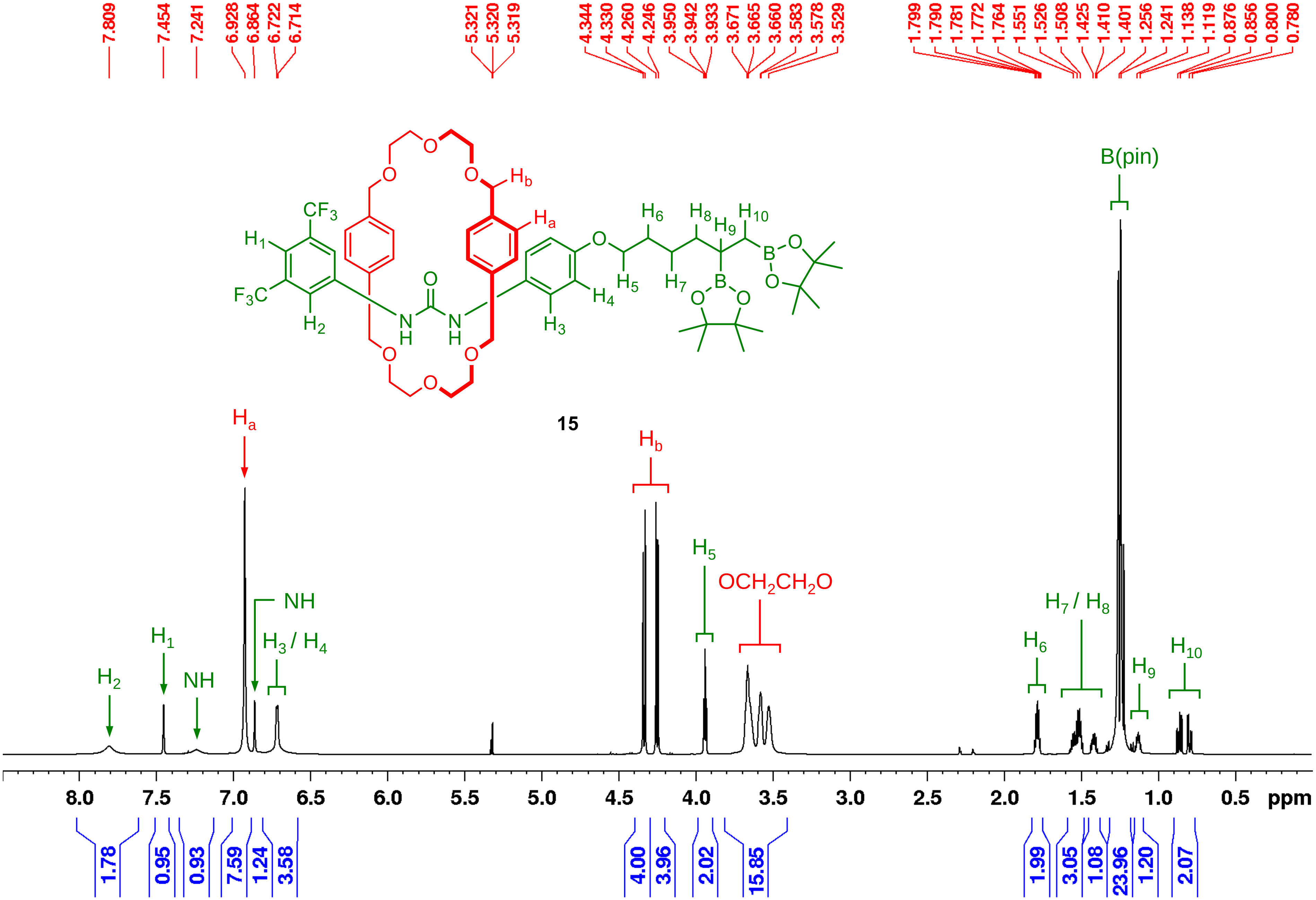

Figure S20. ^{13}C NMR Spectrum (100 MHz / $CDCl_3$ / 298 K) of **15**

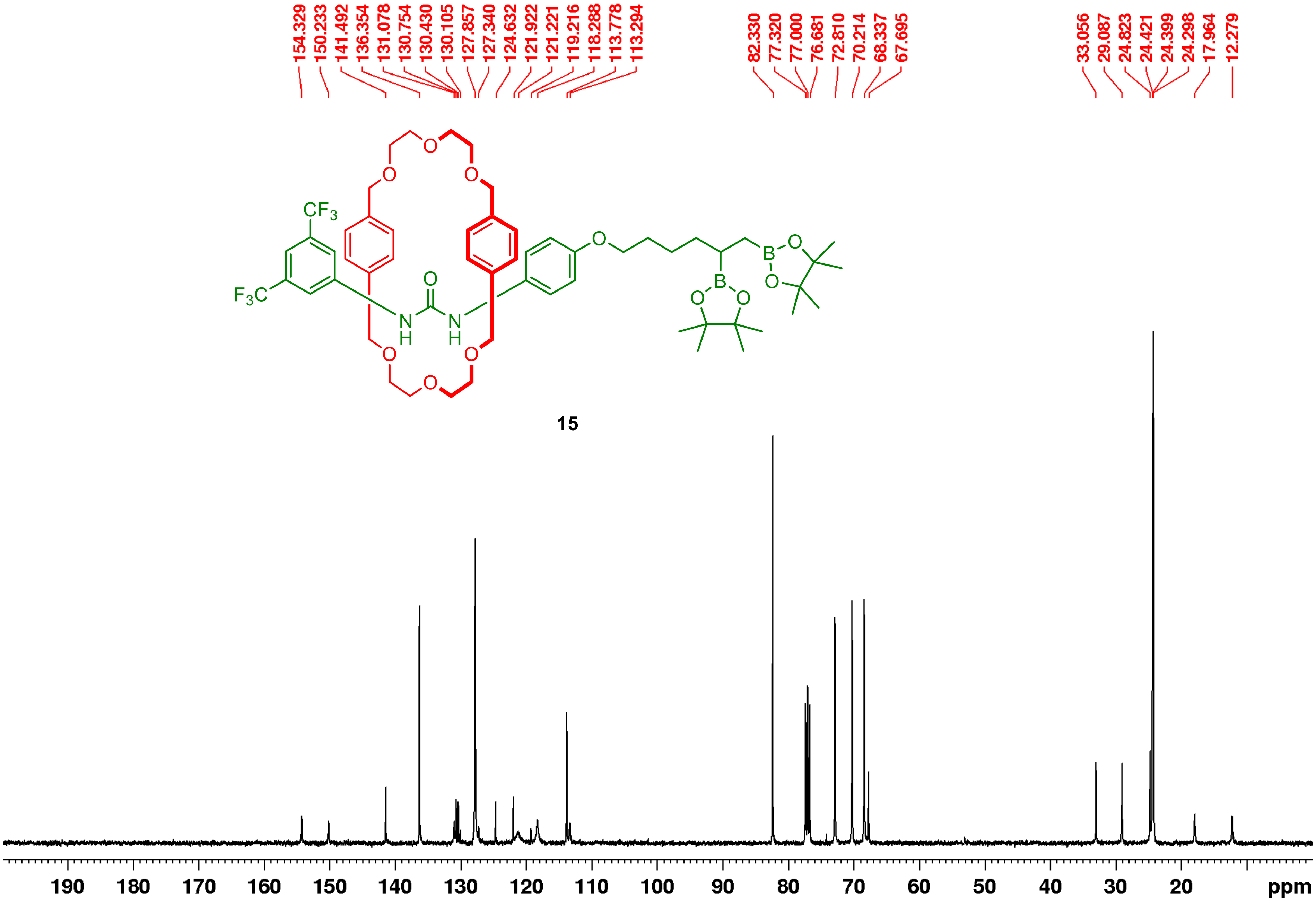

Figure S21. ^{1}H NMR Spectrum (400 MHz / $CDCl_3$ / 298 K) of **S3**

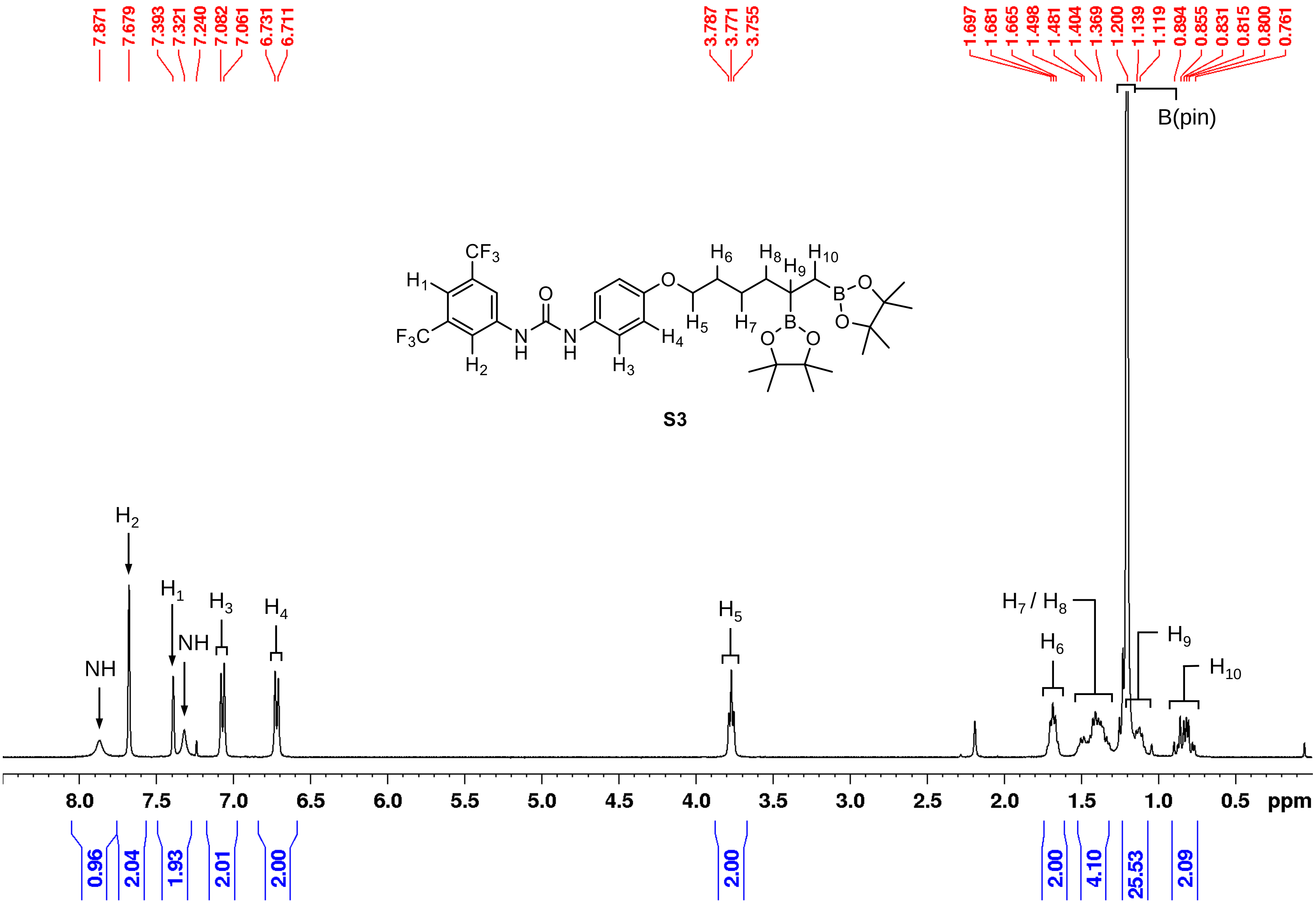

Figure S22. ^{13}C NMR Spectrum (100 MHz / $CDCl_3$ / 298 K) of **S3**

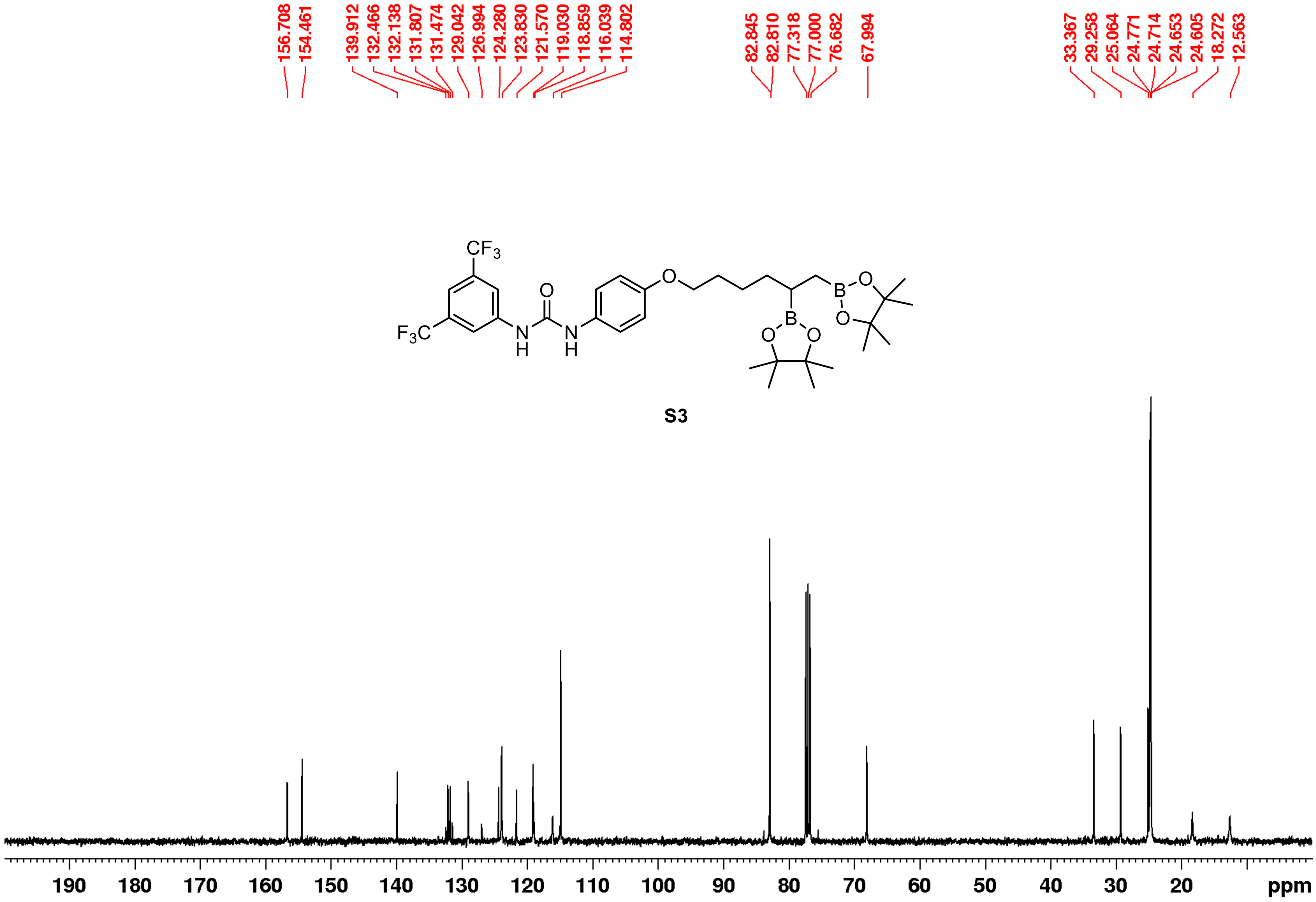

Figure S23. ^{1}H NMR Spectrum (400 MHz / $CDCl_3$ / 298 K) of **16**

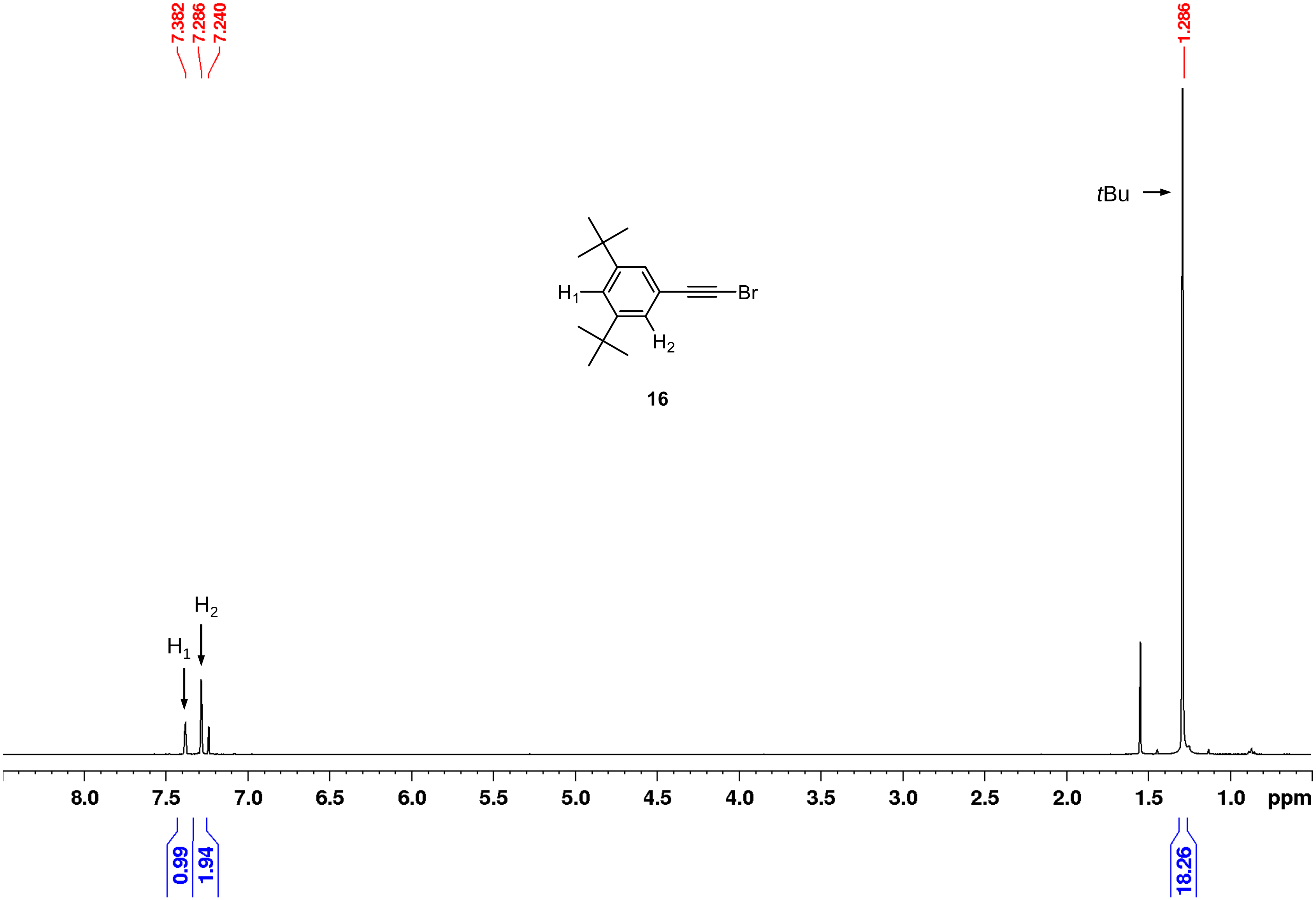

Figure S24. ^{13}C NMR Spectrum (100 MHz / $CDCl_3$ / 298 K) of **16**

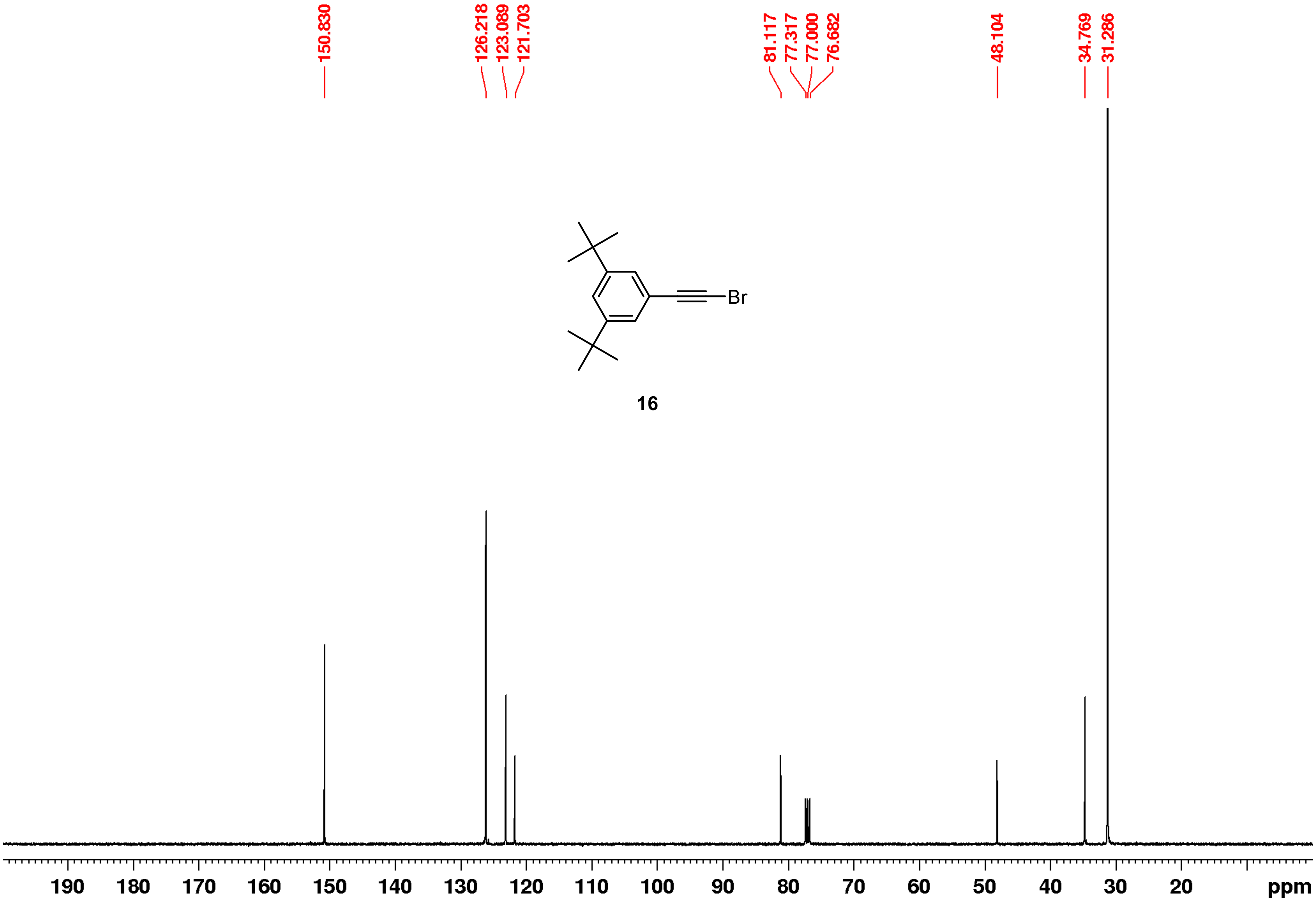

Figure S25. ^{1}H NMR Spectrum (400 MHz / $CDCl_3$ / 298 K) of **17**·TFPB

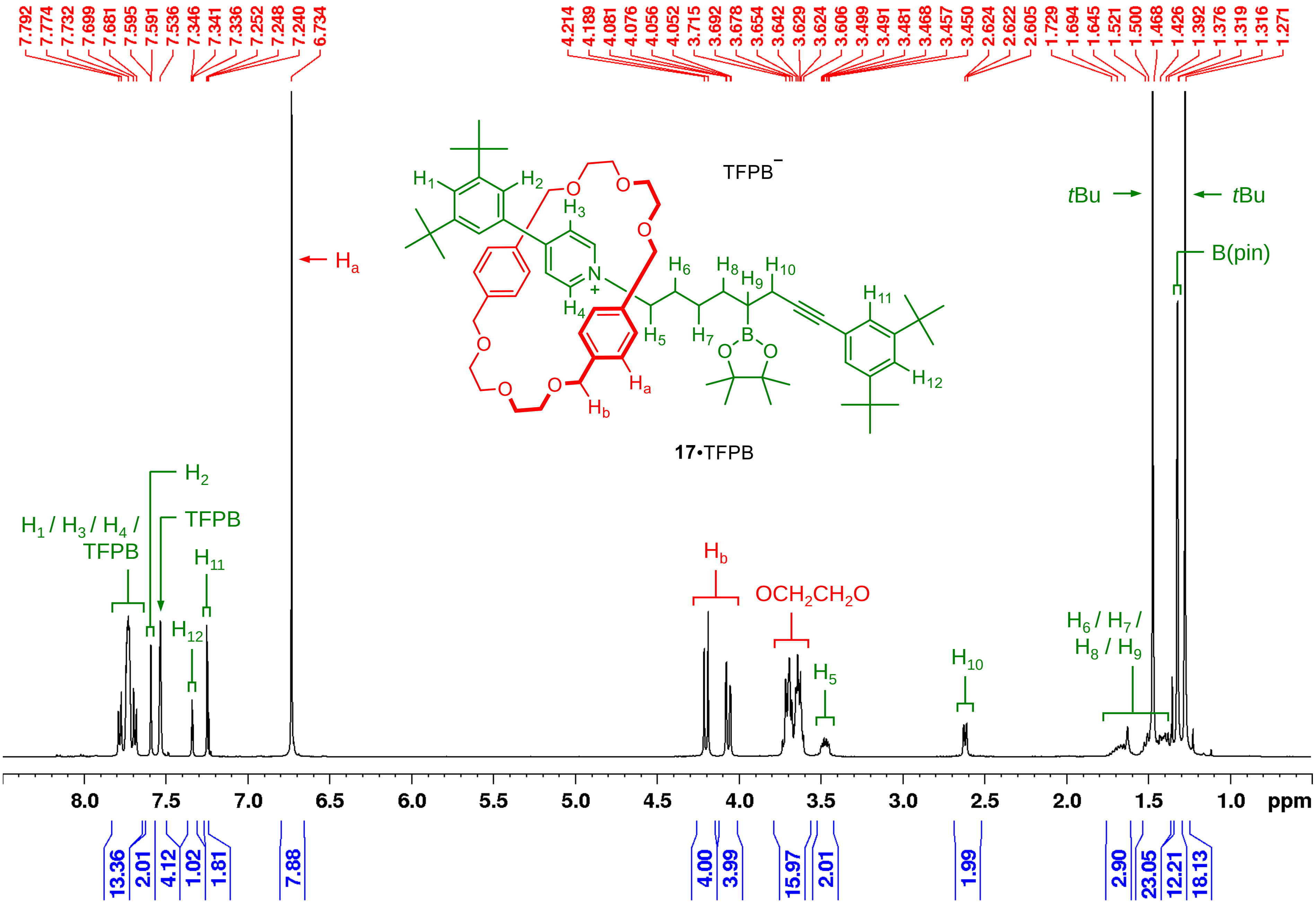

Figure S26. ^{13}C NMR Spectrum (100 MHz / $CDCl_3$ / 298 K) of **17**·TFPB

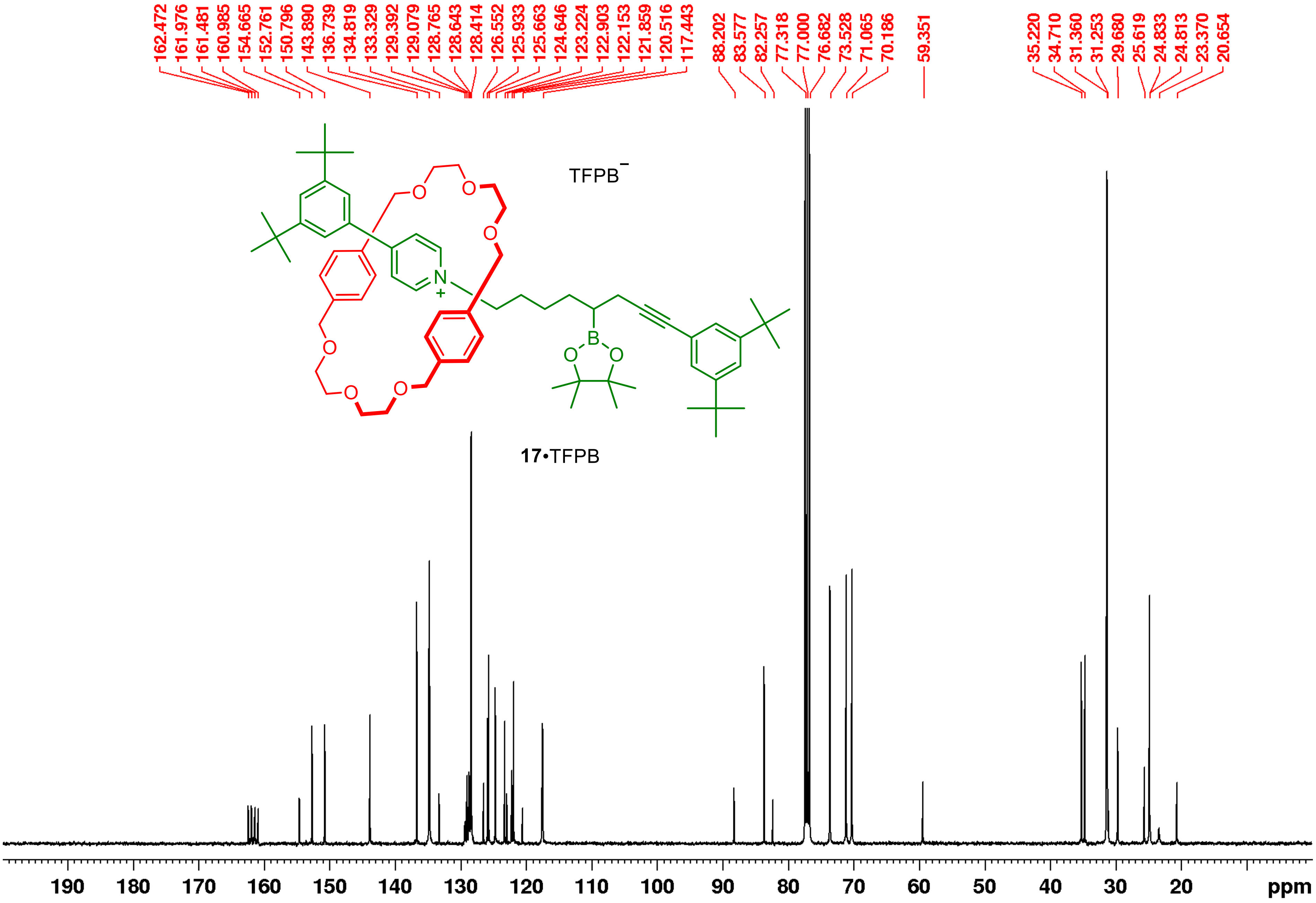

Figure S27. ^{1}H NMR Spectrum (400 MHz / $CDCl_3$ / 298 K) of **S4**·TFPB

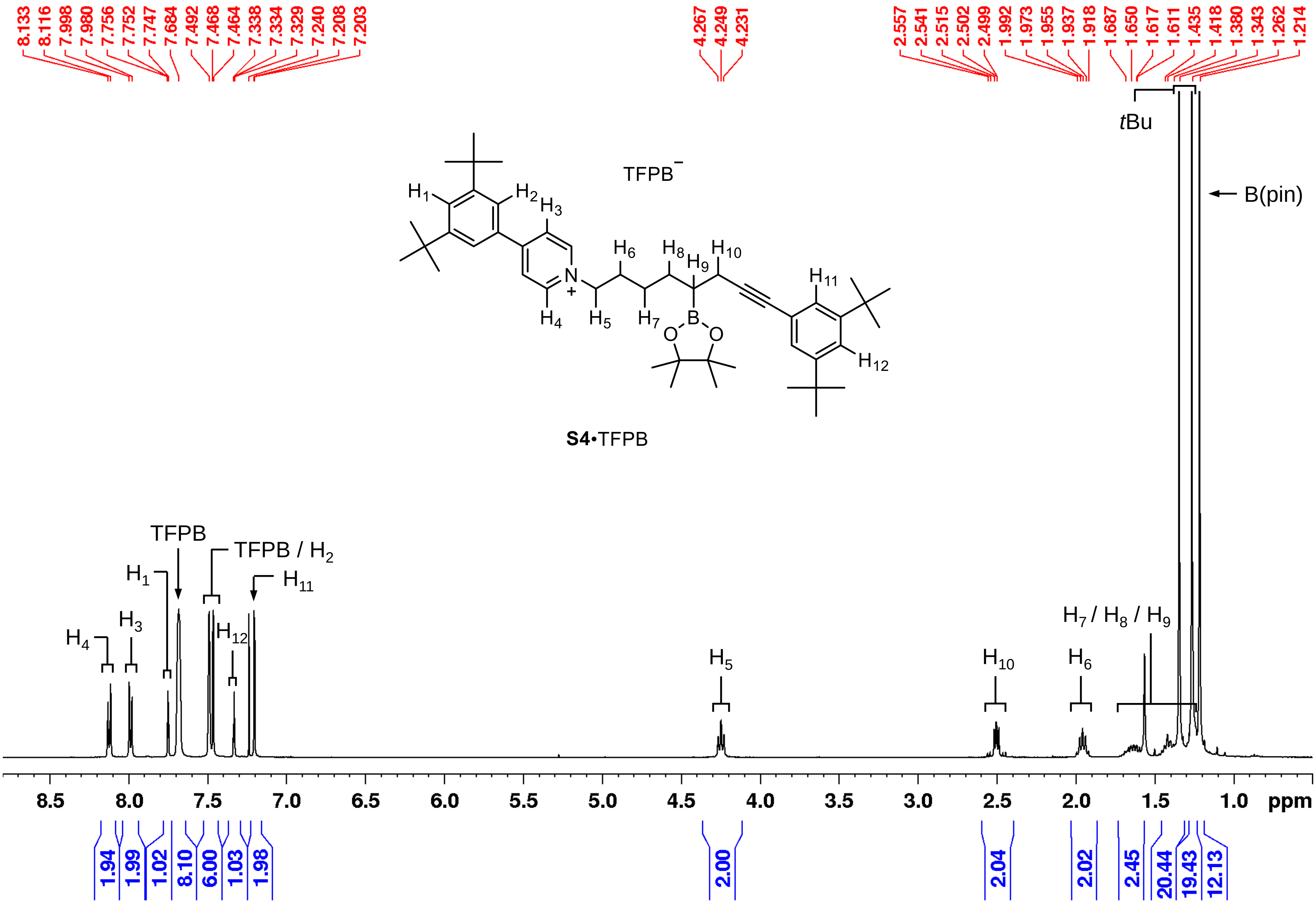

Figure S28. ^{13}C NMR Spectrum (100 MHz / $CDCl_3$ / 298 K) of **S4**·TFPB

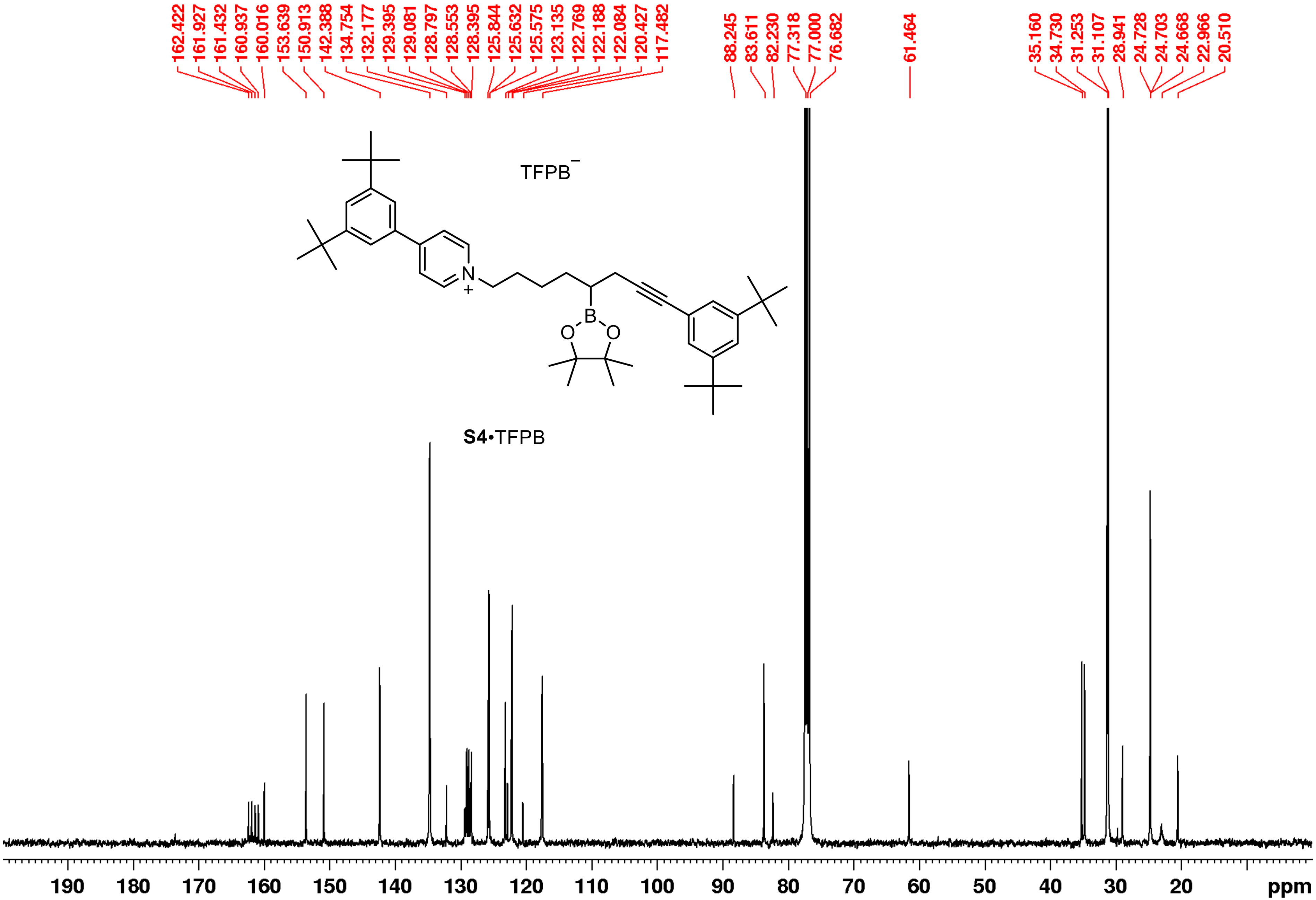

Figure S29. ^{1}H NMR Spectrum (400 MHz / $CDCl_3$ / 298 K) of **18**·TFPB

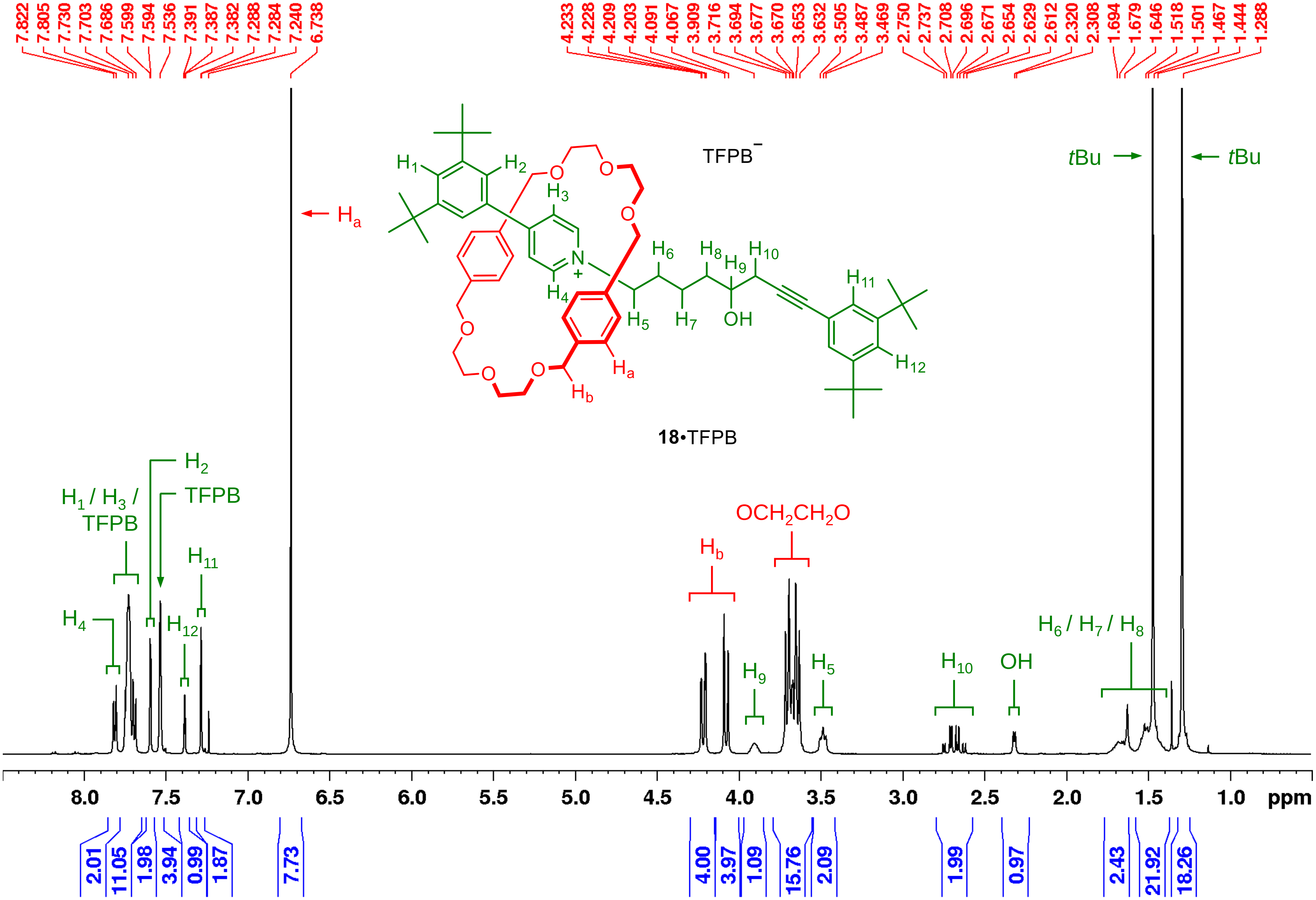

Figure S30. ^{13}C NMR Spectrum (100 MHz / $CDCl_3$ / 298 K) of **18**·TFPB

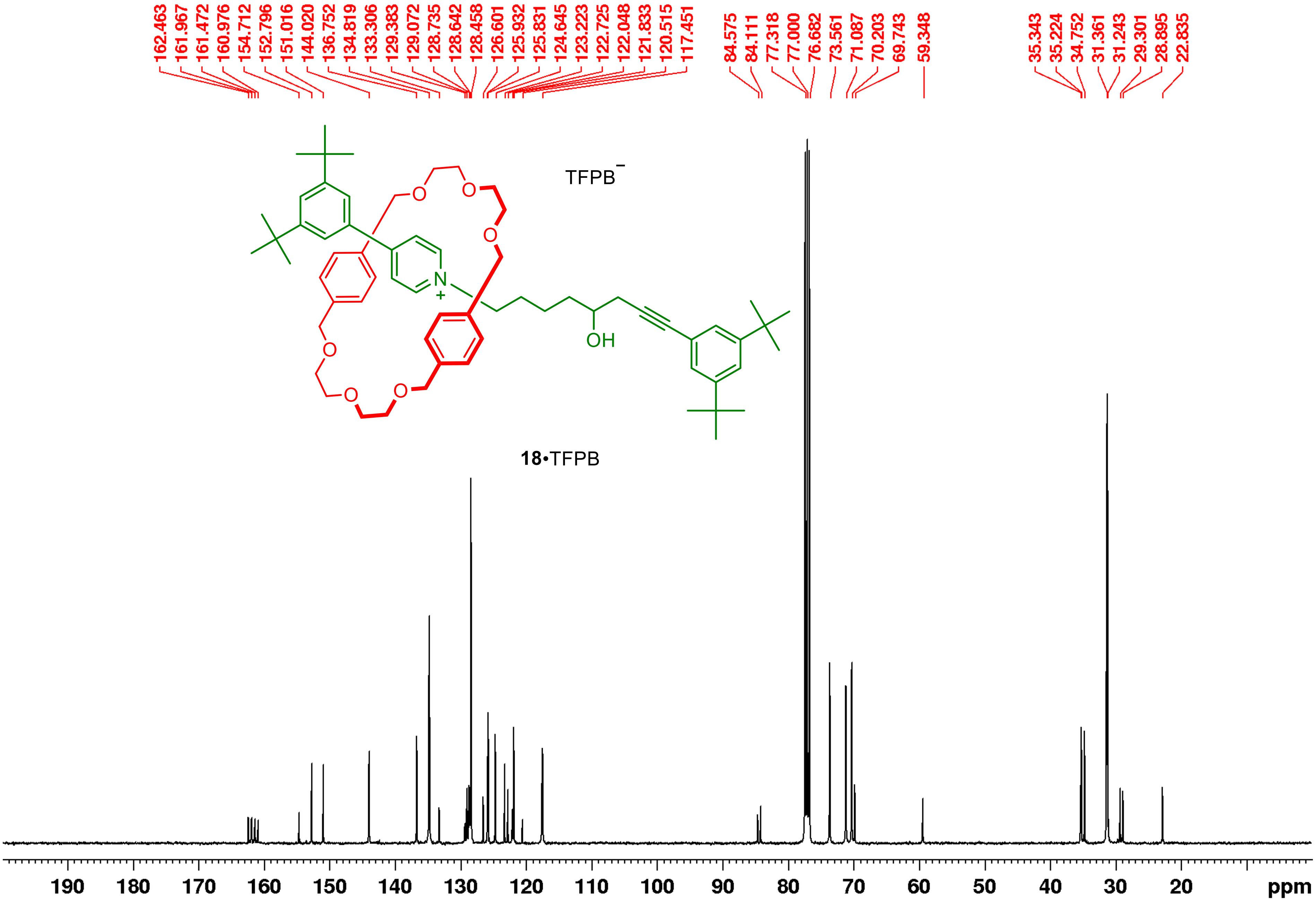

Figure S31. ^{1}H NMR Spectrum (500 MHz / $CDCl_3$ / 298 K) of **19**

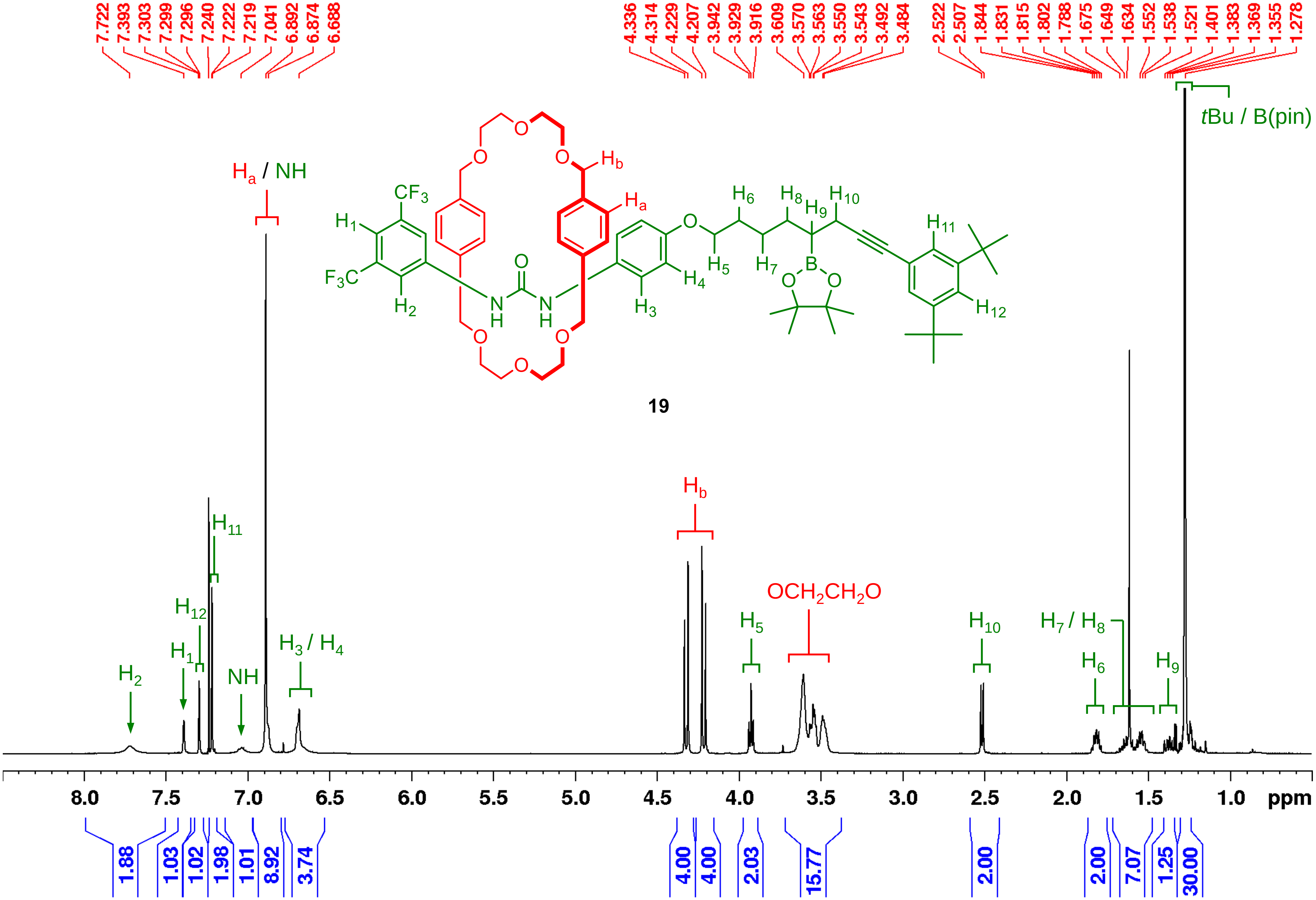

Figure S32. ^{13}C NMR Spectrum (125 MHz / $CDCl_3$ / 298 K) of **19**

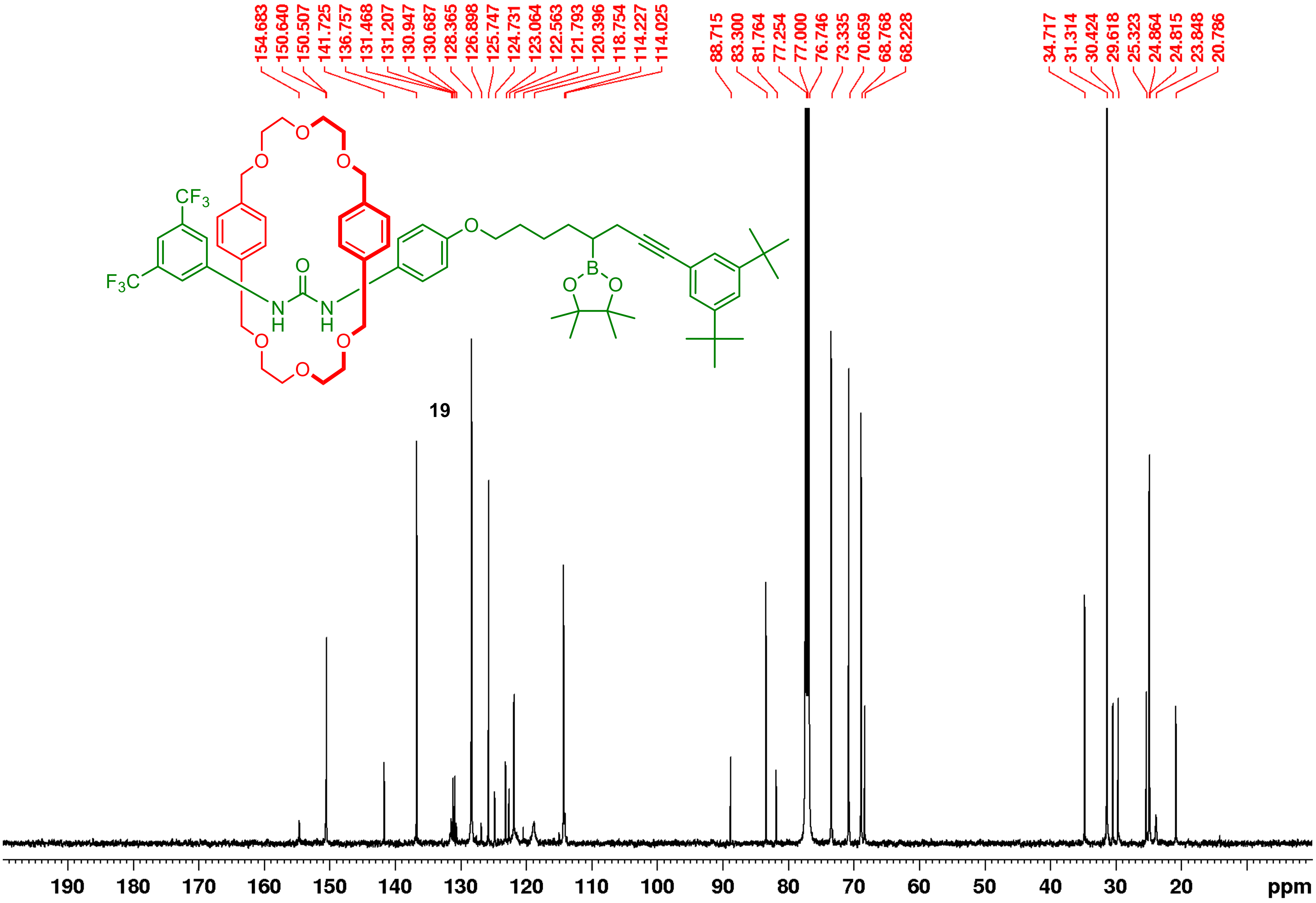

Figure S33. ^{1}H NMR Spectrum (400 MHz / $CDCl_3$ / 298 K) of **S5**

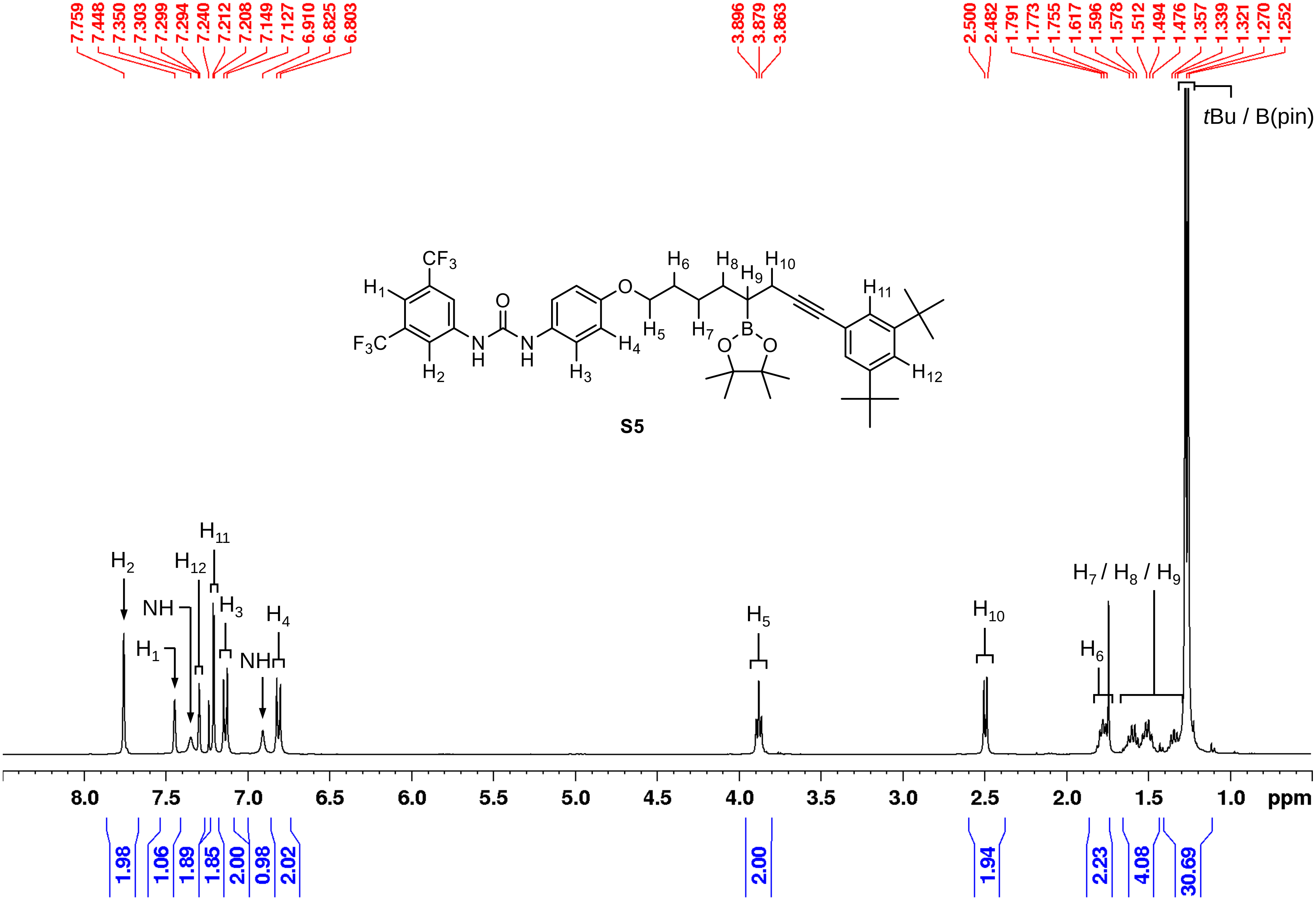

Figure S34. ^{13}C NMR Spectrum (100 MHz / $CDCl_3$ / 298 K) of **S5**

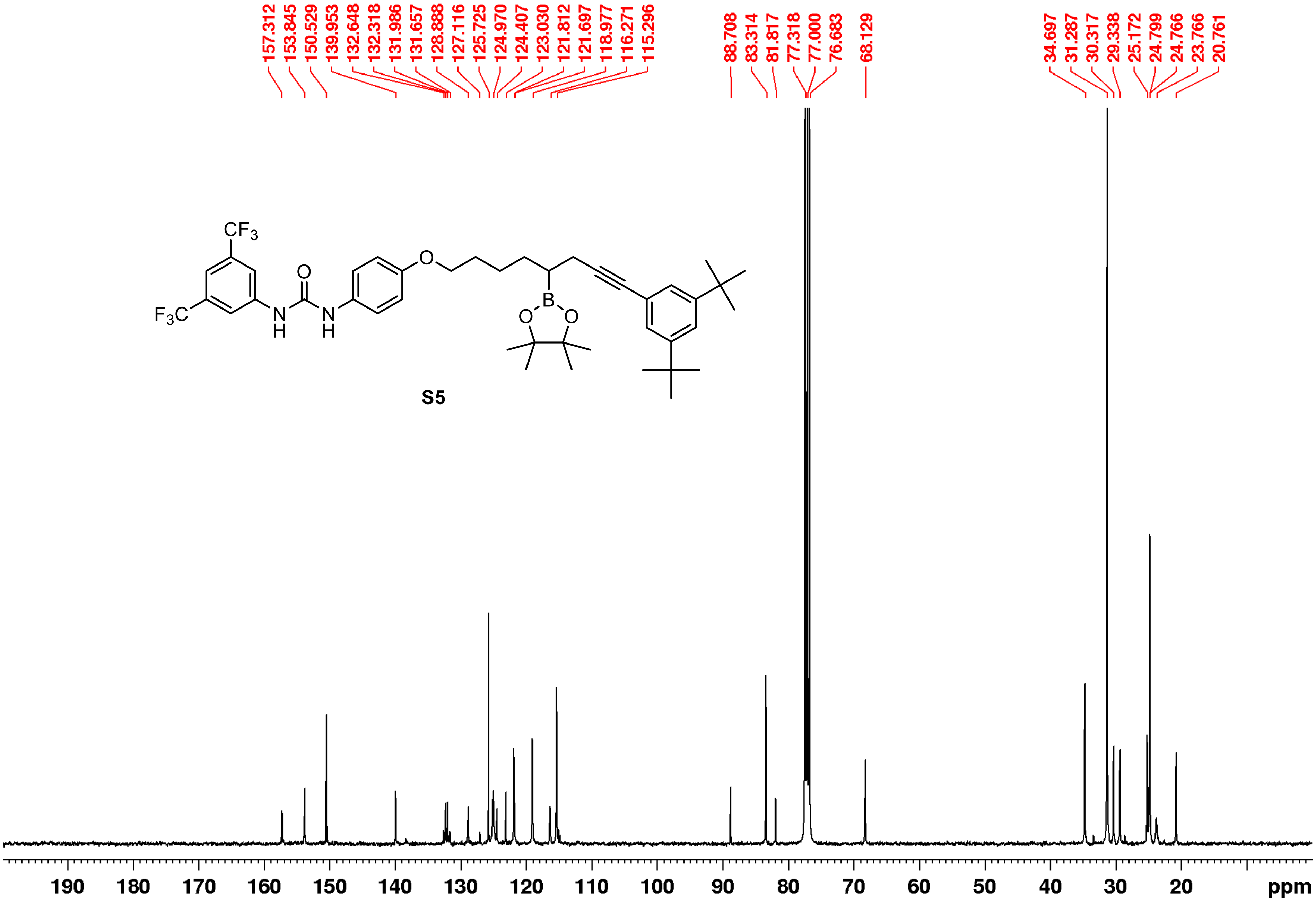

Figure S35. ^{1}H NMR Spectrum (400 MHz / $CDCl_3$ / 298 K) of **20**

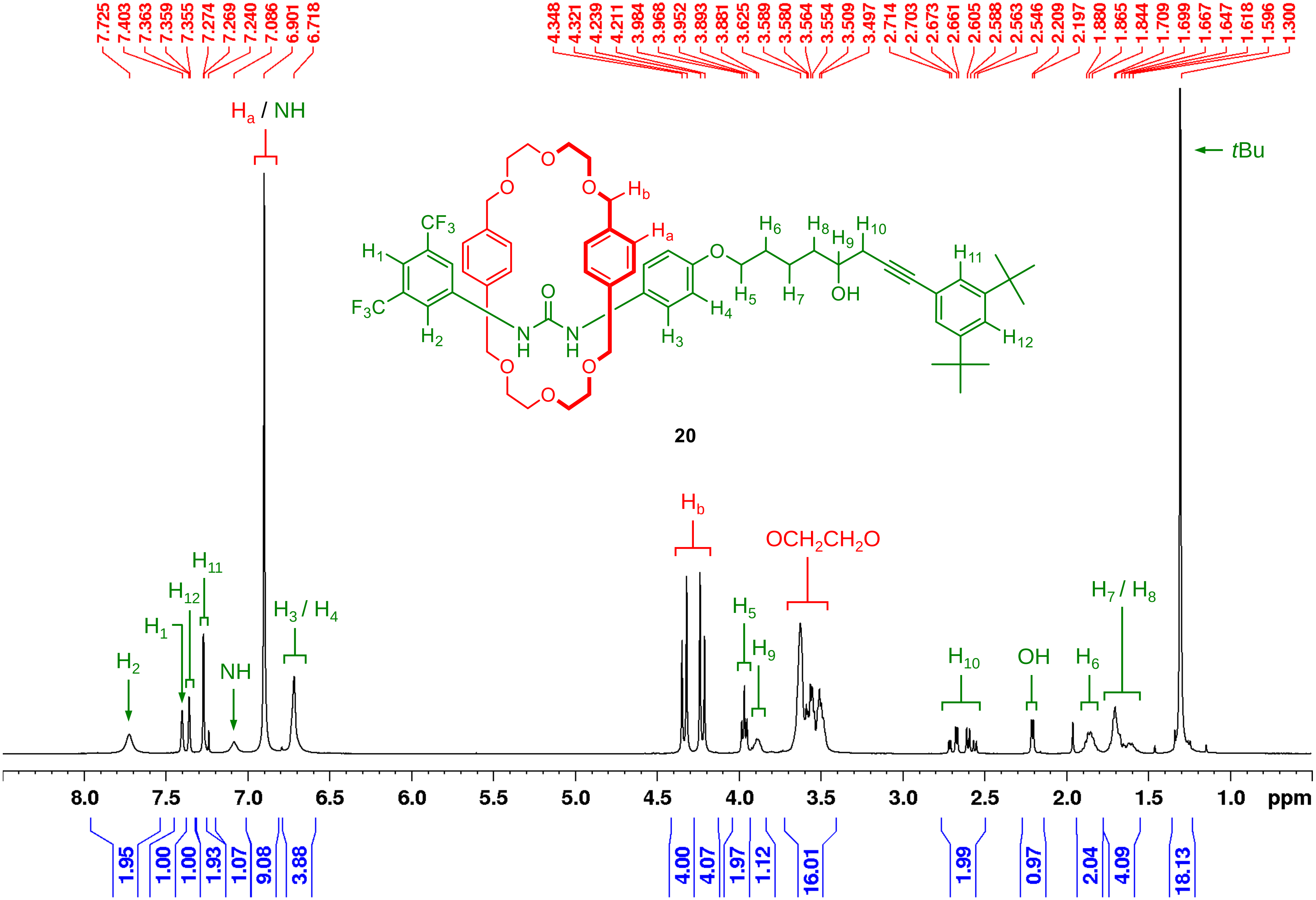

Figure S36. ^{13}C NMR Spectrum (100 MHz / $CDCl_3$ / 298 K) of **20**

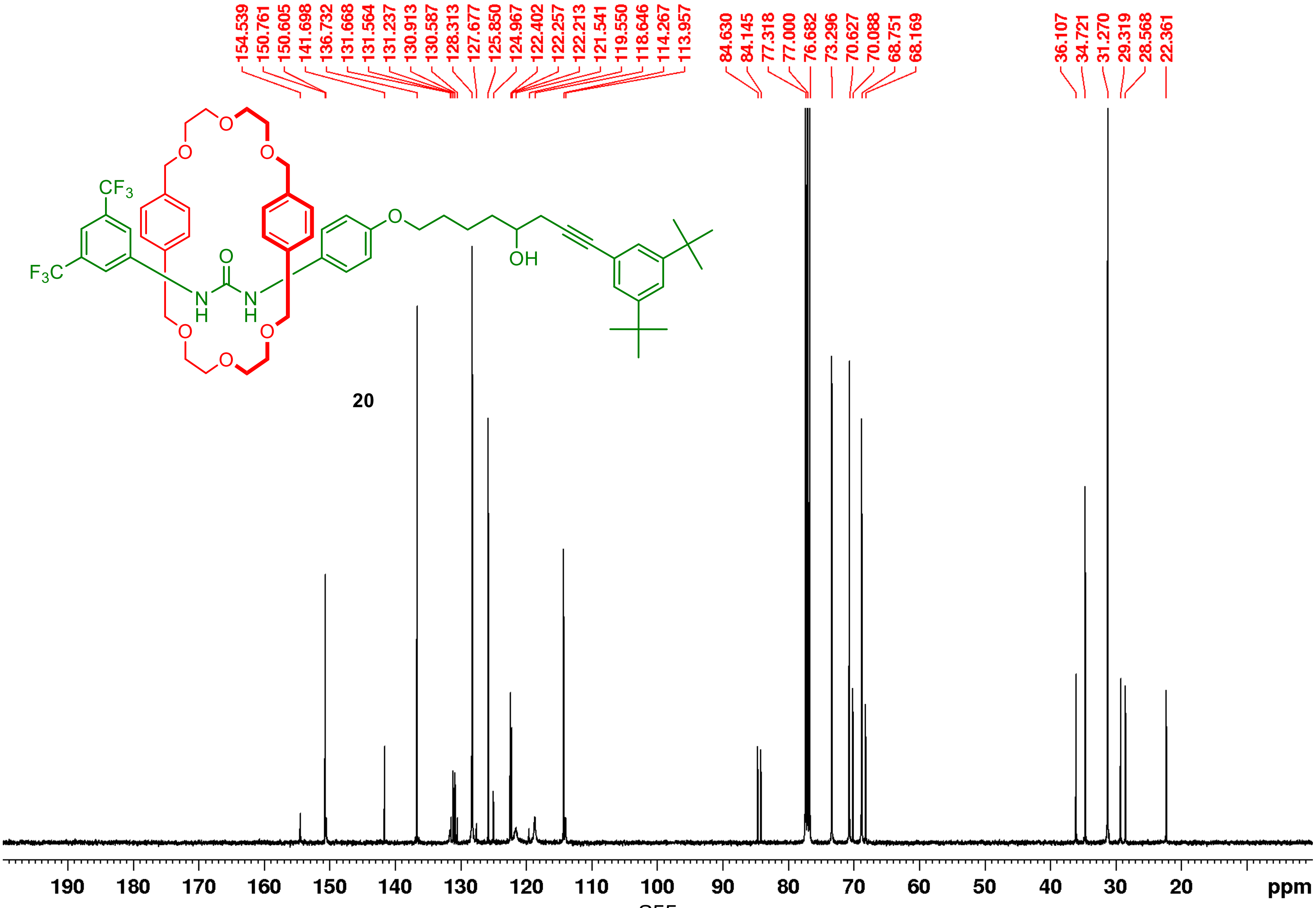

Figure S37. 2D COSY Spectrum (400 MHz / CD_2Cl_2 / 298 K) of **7**·TFPB

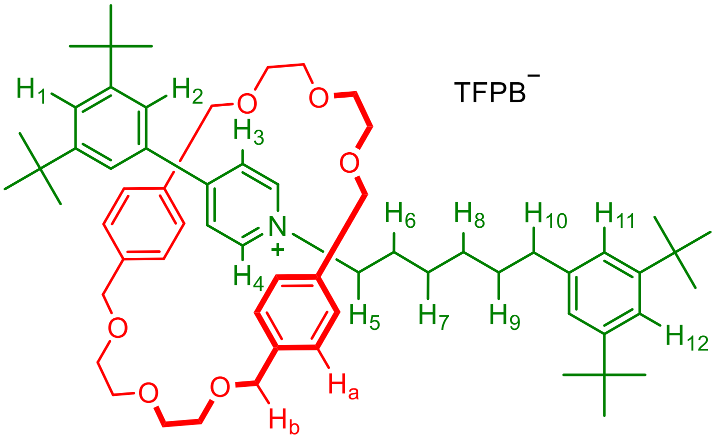

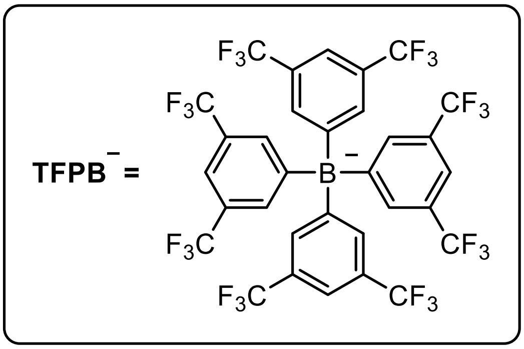

7•TFPB

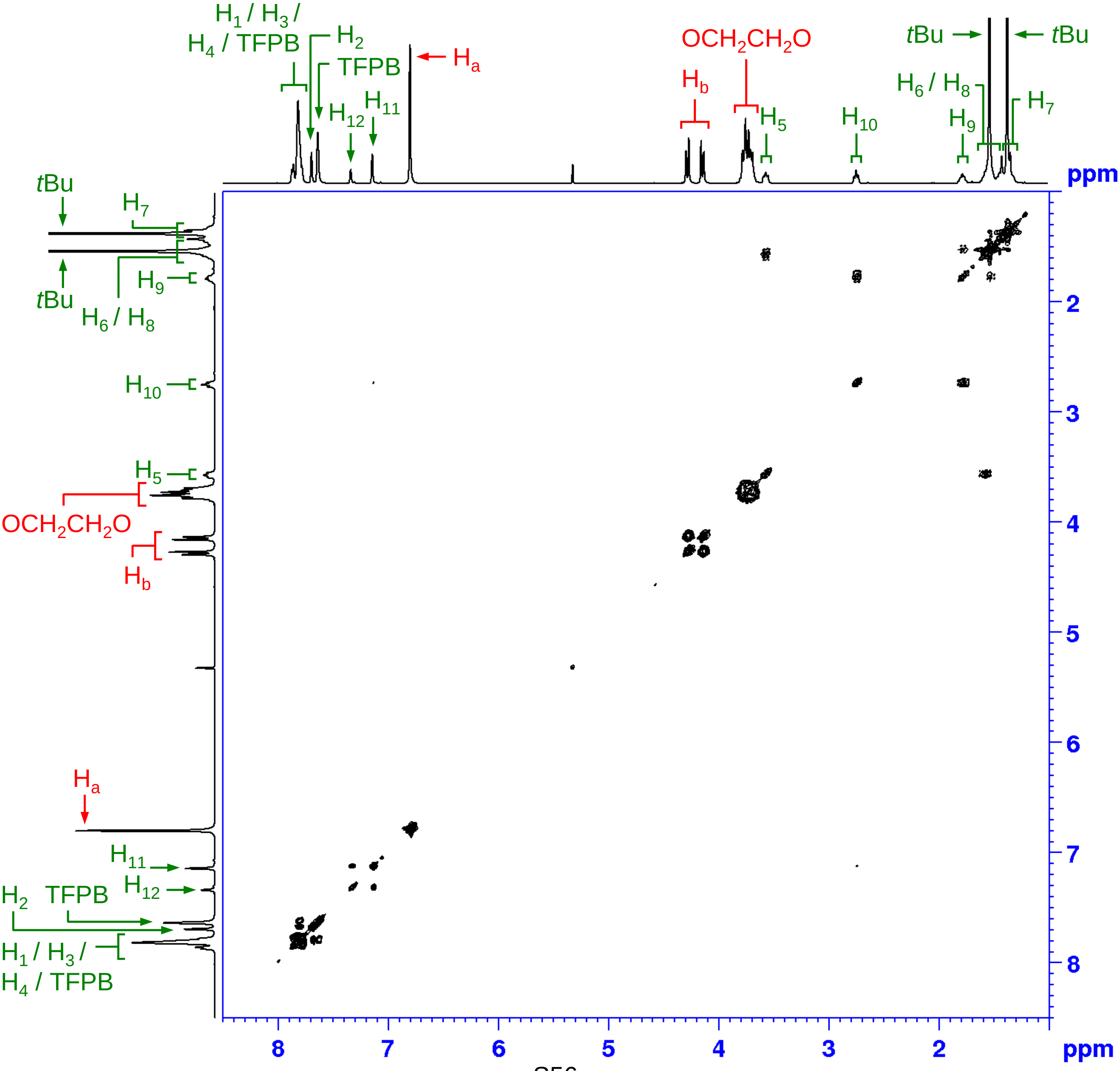

Figure S38. 2D ROESY Spectrum (400 MHz / CD_2Cl_2 / 298 K) of **7**·TFPB

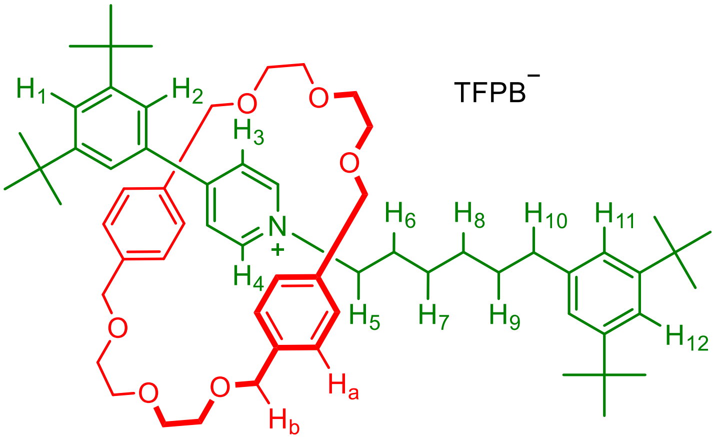

7•TFPB

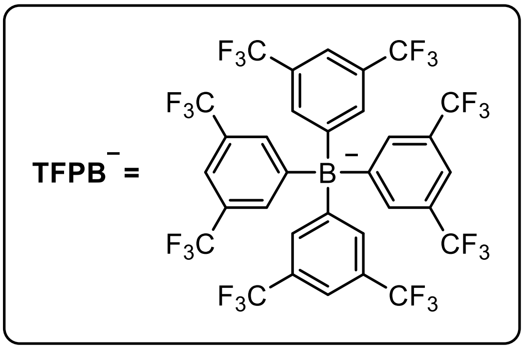

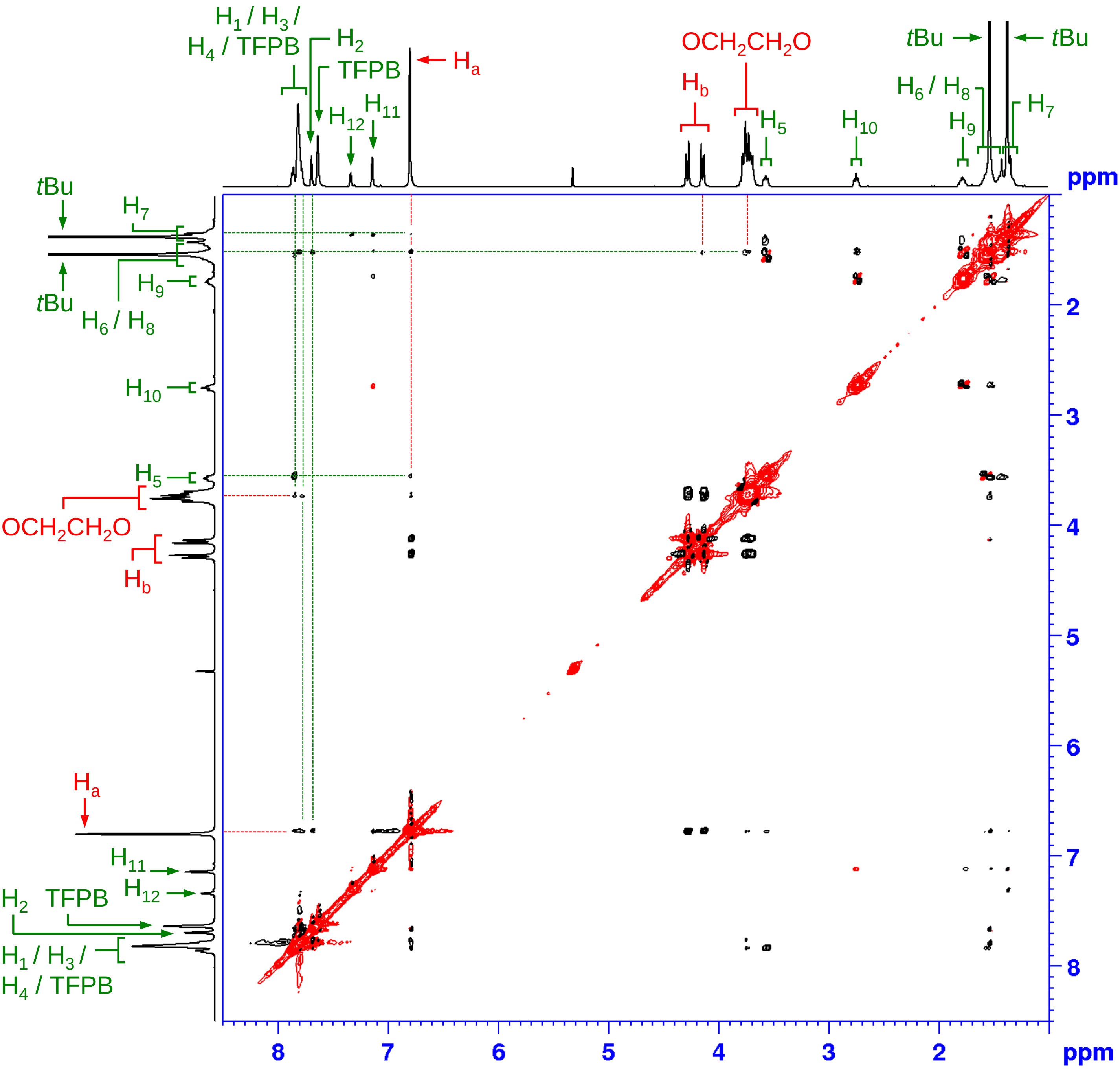

Figure S39. 2D COSY Spectrum (400 MHz / CD_2Cl_2 / 298 K) of **9**

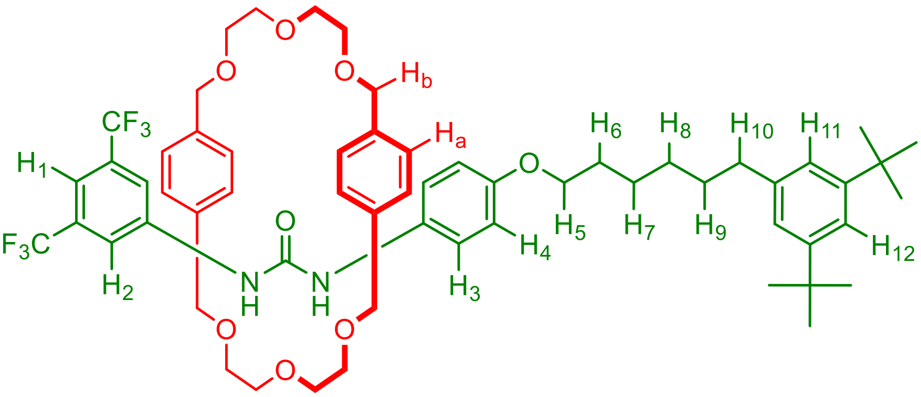

9

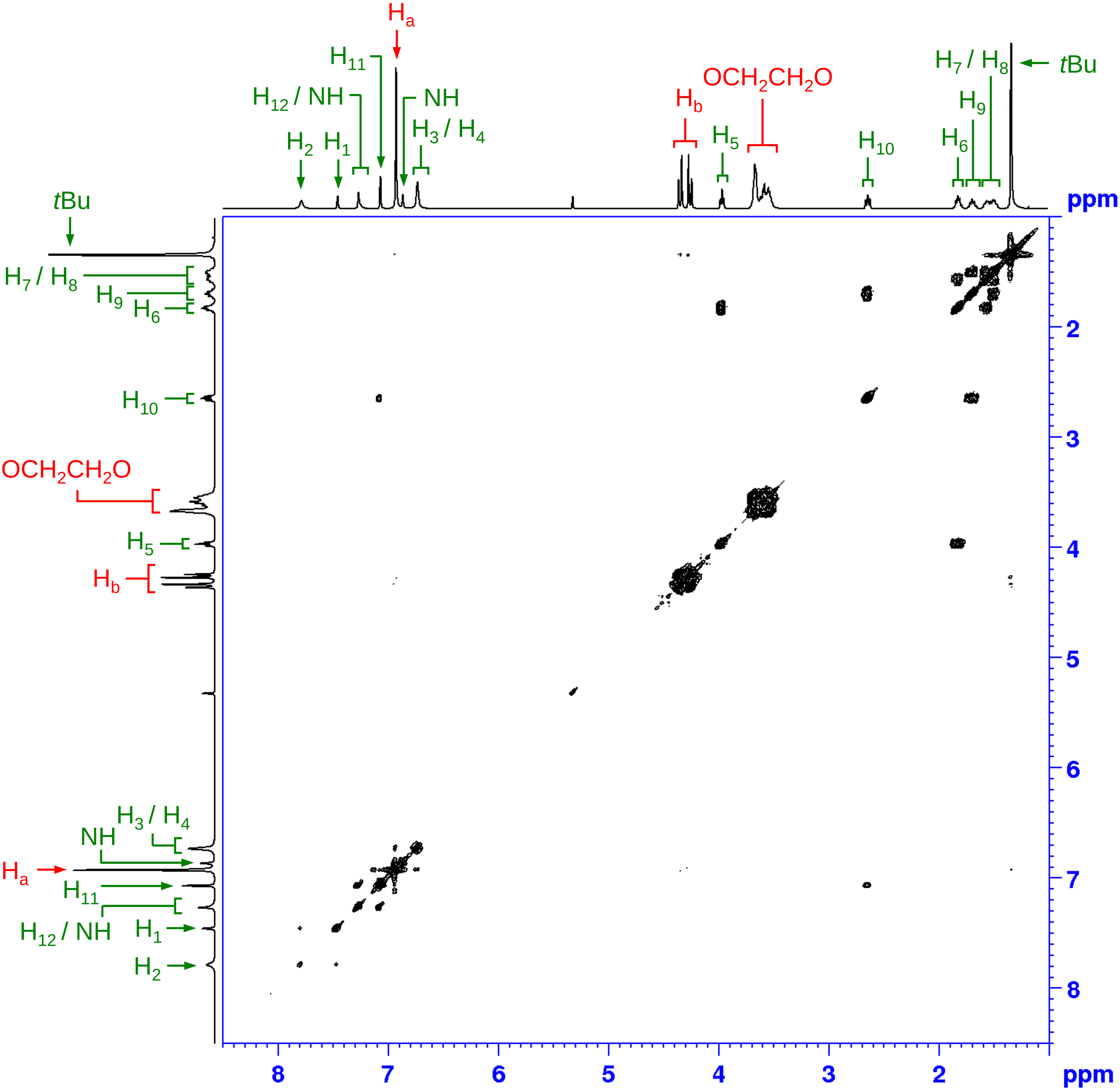

Figure S40. 2D ROESY Spectrum (400 MHz / CD_2Cl_2 / 298 K) of **9**

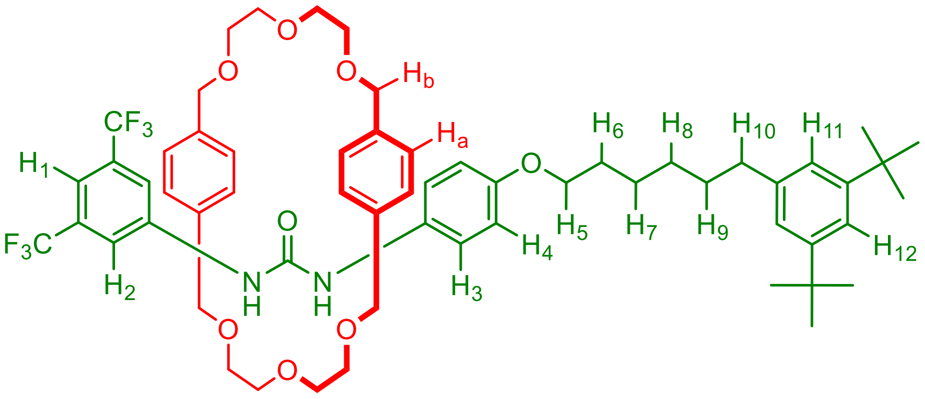

9

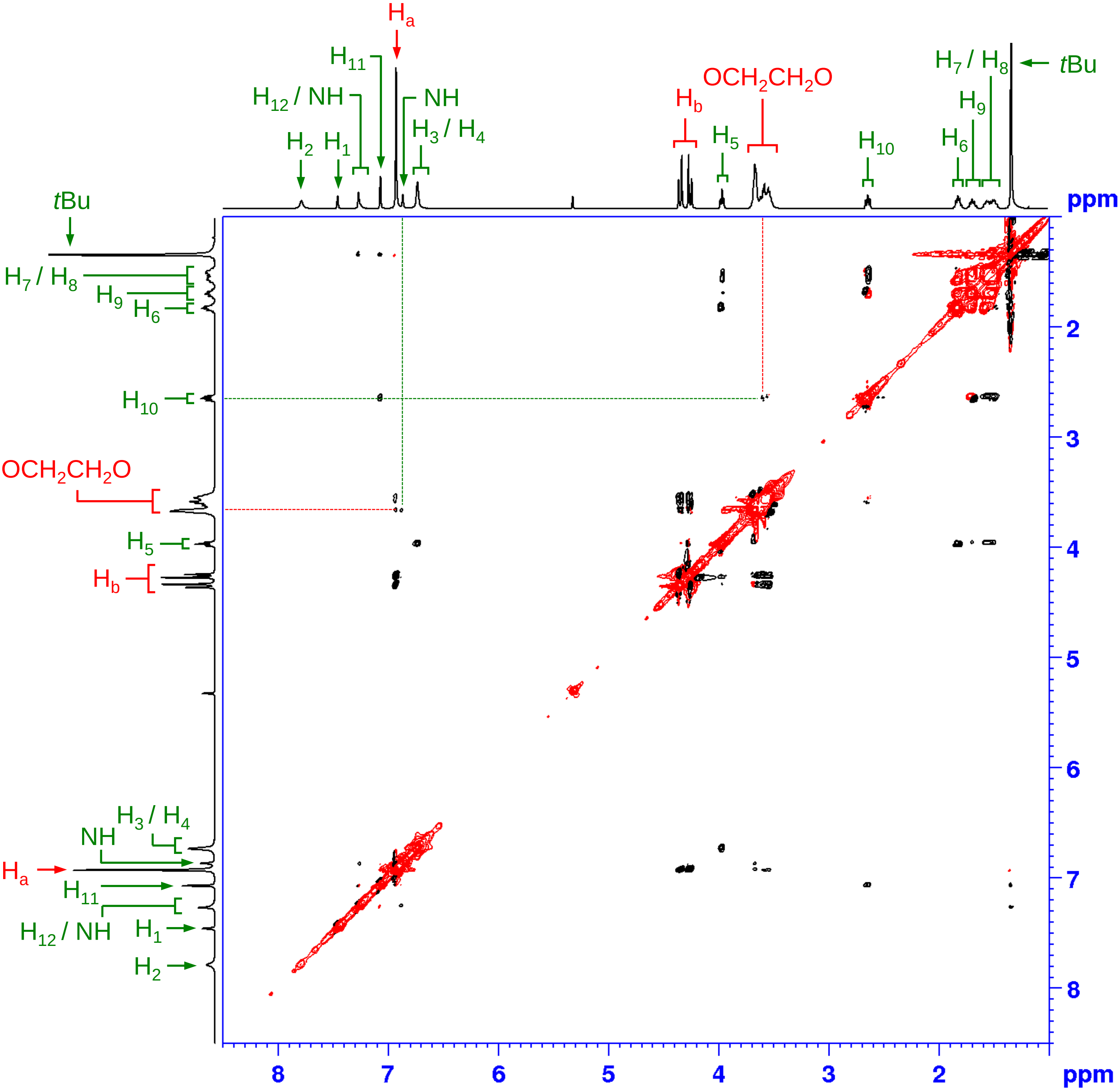

Figure S41. 2D COSY Spectrum (800 MHz / $CDCl_3$ / 298 K) of **12**·TFPB

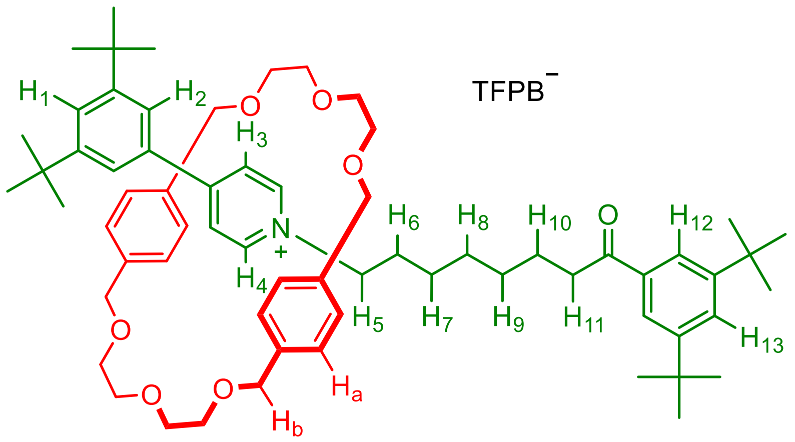

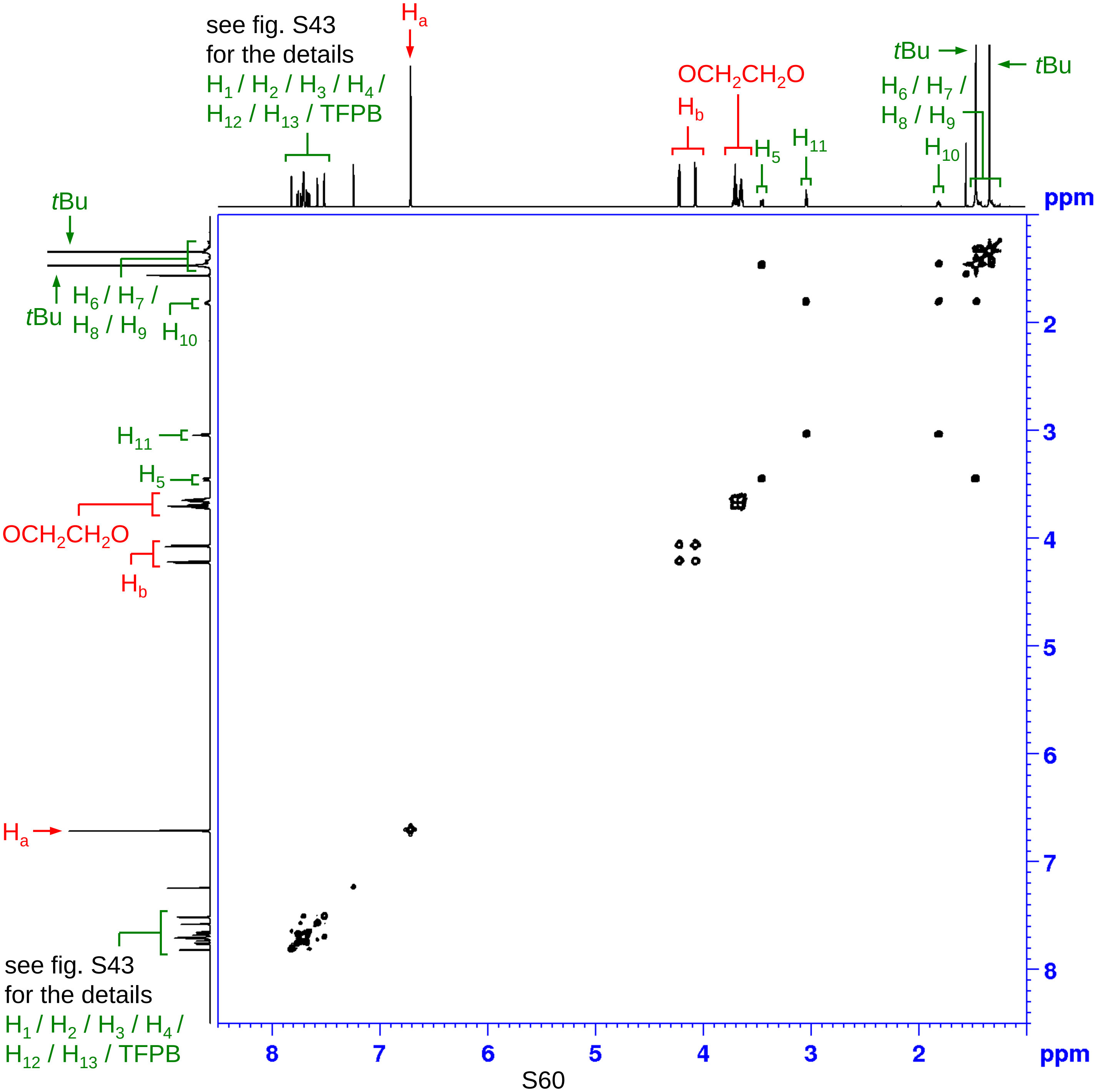

Figure S42. 2D ROESY Spectrum (800 MHz / $CDCl_3$ / 298 K) of **12**·TFPB

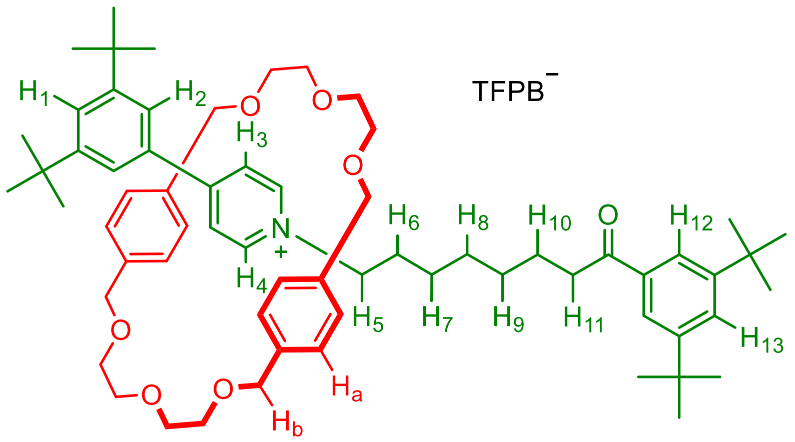

12•TFPB

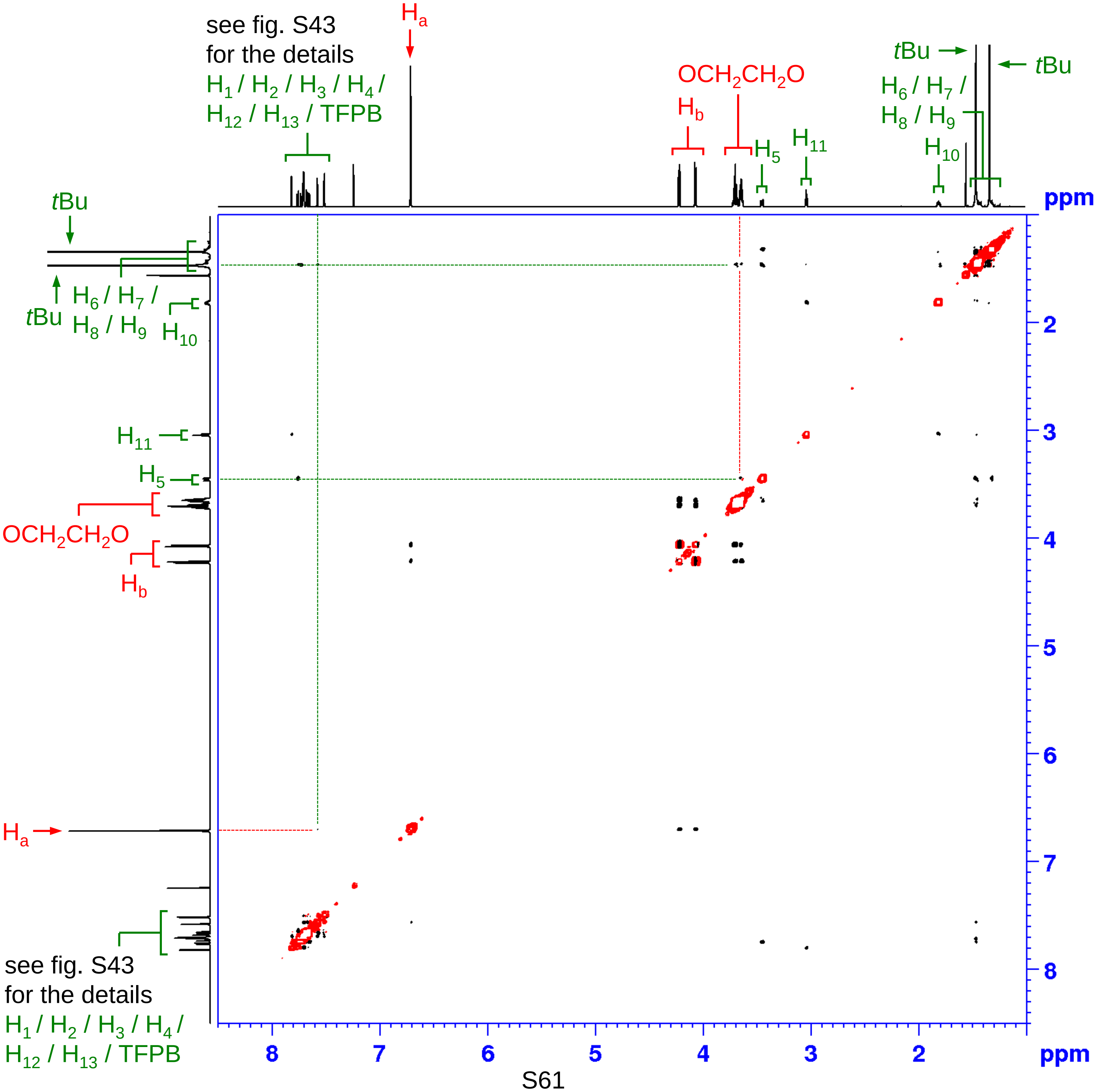

Figure S43. Partial COSY and ROESY Spectrum (800 MHz / $CDCl_3$ / 298 K) of **12**·TFPB

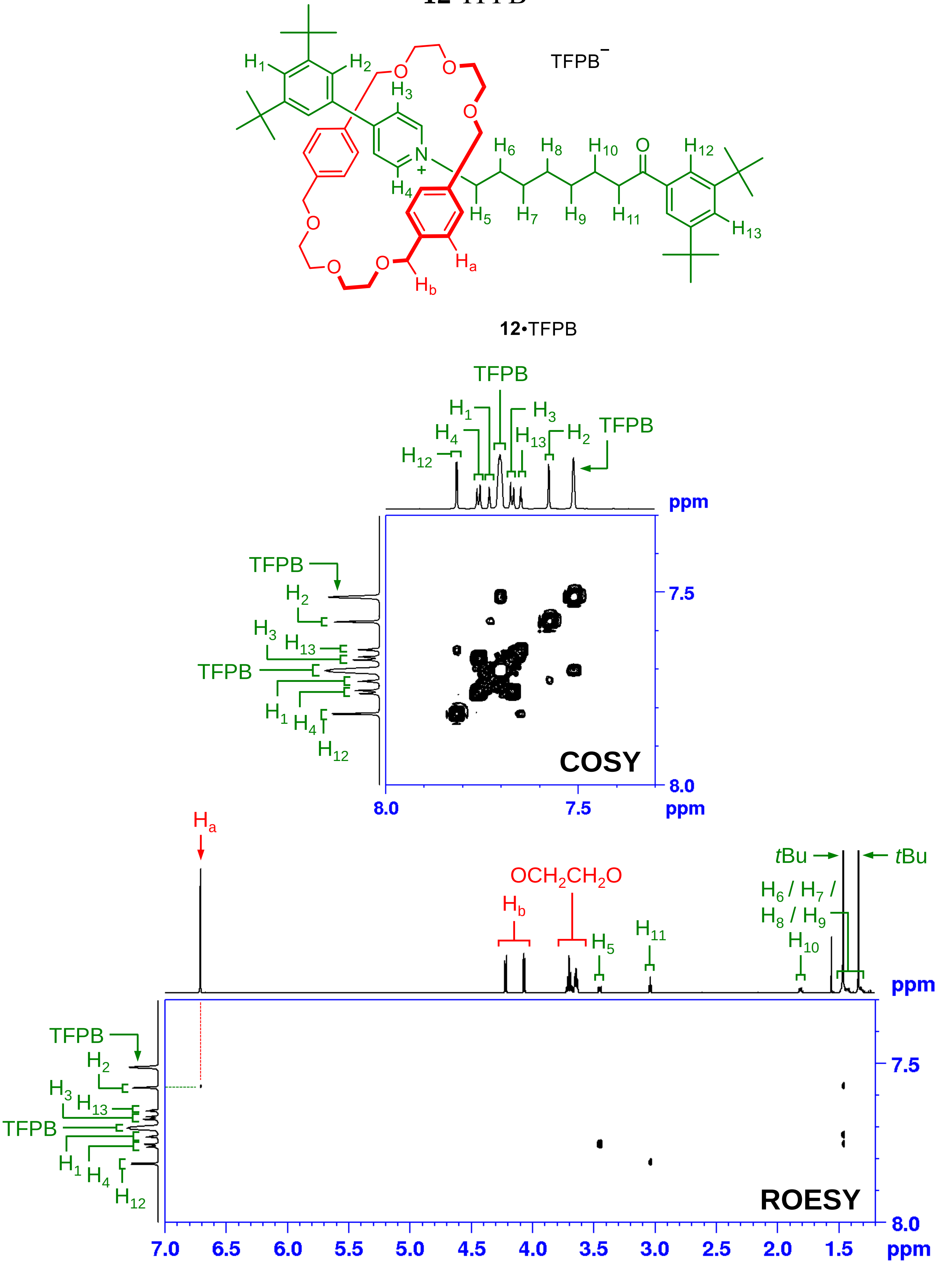

Figure S44. 2D COSY Spectrum (500 MHz / CD_2Cl_2 / 298 K) of **13**

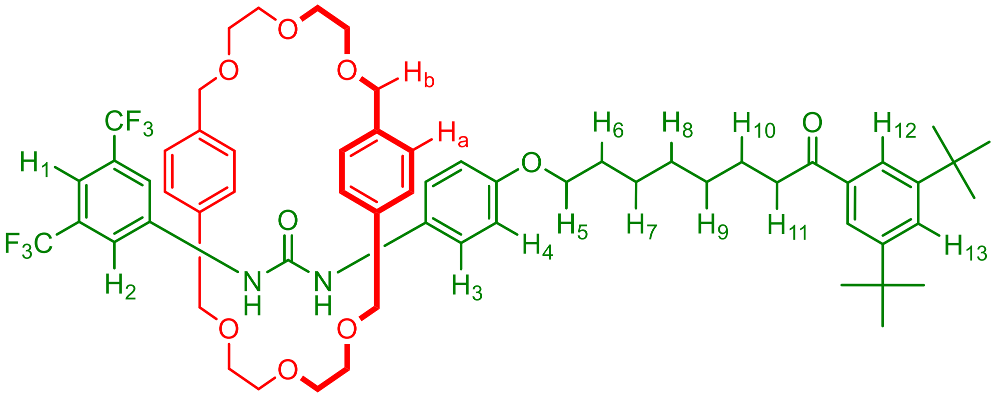

13

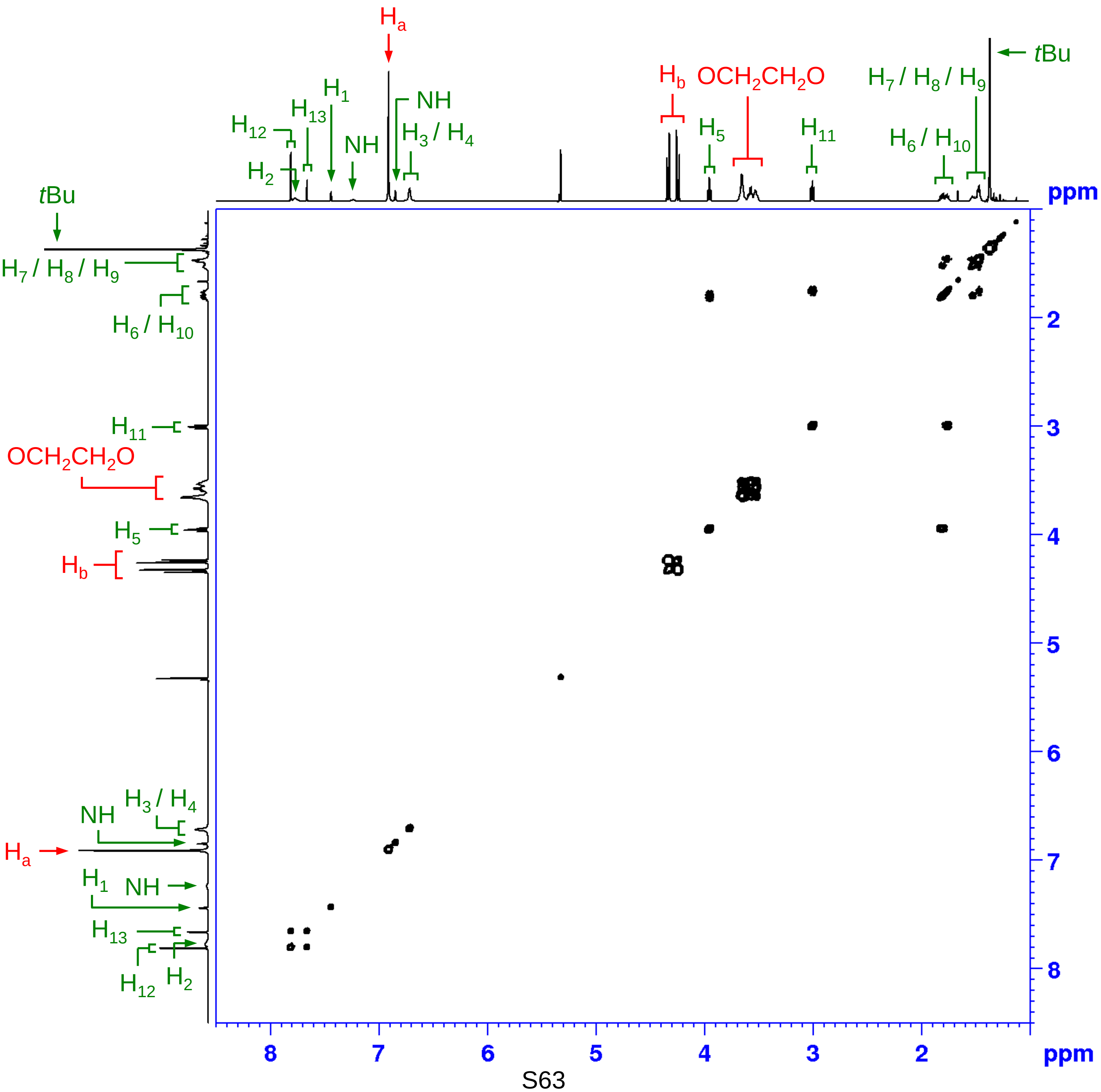

Figure S45. 2D ROESY Spectrum (500 MHz / CD_2Cl_2 / 298 K) of **13**

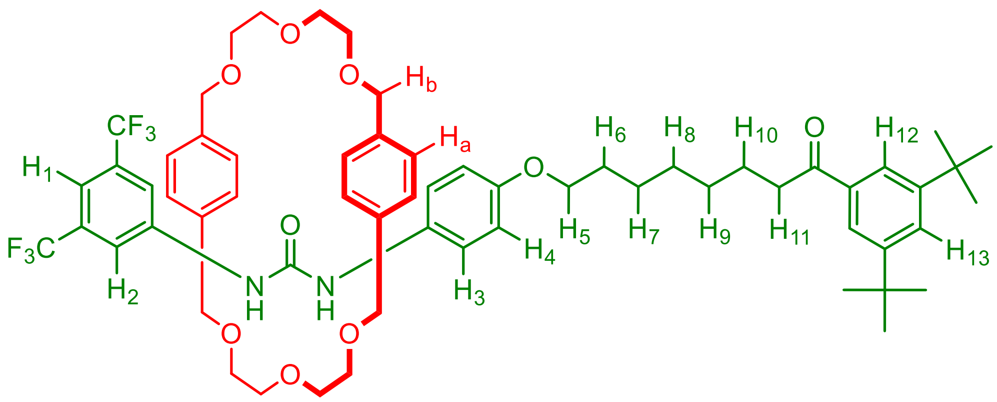

13

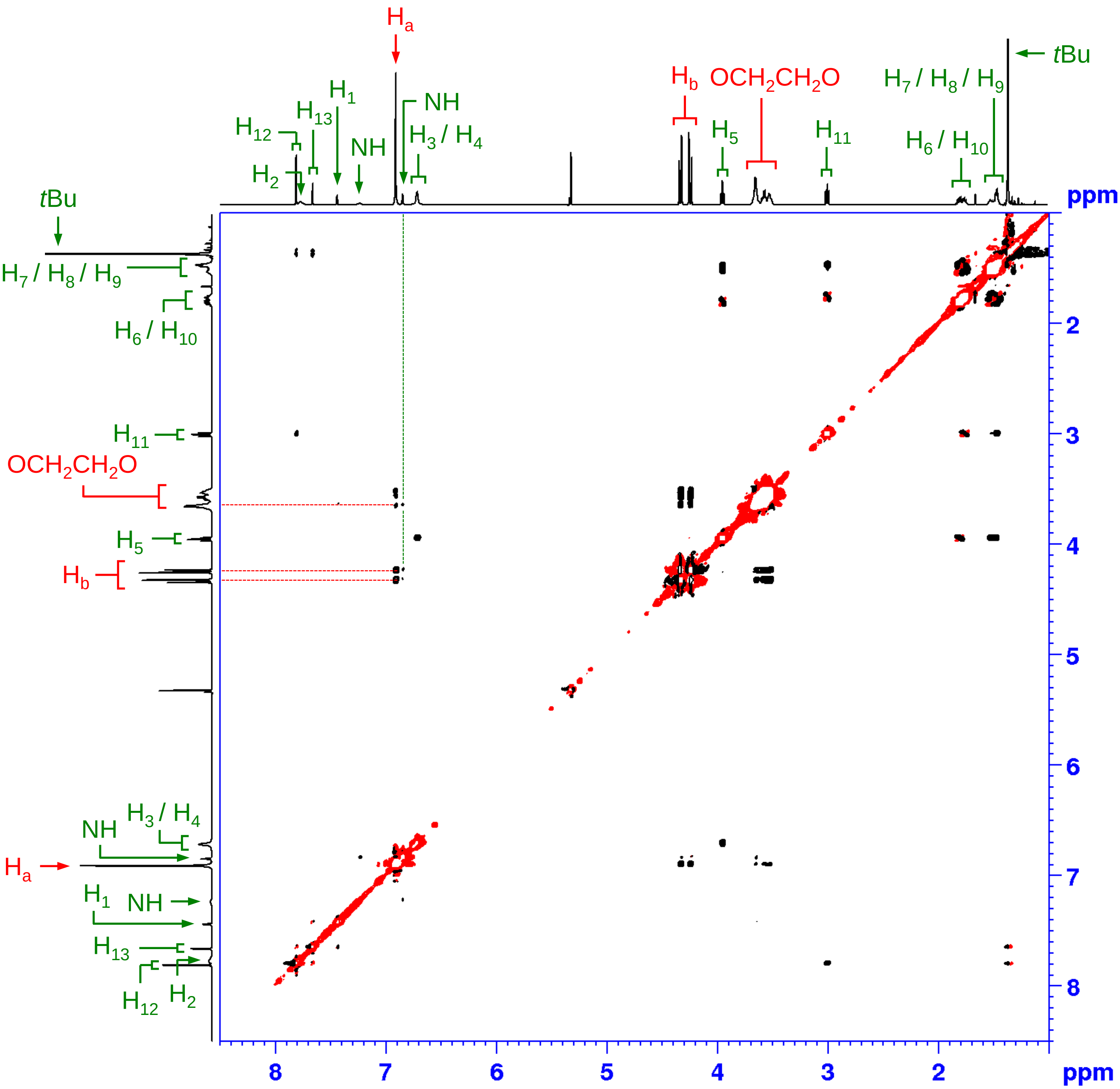

Figure S46. 2D COSY Spectrum (400 MHz / CD_2Cl_2 / 298 K) of **S1**·TFPB

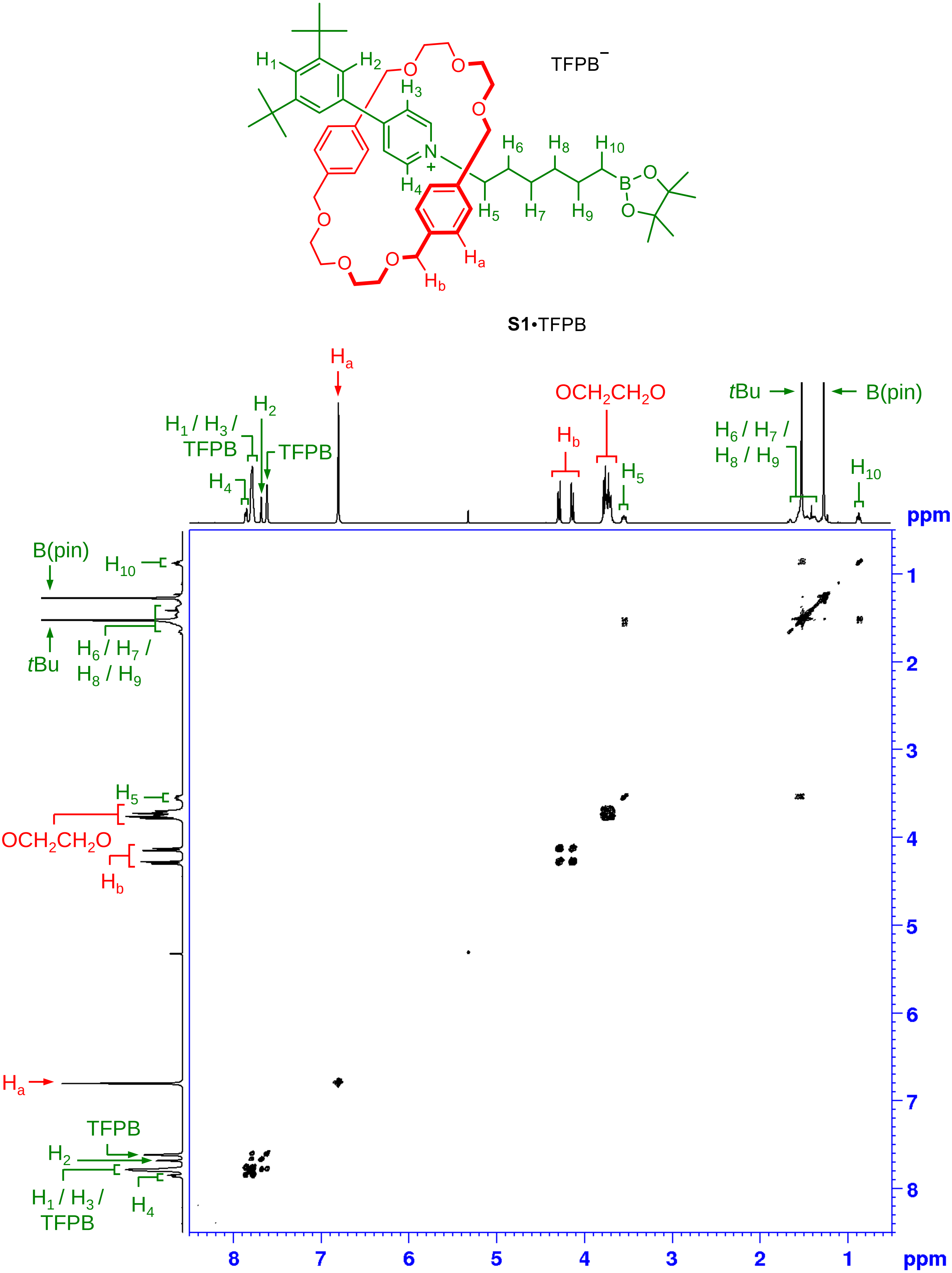

Figure S47. 2D ROESY Spectrum (400 MHz / CD_2Cl_2 / 298 K) of **S1**·TFPB

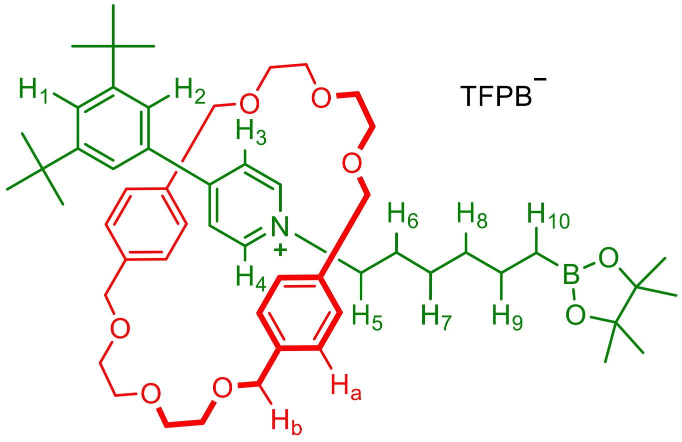

S1•TFPB

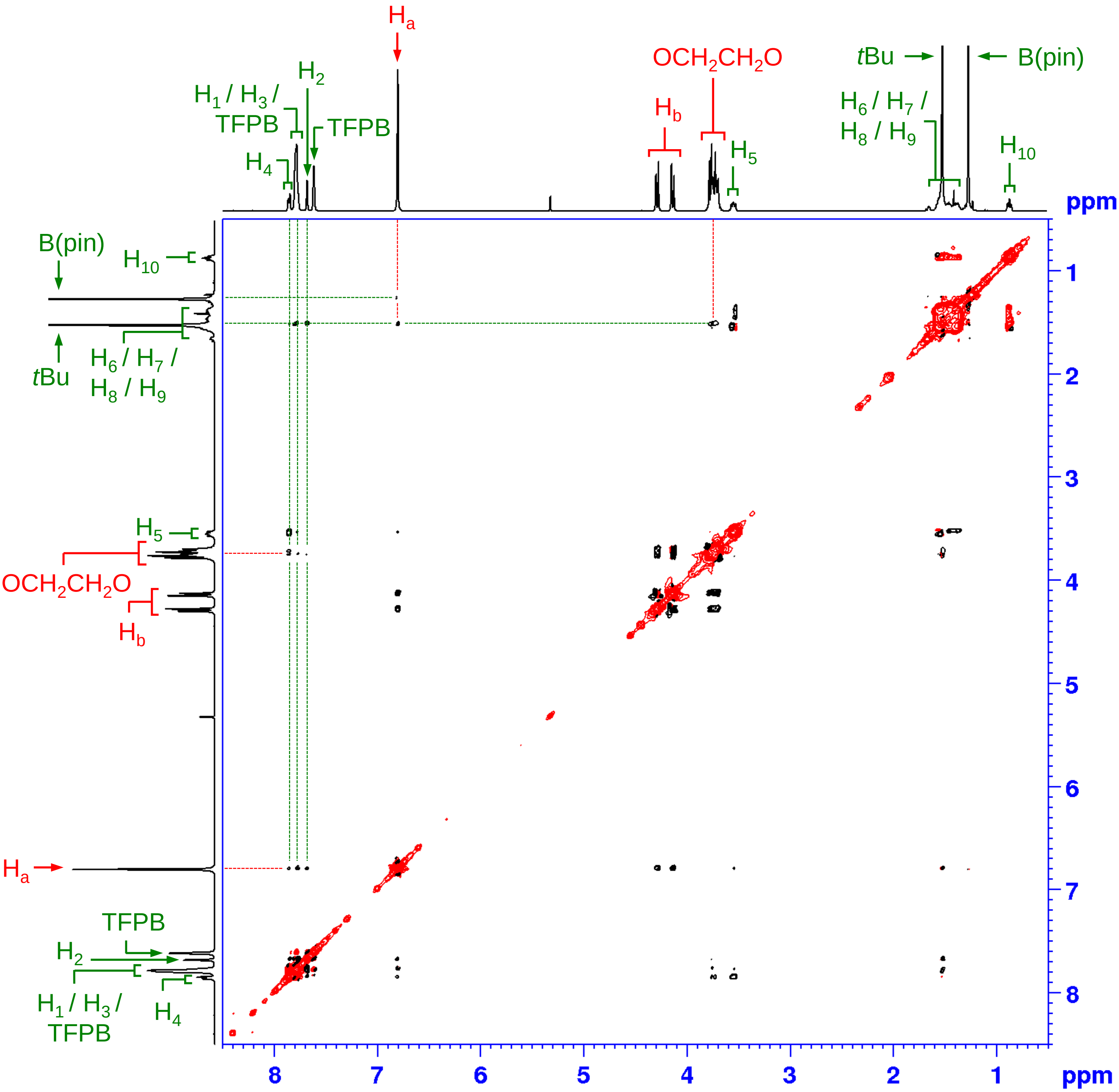

Figure S48. 2D COSY Spectrum (400 MHz / CD_2Cl_2 / 298 K) of **14**·TFPB

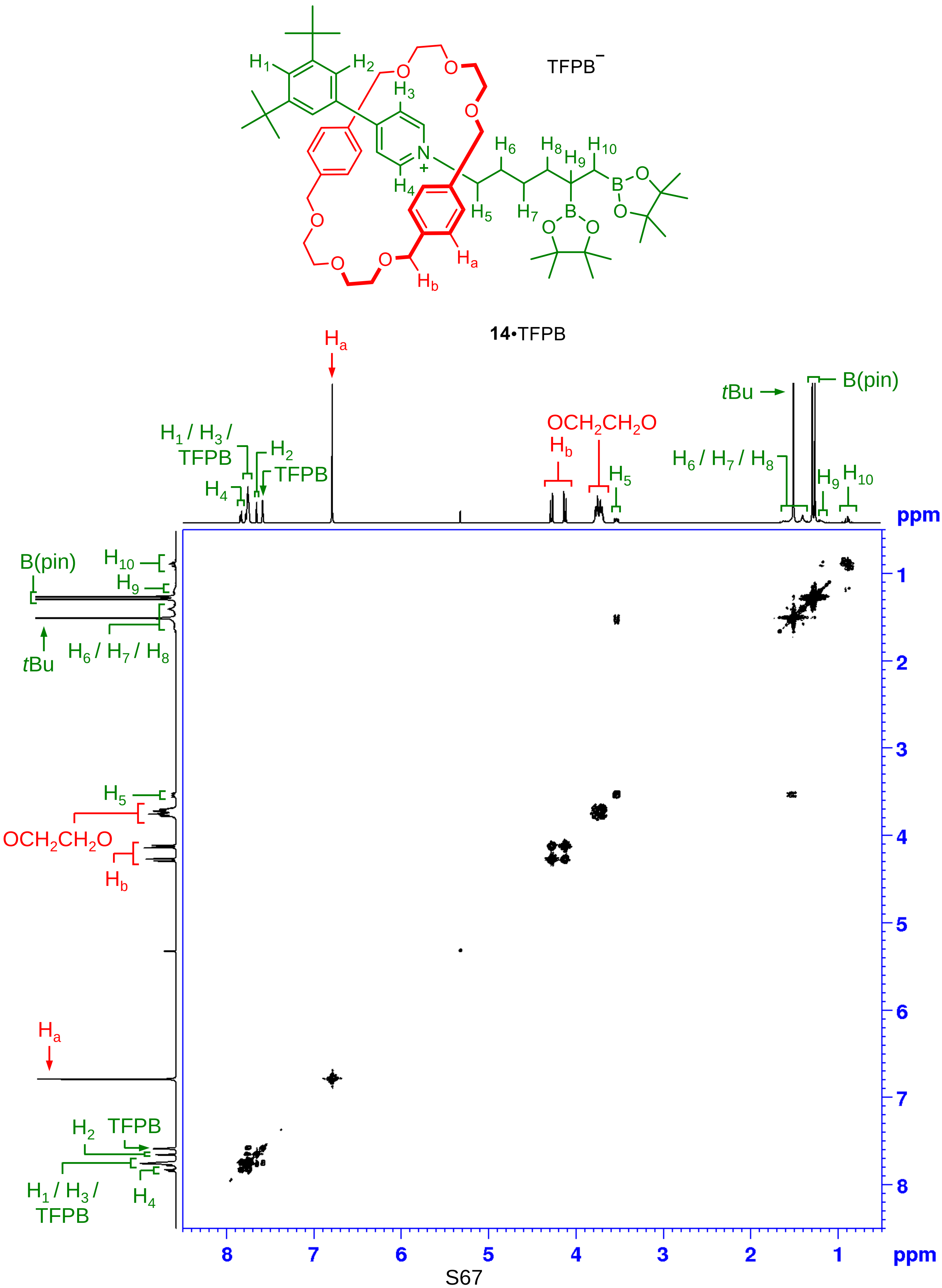

Figure S49. 2D ROESY Spectrum (400 MHz / CD_2Cl_2 / 298 K) of **14**·TFPB

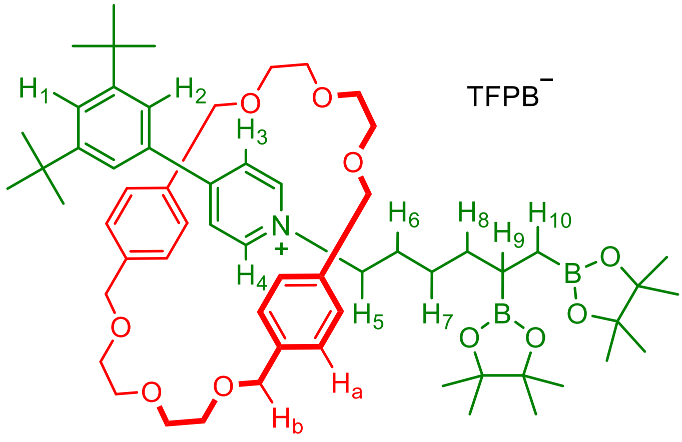

14•TFPB

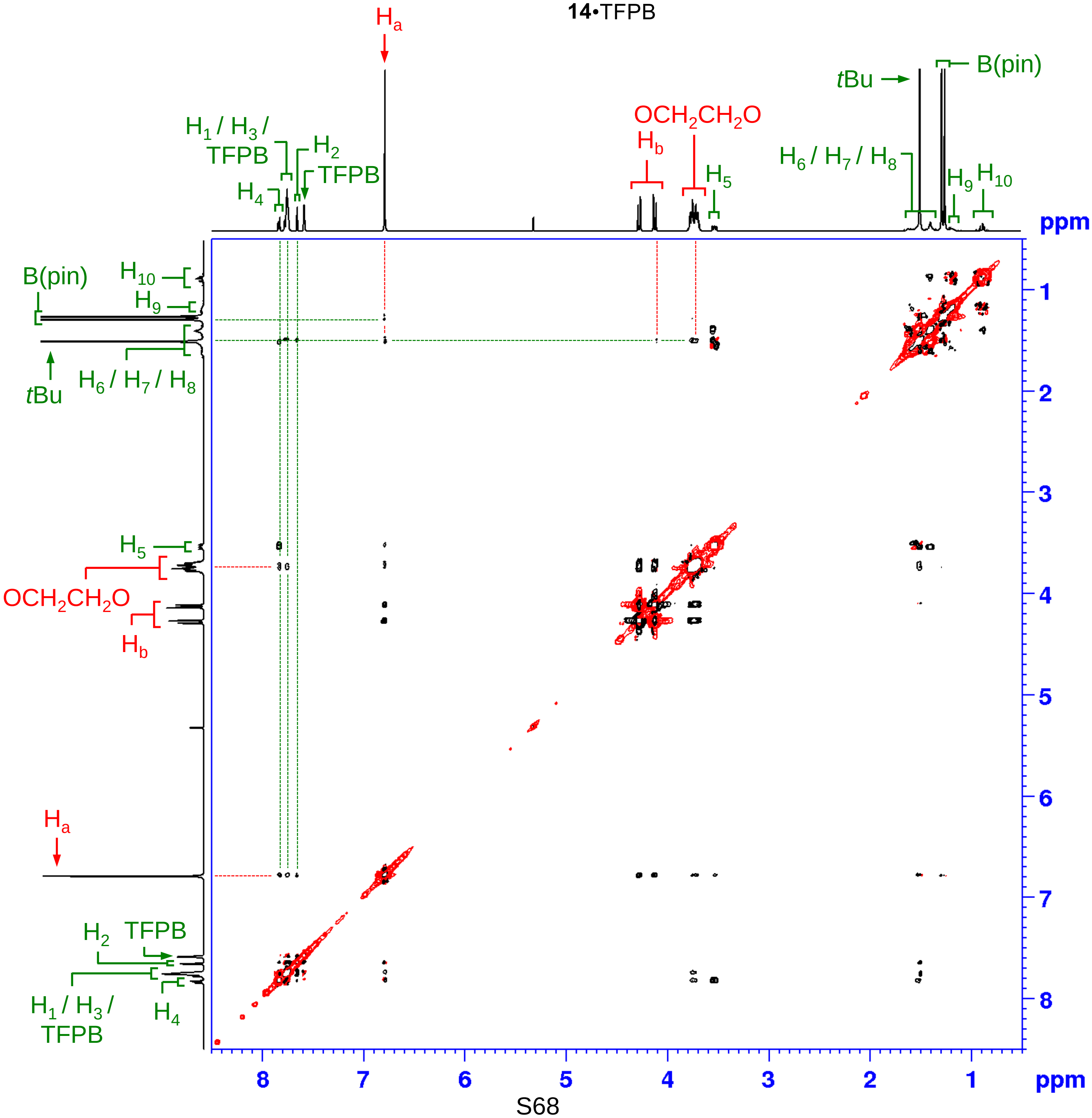

Figure S50. 2D COSY Spectrum (800 MHz / CD_2Cl_2 / 298 K) of **15**

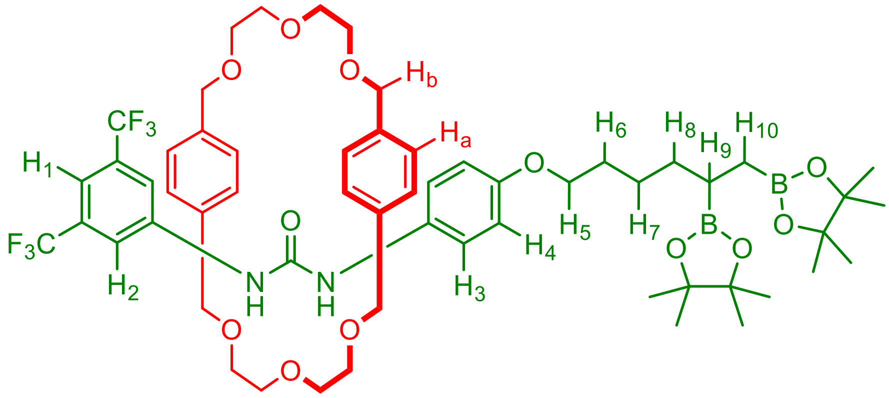

15

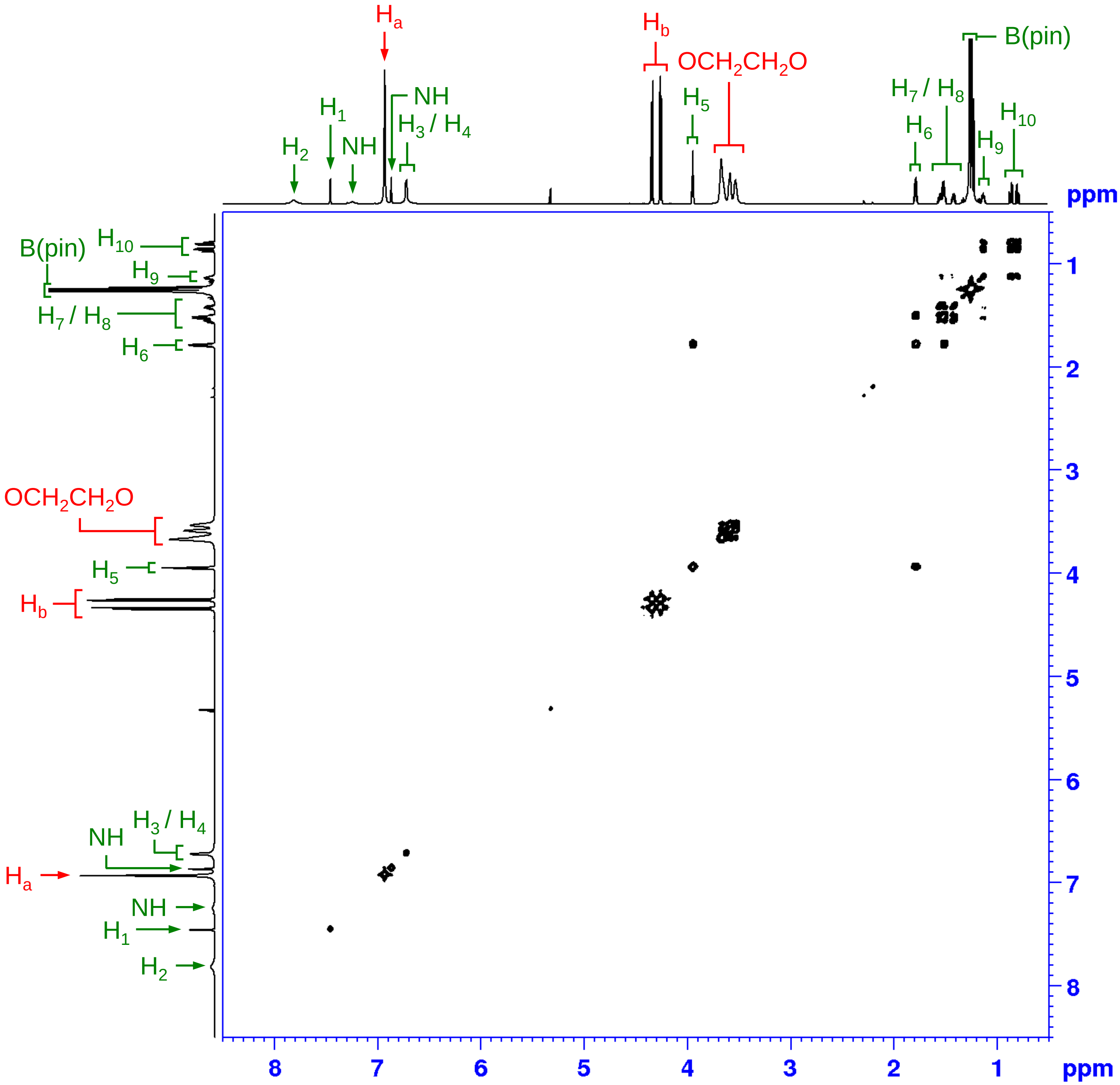

Figure S51. 2D ROESY Spectrum (800 MHz / CD_2Cl_2 / 298 K) of **15**

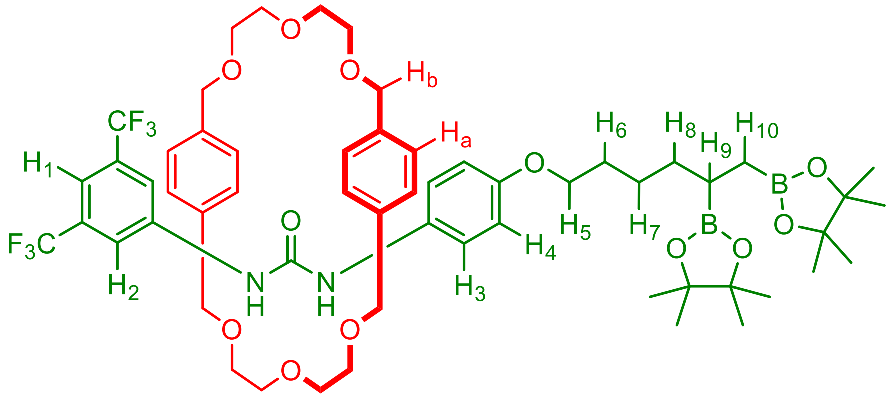

15

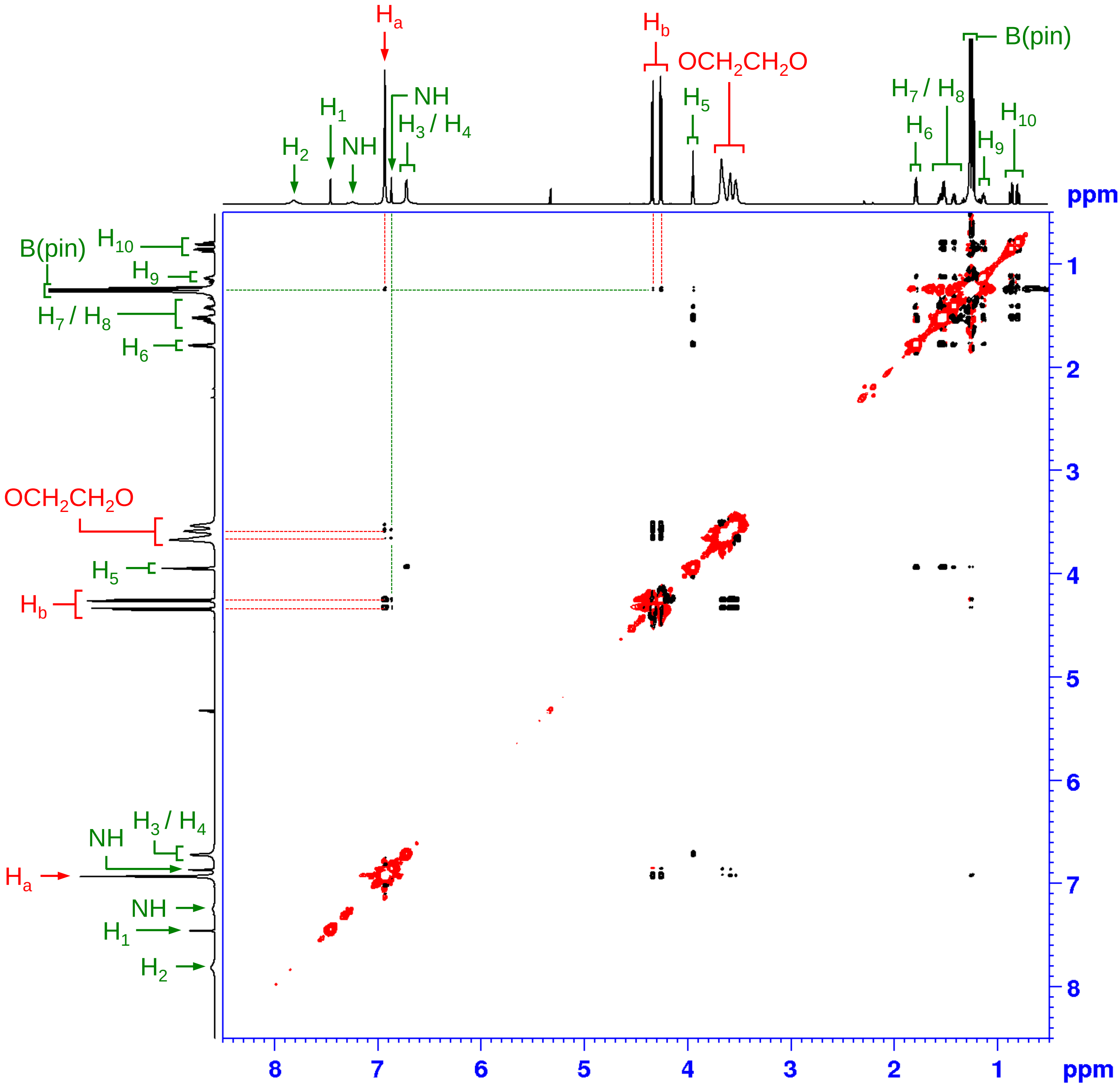

Figure S52. 2D COSY Spectrum (500 MHz / $CDCl_3$ / 298 K) of **17**·TFPB

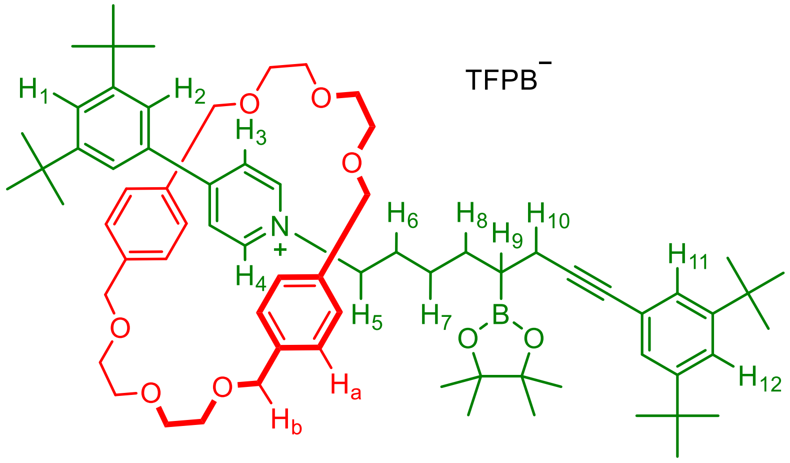

17•TFPB

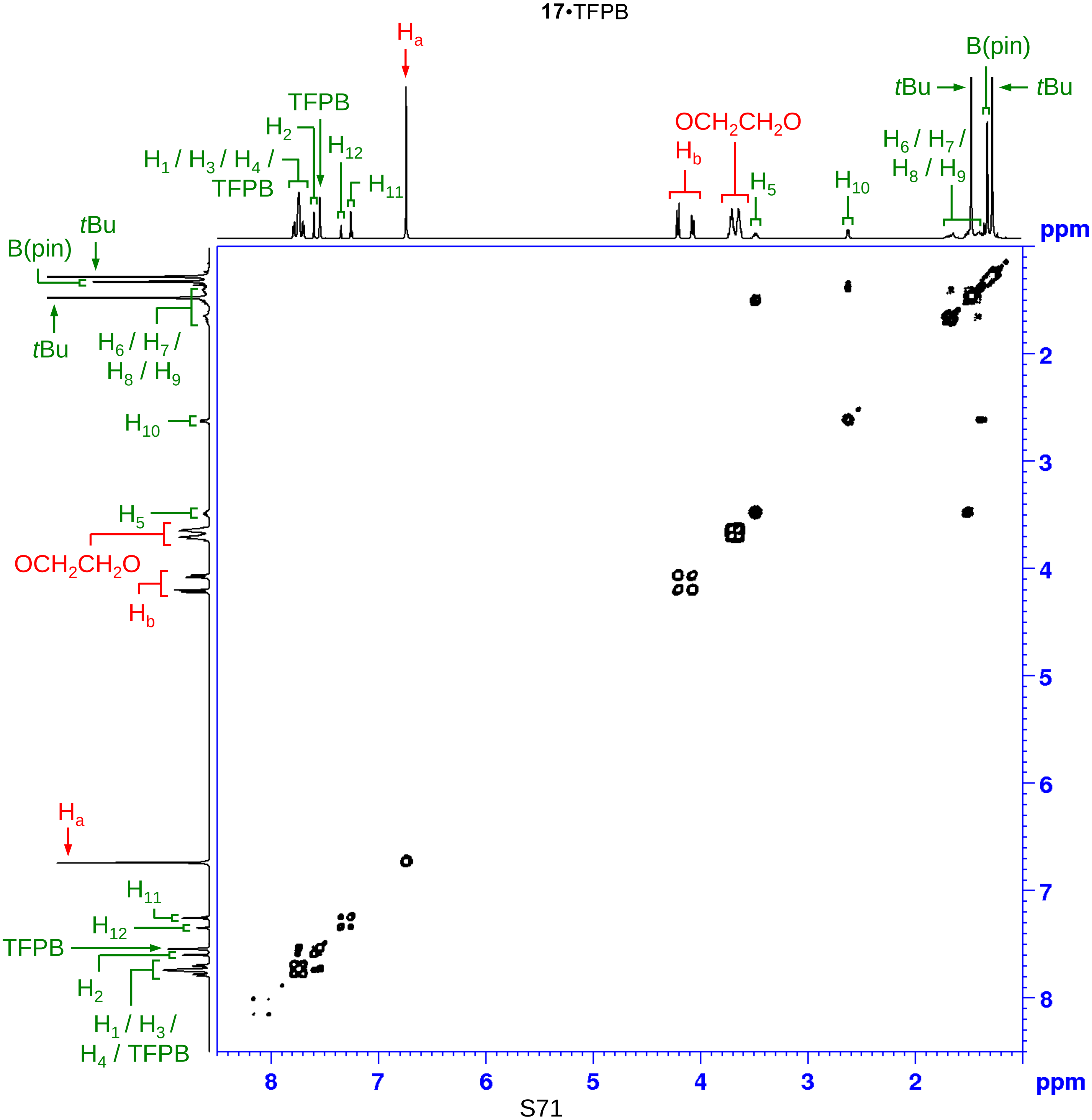

Figure S53. 2D ROESY Spectrum (500 MHz / $CDCl_3$ / 298 K) of **17**·TFPB

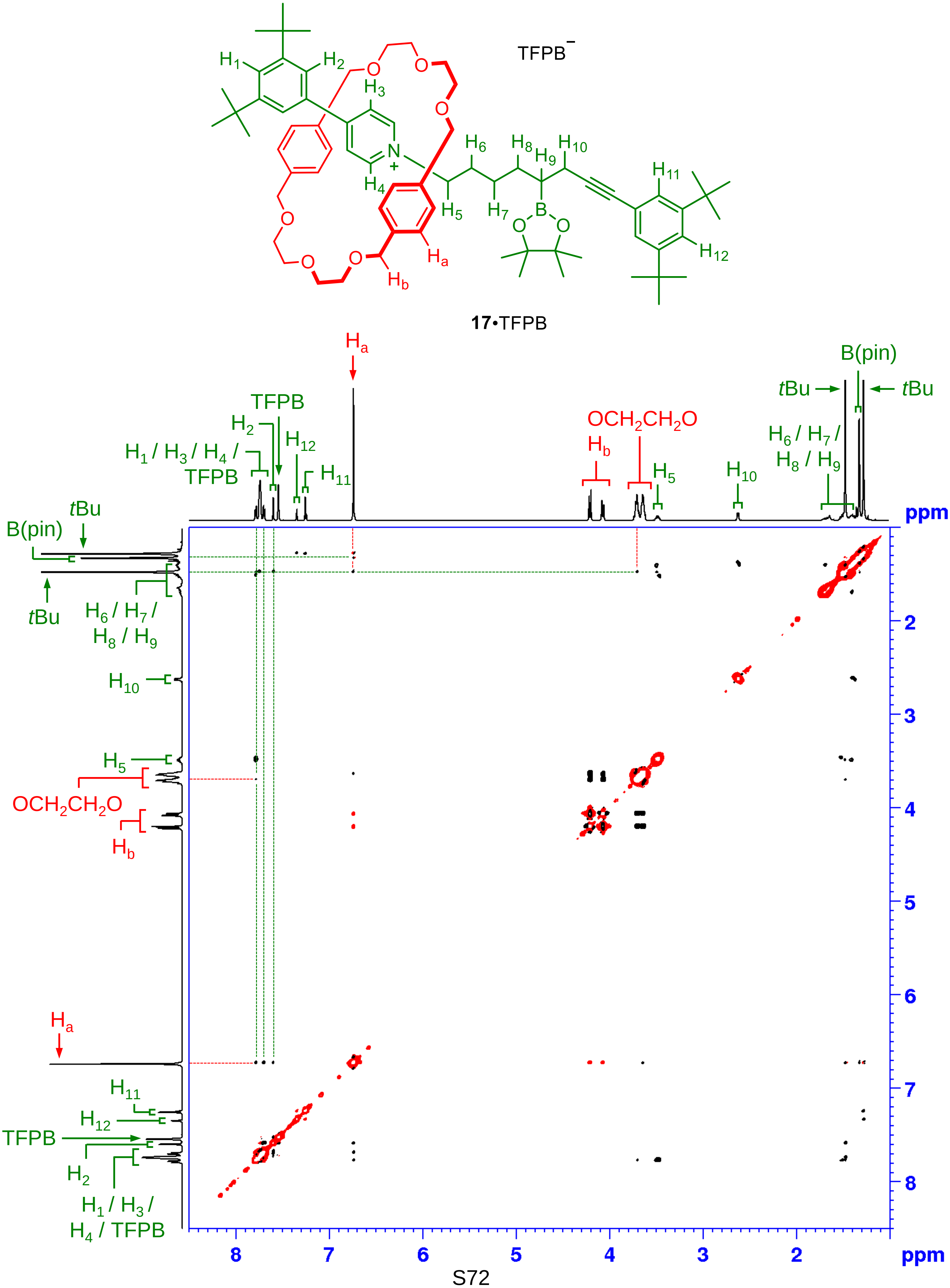

Figure S54. 2D COSY Spectrum (800 MHz / $CDCl_3$ / 298 K) of **18**·TFPB

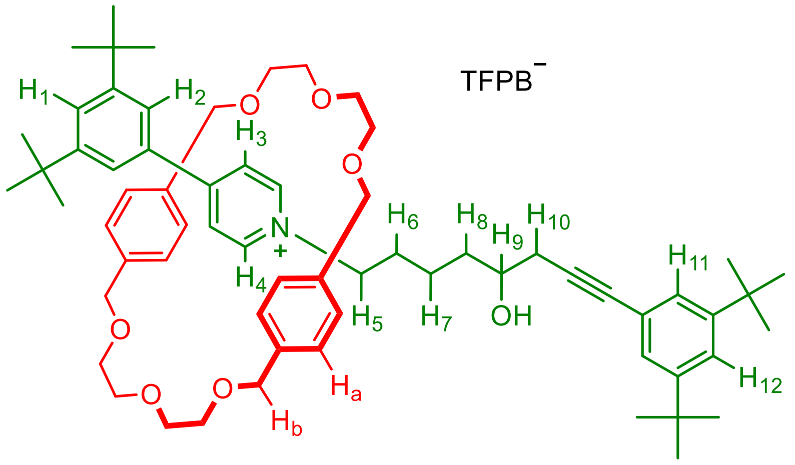

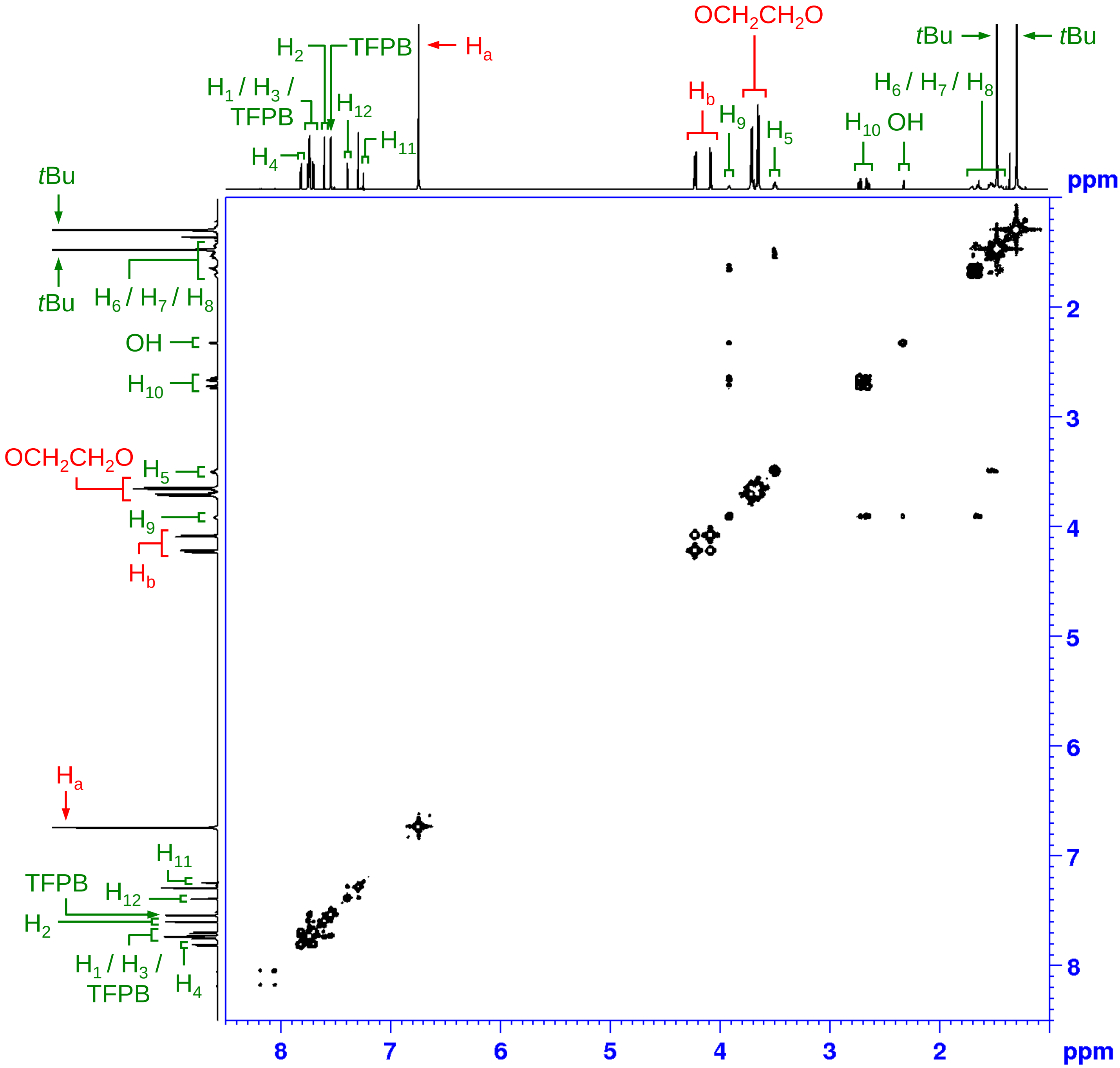

Figure S55. 2D ROESY Spectrum (800 MHz / $CDCl_3$ / 298 K) of **18**·TFPB

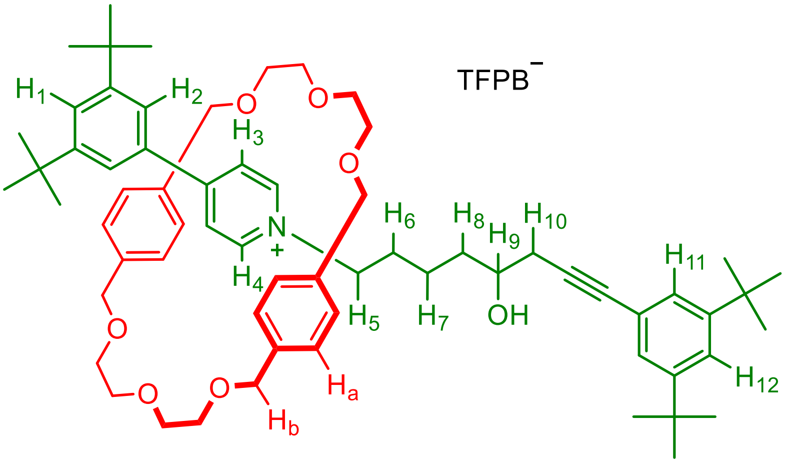

18•TFPB

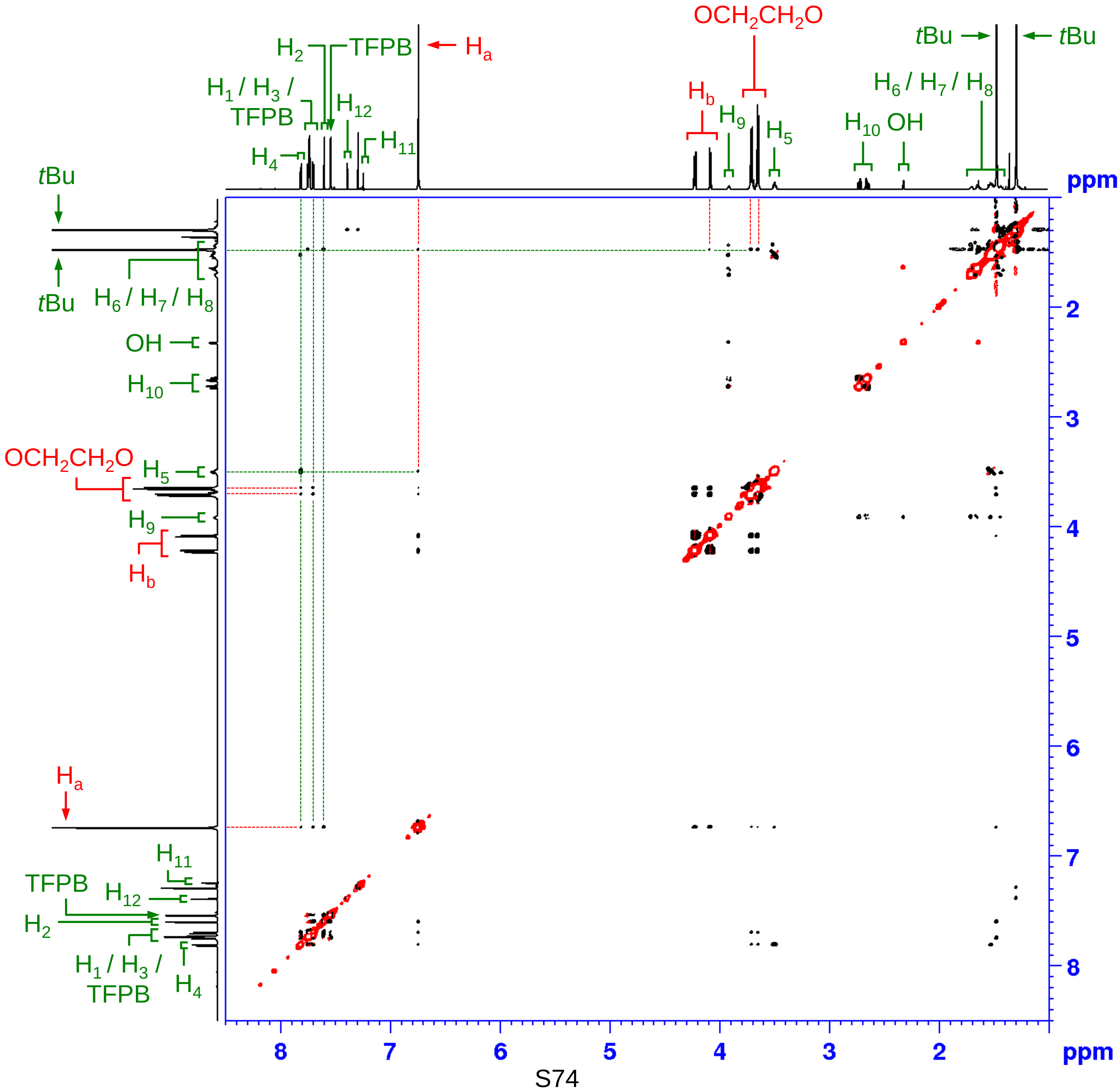

Figure S56. 2D COSY Spectrum (500 MHz / $CDCl_3$ / 298 K) of **19**

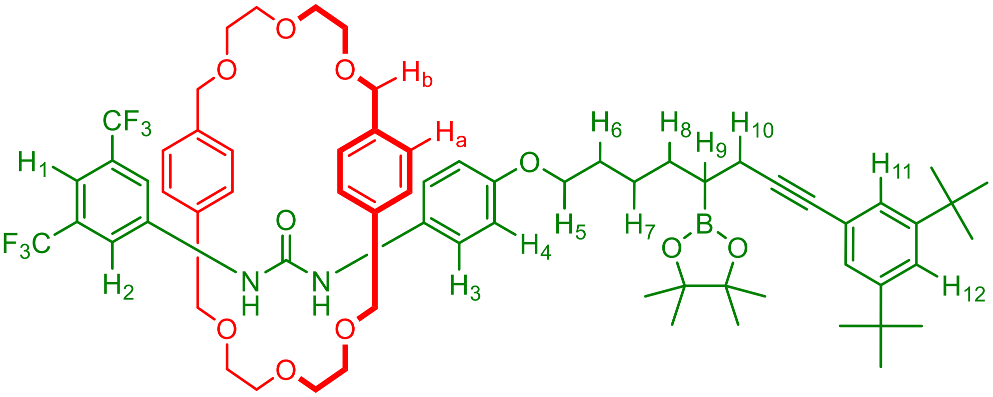

19

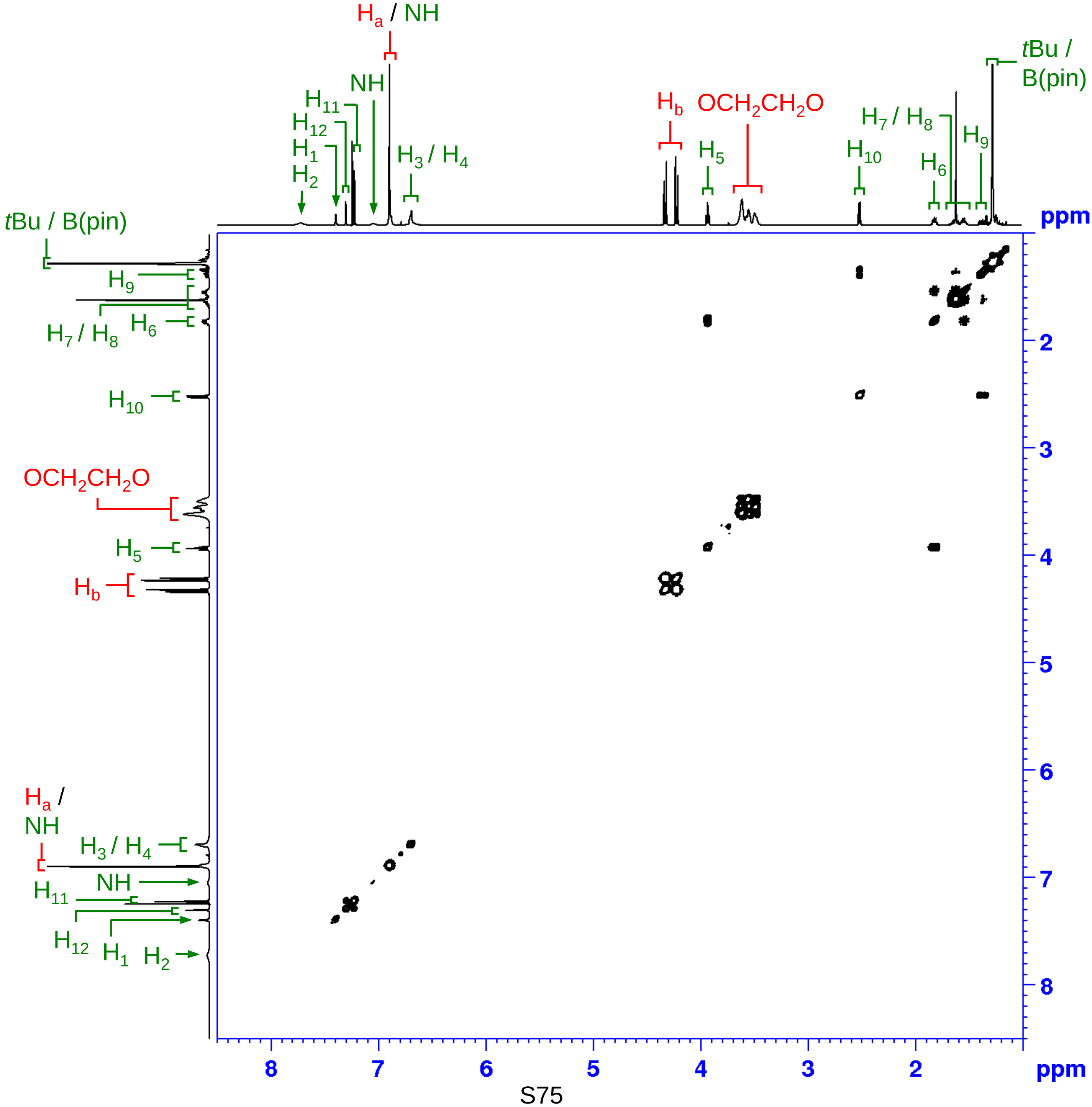

Figure S57. 2D ROESY Spectrum (500 MHz / $CDCl_3$ / 298 K) of **19**

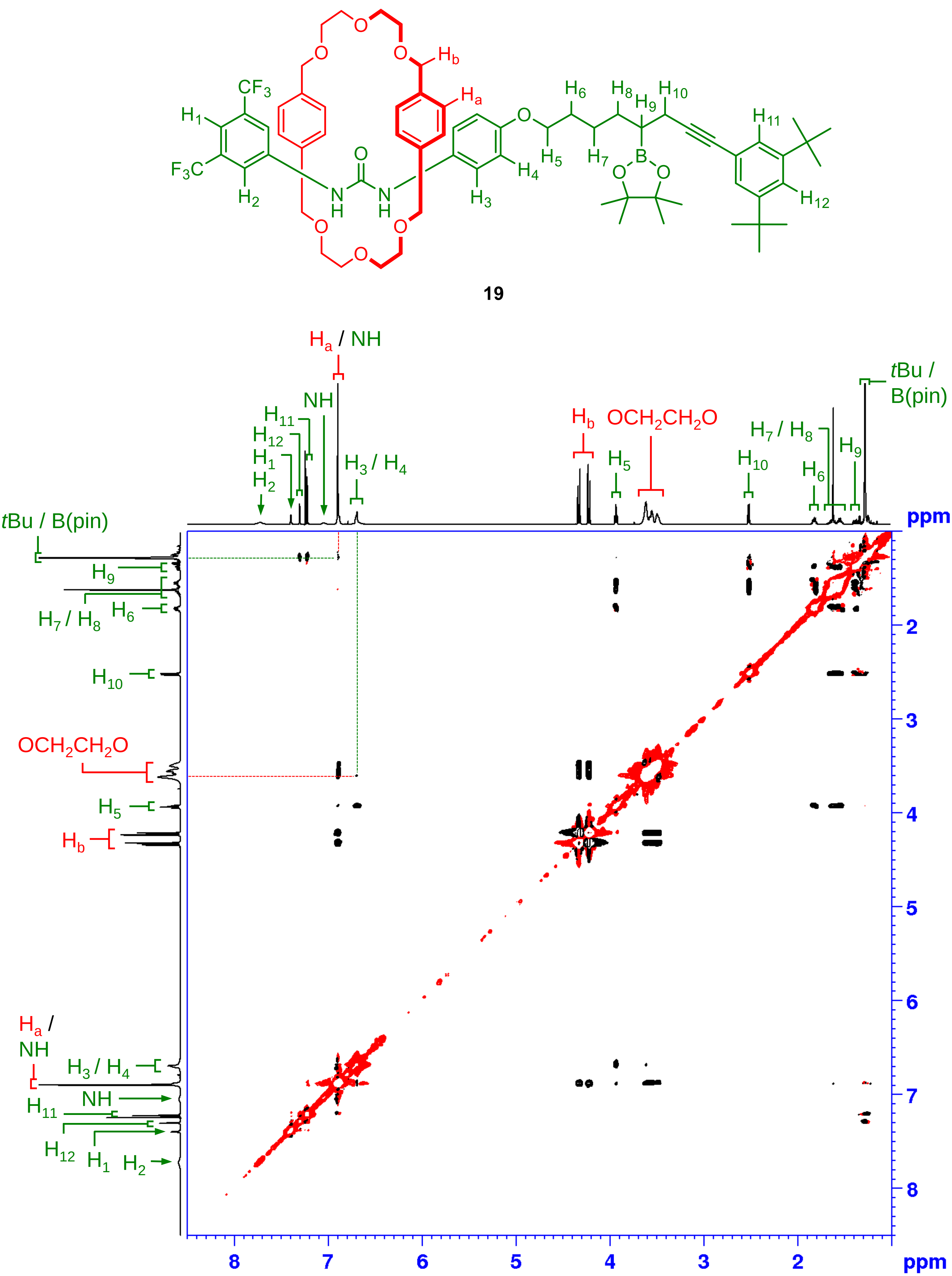

Figure S58. 2D COSY Spectrum (500 MHz / $CDCl_3$ / 298 K) of **20**

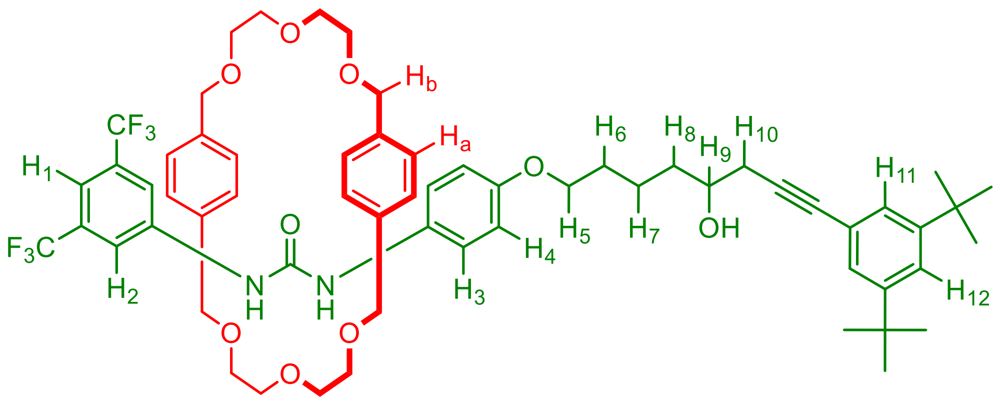

20

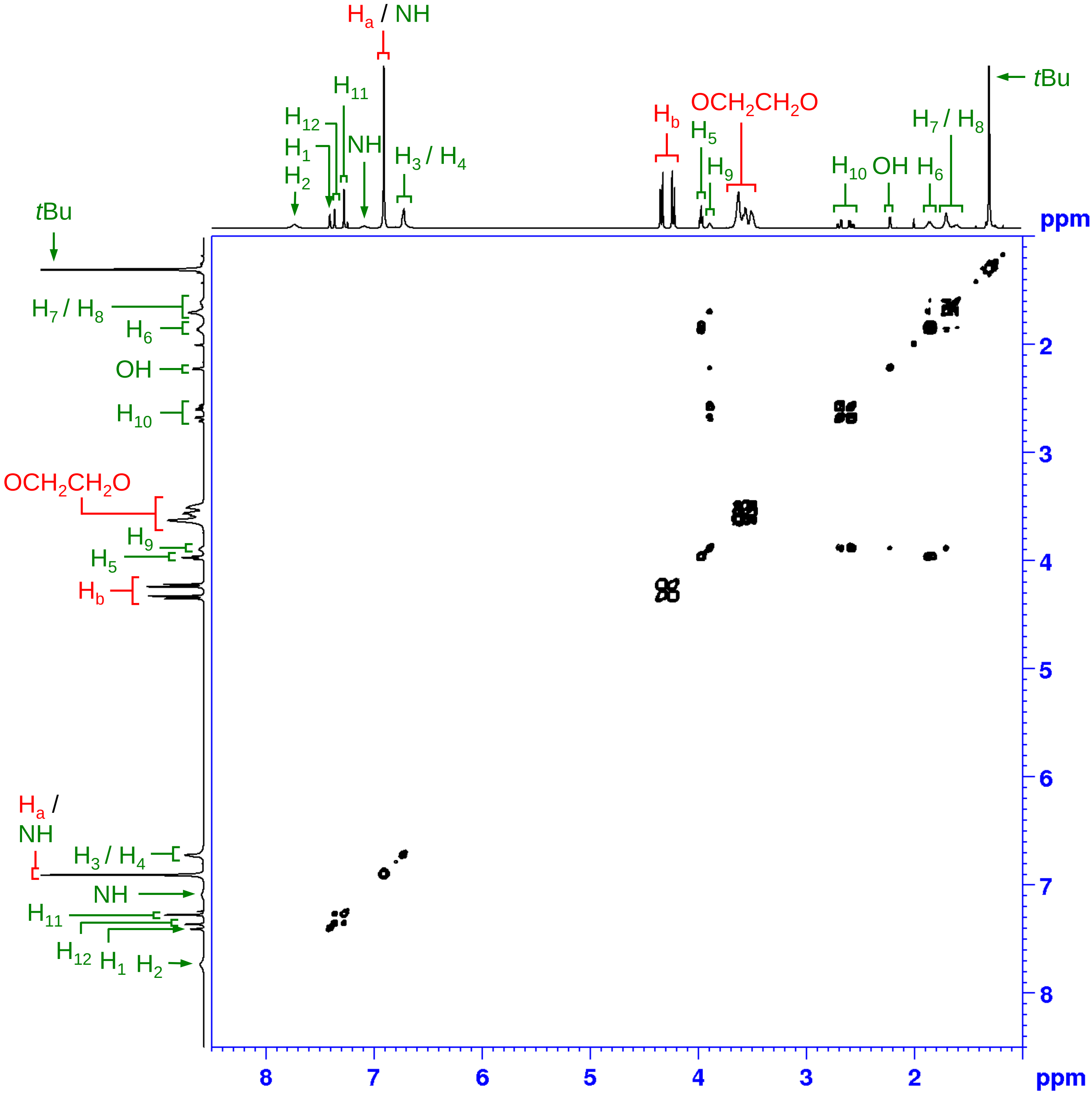

Figure S59. 2D ROESY Spectrum (500 MHz / $CDCl_3$ / 298 K) of **20**

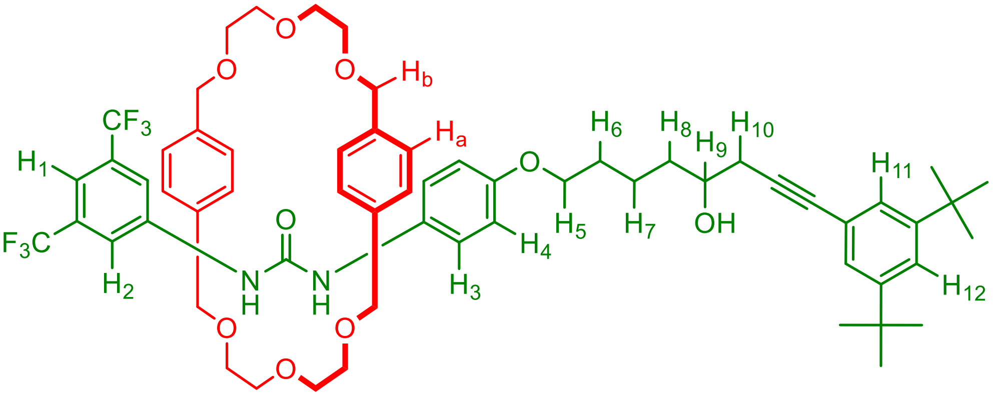

20

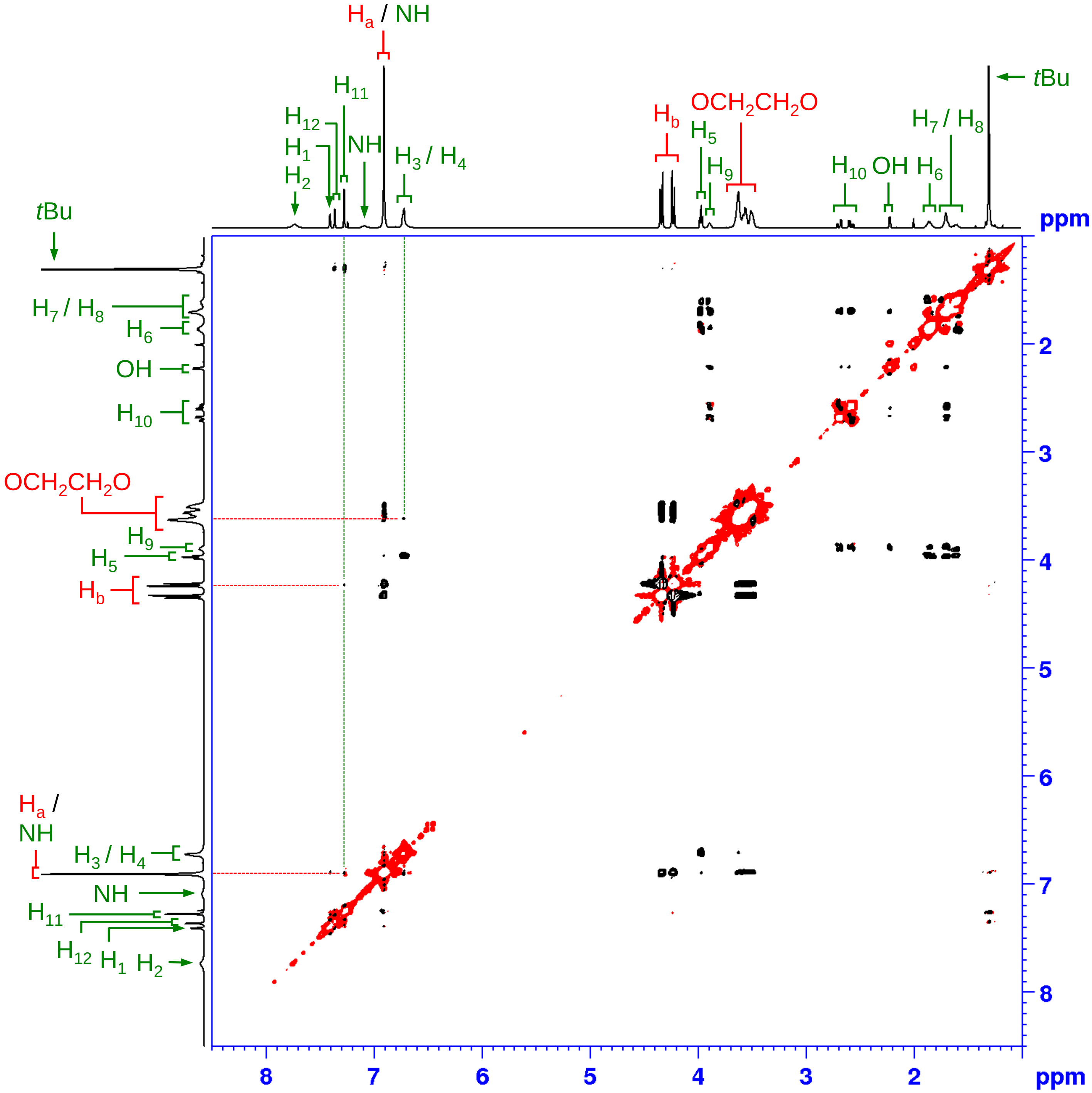

Figure S60. ^{1}H NMR Spectra (400 MHz / toluene-d_8 / 40 mM / 298 K) of the 9-BBN hydroboration of **1**·TFPB, performed with or without BPX26C6.

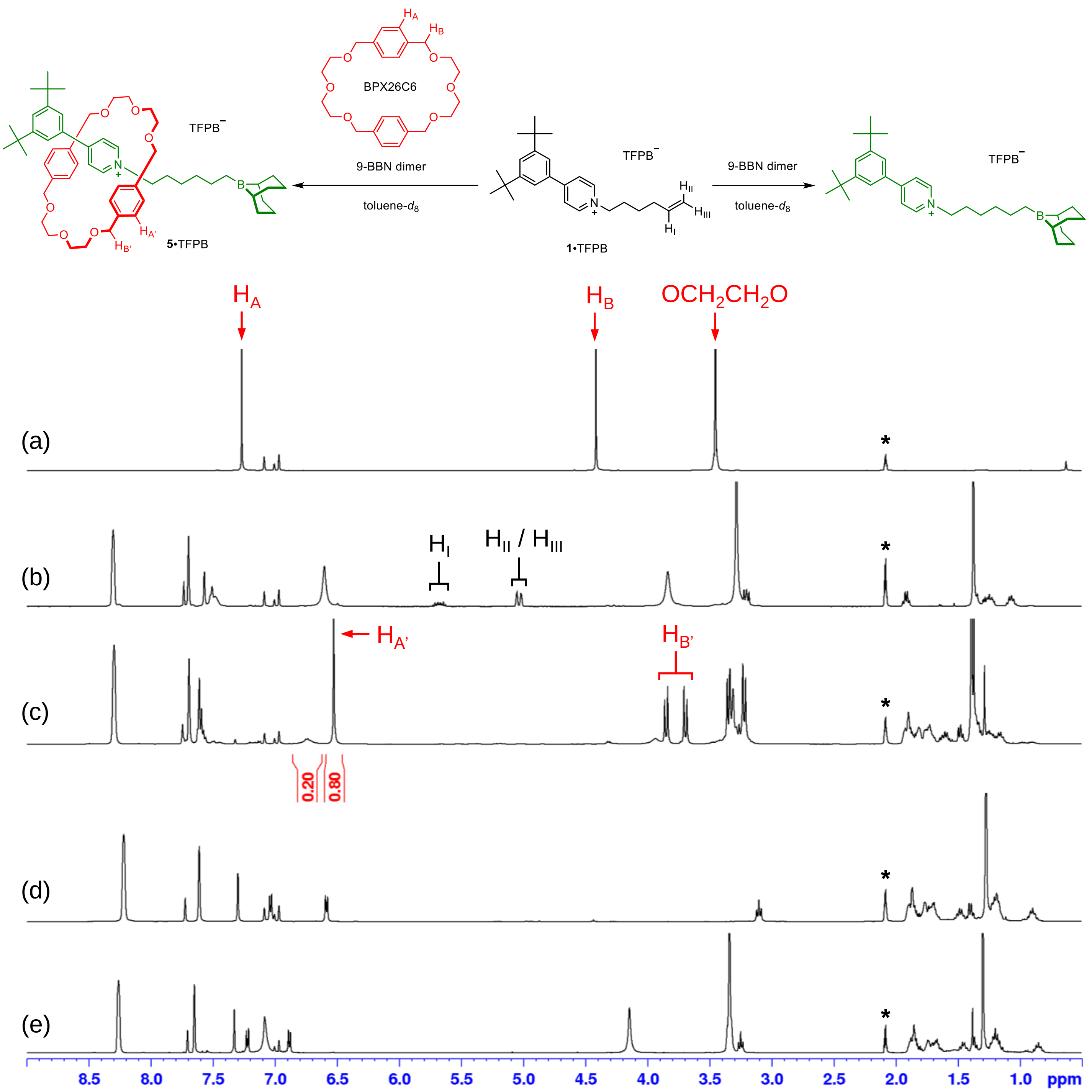

Each sample was obtained by diluting the corresponding reaction mixture (400 mM, in toluene-d_8) to 40 mM with toluene-d_8. Asterisks: signals from residual solvent.

(a) BPX26C6 (1 equiv.).

(b) mixing **1**·TFPB (1 equiv.) and BPX26C6 (1 equiv.).

(c) mixing (b) and 9-BBN dimer (0.55 equiv.), and heating at 60 °C for 16 h.

(d) mixing **1**·TFPB (1 equiv.) and 9-BBN dimer (0.55 equiv.), and heating at 60 °C for 16 h.

(e) mixing (d) and BPX26C6 (1 equiv.), and heating at 60 °C for 16 h.

Figure S61. ORTEP representation of [2]rotaxane **7**·TFPB showing 50% probability atomic displacement parameters. Anions, hydrogen atoms and solvent molecules have been omitted for clarity

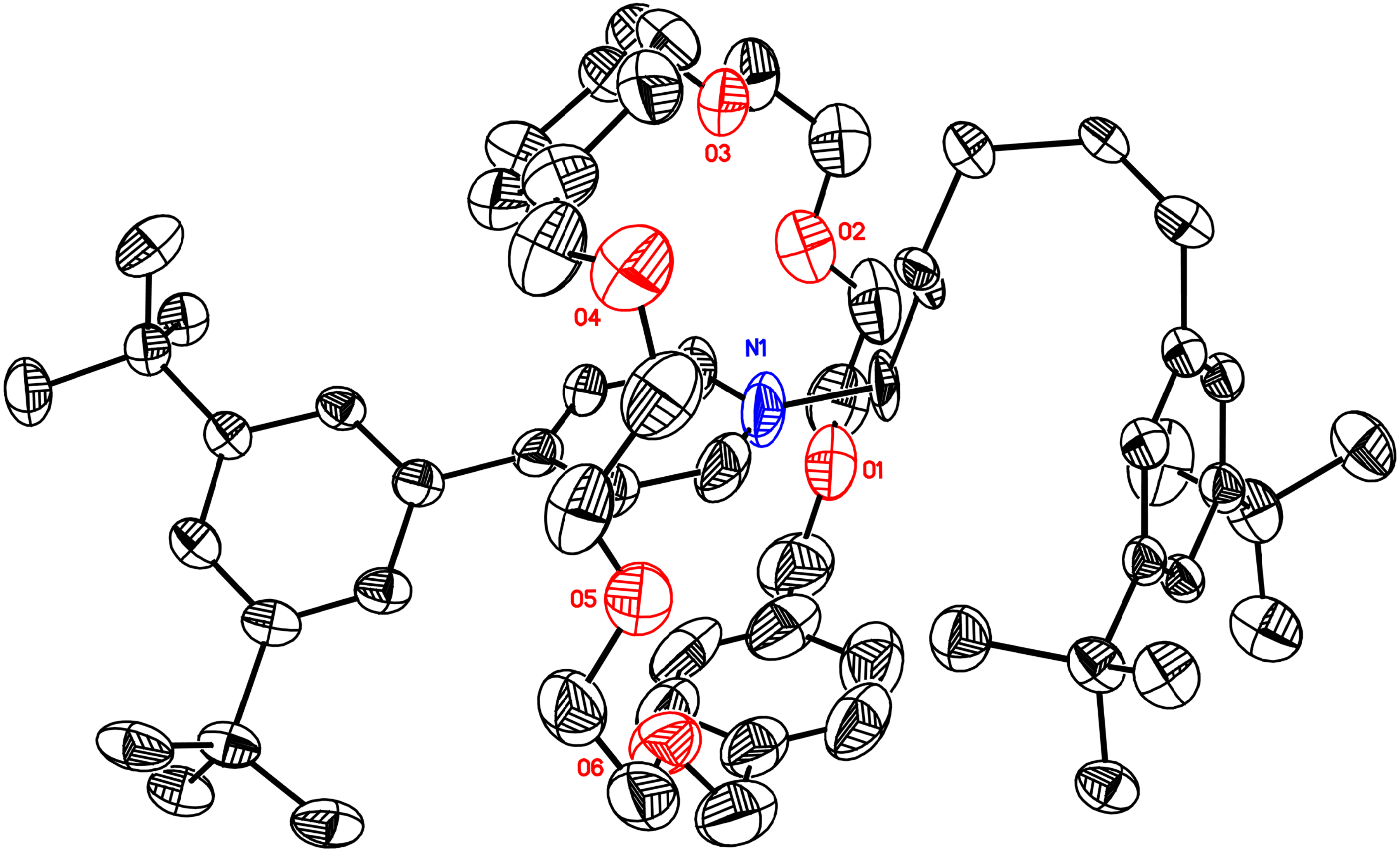

Table S1. Crystal data and experimental details for [2]rotaxane **7**·TFPB

	Crystal data	
Empirical formula	C95.25 H102.50 B Cl0.50 F24 N O6	
Formula weight	1841.81	
Crystal system	Monoclinic	
Space group	$P2_1/c$	
Unit cell dimensions	a = 21.4755(6) Å	α= 90°.
	b = 22.8618(7) Å	β= 119.5623(9)°.
	c = 21.3448(6) Å	γ = 90°.
Volume	9115.4(5) $Å^3$	
Z	4	
F(000)	3834	
Density (calculated)	1.342 Mg/m^3	
Wavelength	1.54178 Å	
Cell parameters reflections used	9334	
Theta range for Cell parameters	3.07 to 68.22°.	
Absorption coefficient	1.130 mm^{-1}	
Temperature	100(2) K	
Crystal size	0.250 x 0.150 x 0.150 mm^3	
	Data collection	
Diffractometer	Bruker D8 VENTURE, PhotonIII_C28	
Absorption correction	Semi-empirical from equivalents	
Max. and min. transmission	1.0000 and 0.8967	
No. of measured reflections	130522	
No. of independent reflections	16655 [R(int) = 0.0335]	
No. of observed [I>2_igma(I)]	15264	
Completeness to theta = 67.679°	99.9 %	
Theta range for data collection	2.365 to 68.297°.	
	Refinement	
Final R indices [I>2sigma(I)]	R1 = 0.1010, wR2 = 0.2623	
R indices (all data)	R1 = 0.1061, wR2 = 0.2666	
Goodness-of-fit on F^2	1.039	
No. of reflections	16655	
No. of parameters	1368	
No. of restraints	816	
Largest diff. peak and hole	1.534 and -0.831 e.$Å^{-3}$	
